# Consensus Integration of Multiomics Data With Machine Learning Algorithms Reveals Heterogeneous Molecular Subtypes and Enables Personalized Treatment Strategies for Hepatocellular Carcinoma

**DOI:** 10.1155/humu/9967779

**Published:** 2025-11-24

**Authors:** Zhipeng Jin, Kun Fang, Xue Zhang, Mengying Song, Hong Jiang, Yefu Liu

**Affiliations:** ^1^Department of Hepatopancreatobiliary Surgery, Liaoning Cancer Hospital & Institute, Shenyang, Liaoning, China; ^2^Central Laboratory, Liaoning Cancer Hospital & Institute, Shenyang, Liaoning, China; ^3^Department of Operation Room, Liaoning Cancer Hospital & Institute, Shenyang, Liaoning, China

**Keywords:** hepatocellular carcinoma, immunotherapy, machine learning, molecular subtype, multiomics, prognosis, tumor microenvironment

## Abstract

**Background:**

Cancers are characterized by high heterogeneity. This study seeks to identify the factors driving hepatocellular carcinoma (HCC) heterogeneity to aid in prognostic stratification and inform personalized treatment approaches.

**Methods:**

We used a computational pipeline to integrate multiomics data from HCC patients, applying 10 clustering algorithms. These results were combined with a machine learning framework to identify high-resolution molecular subtypes (MSs) and to create a robust molecular subtype–related risk score (MSRRS). Subsequent integrated bioinformatics algorithms further analyzed the heterogeneity of HCC at the level of molecular pathways, therapeutic response, and tumor microenvironment, thereby assessing potential clinical value.

**Results:**

Through multiomics clustering, we identified two heterogeneous MSs associated with prognosis, with MS2 exhibiting a more favorable prognostic outcome. Subsequently, we applied bootstrap resampling-based univariate Cox regression and Boruta algorithm to screen for more clinically relevant genes from the marker genes of each MS. Next, we benchmarked seven survival-related machine learning algorithms for overall survival (OS) using nested cross-validation. The hyperparameter-tuned Ridge survival model outperforms other tuned models and was therefore used to develop a robust MSRRS. MSRRS demonstrated superior performance in predicting patient OS in multiple independent HCC cohorts. Downstream analysis suggested that MSRRS has the potential to guide individualized targeted therapy, chemotherapy, and immunotherapy for HCC and to assess the tumor microenvironment. Pathway enrichment analysis identified the cell cycle as a crucial driver of heterogeneity differences between the two subtypes. Finally, we confirmed that KIF2C may be the most central MSRRS gene and demonstrated by in vitro experiments that KIF2C could promote G2/M transition in HCC cells by targeting CDK1/CCNB1/PLK1 signaling.

**Conclusion:**

The novel MSs and robust MSRRS we identified effectively exposed the heterogeneity of HCC and have the potential to predict prognosis and guide individualized precision therapy.

## 1. Introduction

Hepatocellular carcinoma (HCC) is the main type of primary liver cancer (PLC), accounting for more than 80% of all PLC cases, and also one of the most aggressive malignant tumors [1]. The etiology of HCC is complex, yet relatively well defined. In East Asia and Africa, higher rates of chronic hepatitis B virus (HBV) infection and aflatoxin B1 (AFB1) exposure contribute to higher HCC morbidity and mortality [2]. In fact, cirrhosis of any etiology is a risk factor for HCC, including chronic alcohol consumption, diabetes or obesity-related nonalcoholic steatohepatitis, and hepatitis C virus (HCV) infection, which are also responsible for the development of HCC in some developed countries [3]. Recent years have seen significant advancements in systemic therapy due to extensive basic research and clinical trials, particularly in the areas of multikinase inhibitors, antiangiogenic drugs, and immune checkpoint inhibitors (ICIs). These advancements have notably improved the prognosis for HCC patients, providing the opportunity for radical surgery for many previously ineligible cases. Despite the availability of drug therapy, a majority of patients still do not achieve satisfactory clinical outcomes due to its limited effectiveness [4].

The extensive heterogeneity, encompassing genetic and immunological variations, stands as a significant hurdle in achieving effective treatment outcomes for HCC [5, 6]. Hence, it is imperative to comprehensively characterize the tumor and establish tailored treatment strategies. The advent of high-throughput sequencing technologies in the 21st century has revolutionized the analysis of a species' transcriptome and genome, enabling a profound and holistic examination. Many studies leveraging high-throughput data have significantly enhanced our comprehension of human disease [7–10]. Numerous molecular characteristics of HCC have been increasingly revealed, leading to the identification of two primary subclasses that differ significantly in various aspects, including etiology, prognosis, and clinical indicators [11]. Unfortunately, the current molecular subtypes (MSs) of HCC are predominantly rooted in traditional molecular characteristics, such as high, intermediate, and low differentiation, Tumor Grades I–IV, or proliferation and nonproliferation subtypes [4, 11], thus limiting their ability to effectively guide the advanced personalized treatment of HCC, particularly in terms of the utilization of tyrosine kinase inhibitors (TKIs) and ICIs. Therefore, there is a need for improved HCC classifications that can accurately identify tumor heterogeneity and aid in making informed clinical treatment decisions.

Here, we combined multiomics data to develop an integrated consensus subtype of HCC. Subsequently, we employed a robust machine learning framework to construct the molecular subtype–related risk score (MSRRS). Validation of multiple independent cohorts demonstrated the significant prognostic value of MSRRS and its robustness in predicting drug sensitivity. Finally, we examined the potential factors driving the heterogeneity of HCC associated with the MSRRS. Our findings provide an important reference for enhancing the precise stratification and individualized treatment of HCC.

## 2. Materials and Methods

### 2.1. Multiomics Data Collection and Processing

Omics data for the TCGA-LIHC cohort were obtained from the GDC Data Portal (https://portal.gdc.cancer.gov/). The mature miRNA was identified using the miRBaseVersions.db R package. Mutation data were processed using the maftools package. For DNA methylation data, we chose probes targeting promoter CpG islands. Samples with complete omics data and clinical data were used for subsequent analyses. For external validation cohorts, the RNA-seq data and clinical data for the LIRI-JP cohort were downloaded from the ICGC Data Portal (https://dcc.icgc.org/). Gene expression microarray data from GSE14520 and GSE116174 cohorts were downloaded from the GEO database (http://www.ncbi.nlm.nih.gov/geo/). For immunotherapy cohorts, the RNA-seq and clinical data for the GSE78220 (melanoma), GSE135222 (non–small cell lung carcinoma), and GSE91061 (melanoma) cohorts were downloaded from the GEO database. Transcriptomic and matched clinical data for the IMvigor210 (urothelial carcinoma) cohort were collected from http://research-pub.gene.com/IMvigor210CoreBiologies. RNA-seq data was converted to transcripts per million (TPM) format, and the mean value was utilized to represent the expression level of genes matched to multiple probes. The sva R package was employed to remove batch effects across multiple cohorts, followed by principal component analysis (PCA) to examine the data before and after batch effect correction. scRNA-seq data for the GSE149614 cohort was downloaded from the GEO database. Another single-cell dataset was downloaded from the Mendeley Data (https://data.mendeley.com/datasets/skrx2fz79n/1). Data were processed using the Seurat V4.0 R package. Quality control procedures used the following filter criteria: 200 < nFeature < 7000 and percent.mt < 20%. We first performed log normalization to standardize the merged data and found hypervariable genes through the FindVariableFeatures function. Subsequently, the dimensionality of the scRNA-seq data was diminished through PCA. Then, we removed batches of samples using the harmony method through the FindIntegrationAnchors function and integrated samples using the IntegrateData method. Uniform manifold approximation and projection (UMAP) dimensionality reduction was performed on all cells with the RunUMAP function. Finally, cell type annotation was performed by the method described in previous studies [12, 13]. All raw data and patient information involved in this study were obtained from publicly available databases. Ethical approval has been obtained for all research subjects contained in these databases, and users can freely access and download relevant data for research purposes. Therefore, our study does not involve any ethical concerns or conflicts of interest.

### 2.2. Multiomics Consensus Ensemble Analysis

First, we used the getElites function from the MOVICS package to identify gene features [14]. For continuous variables, we utilized the mad method to select the Top 1500 genes exhibiting the highest variation. Subsequently, we employed the Cox method to identify prognostic genes with a significance level of *p* < 0.05. In the case of gene mutation data, we initially utilized the oncoPrint function of the maftools package to identify the Top 5000 genes exhibiting the highest mutation rate. We further refined the selection based on mutation frequency by setting the method parameter to freq in order to identify the Top 5% most frequently mutated genes. The resulting data derived from each dimension were included for further analysis.

Next, we used the getClustNum function from the MOVICS package, which combines clustering prediction index, Gaps-statistics, and silhouette score to estimate the number of subgroups [15, 16]. Taking into account the low level of noise, the significant amount of important information, and our prior understanding of HCC heterogeneity, we made the decision to classify the samples into two major subclasses. Subsequently, we utilized the getMOIC function input 10 clustering algorithms for clustering analysis. We then utilized the getConsensusMOIC function to combine the results from various methods, thereby strengthening the clustering process. The distance parameter was set to Euclidean, and the linkage parameter was set to average.

Finally, to enhance the robustness of subtyping, we initially confirmed the results by employing subtype-specific biomarkers in the validation cohort. Subsequently, we compared the consistency of consensus clustering with the nearest template prediction (NTP) and partition around medoid (PAM) classifier [14, 17]. The Top 100 upregulated genes in each subtype were screened for subsequent analyses. The clusterProfiler package was used to identify and visualize the disease ontology (DO) terms enriched for marker genes.

### 2.3. Assessment of the Molecular Landscape

We first assessed the activity of specific gene sets in each HCC sample through gene set variation analysis (GSVA) [18]. The annotation files of 50 hallmark gene sets (h.all.v7.5.1.symbols.gmt) and 186 KEGG pathways (c2.cp.kegg_legacy.v2023.2.Hs.symbols.gmt) were obtained from the MSigDB (http://www.gsea-msigdb.org/gsea/downloads.jsp). Then, we evaluated the expression of 23 transcription factors (TFs) and potential regulators associated with cancer chromatin remodeling collected from previous studies [17, 19]. Finally, we assessed the differences in tumor microenvironment (TME) between the two HCC subtypes, including the expression of immune checkpoint genes, the activity of immune-related pathways, and the abundance of immune cell types. The activity of immune functional pathways was estimated by single-sample gene set enrichment analysis (ssGSEA) [20, 21]. The abundance of immune cells in HCC samples was estimated by six independent methods [22–27].

### 2.4. Construction of a Consensus Machine Learning–Driven Prognostic Signature

Based on the 200 marker genes described previously, univariate Cox analysis identified 93 MS-related prognostic genes (*p* < 0.05). Subsequently, we implemented a bootstrap resampling strategy with 1000 iterations, each time randomly selecting 80% of patients while preserving cohort characteristics. Genes demonstrating consistent statistical significance (*p* < 0.05) in over 900 iterations were retained for subsequent analysis. Furthermore, we refined feature selection using the Boruta algorithm (configured with ntree = 1000 and maxRuns = 1000 parameters) to identify genes demonstrating significant associations with overall survival (OS). This method systematically evaluates feature importance through comparison with randomly permuted shadow features, ensuring selection of variables with true predictive capacity. Based on the identified genes, seven machine learning algorithms were evaluated using nested cross-validation (CV) in the training cohort. We tuned the hyperparameters of each algorithm (Table S1) using the inner fivefold CV and evaluated the performance of the best tuned models using the outer tenfold CV. In each fold, the same ratio of alive and dead cases was maintained. The performance evaluation of the MSRRS was carried out by comparing the averages of C-index and integrated brier score (IBS). A similar machine learning benchmark for constructing a gene signature was described in a previous study [28].

### 2.5. Identification of Pathways Associated With MSRRS

GSEA was performed to identify pathways related to MSRRS [20]. The number of permutations was set to 1000. The value of NES indicates the extent of enrichment. Subsequently, the correlation of MSRRS with GSVA scores for 50 hallmark gene sets was assessed. Finally, we obtained the interacting molecules closely related to the 10 target genes through the GeneMANIA database (https://genemania.org/search/homo-sapiens//).

### 2.6. In Silico Prediction of Therapeutic Targets and Agents

Proteins with significant correlation to MSRRS may have potential therapeutic implications for patients with different risk groups. However, the majority of human proteins are considered undruggable due to a lack of apparent active sites for small molecule compounds to bind to, or because they are located within cells that are inaccessible to biological agents. Therefore, we collected 2249 druggable targets from a previous publication (Table S2) [29]. Expression profile data for human cancer cell lines (CCLs) were obtained from the CCLE database (https://sites.broadinstitute.org/ccle/) [30]. The CERES scores of genome-scale CRISPR knockout screens were acquired from the DepMap database (https://depmap.org/portal/). A lower CERES score indicates a higher likelihood that the gene is essential in cell growth and survival of a given CCL. The proteins with significant positive correlation (*r* > 0.4) with MSRRS and the genes with significant negative correlation (*r* < −0.4) between CERES score and MSRRS were screened as potential therapeutic targets.

We also applied various databases and algorithms to estimate the sensitivity of clinical samples to anticancer drugs. Drug sensitivity data for CCLs were retrieved from the CTRP database (https://portals.broadinstitute.org/ctrp.v2.1/) and PRISM database (https://www.theprismlab.org/). Both databases provide area under the dose-response curve (AUC) values as a measure of drug sensitivity, with lower AUC values indicating increased sensitivity to treatment. We identified drugs with lower AUC in the high MSRRS group (log2FC > 0.2). We also assessed the correlation between the AUC of drugs and MSRRS and screened for drugs whose AUC was significantly negatively correlated with MSRRS (*r* < −0.25). Intersection of the drugs screened above as potential therapeutic agents. Finally, we used the prophetic package to estimate the IC_50_ of specimens to drugs based on the GDSC database (https://www.cancerrxgene.org/) in order to validate the robustness of the aforementioned method and explore new potential therapeutic agents [31].

### 2.7. Comprehensive Analysis of TME Characterization and Immunotherapy Response

Leveraging the IOBR package [32], we systematically aggregated numerous previously published signatures associated with TME. Subsequently, a standardized method was applied to calculate the enrichment score for each sample, enabling a comprehensive analysis of the immunological variances between specimens. Then, we applied the TIP algorithm to evaluate the differences in immunoreactivity between HCC specimens at different phases in the cancer-immunity cycle [33]. Furthermore, we analyzed variations in the multiomics characteristics of various immunomodulators between samples. Finally, we utilized the TIDE algorithm and the ImmuCellAI algorithm to predict sample response to ICI treatment and evaluated the extent of immune dysfunction and exclusion [34–36]. And the correlation between MSRRS and ICI treatment response has been validated in multiple immunotherapy cohorts.

### 2.8. Cell Culture and Transfection

Human HCC-derived JHH7 and Huh7 cells were obtained from the ATCC. Cells were cultured with DMEM (Gibco, Life Technologies, United States) with 10% FBS (Gibco, Life Technologies, United States) and 1% penicillin–streptomycin at 37°C in a humidified incubator with 5% CO_2_ atmosphere. For gene silencing by RNA interference, siRNAs against KIF2C were transiently transfected into cells using the Lipofectamine 3000 (Lipo3000) based on the manufacturer's instructions. Cells were harvested after transfection for 2–3 days and used for further experiments.

### 2.9. Real-Time Quantitative PCR

Human KIF2C gene amplification was performed using sequence-specific primers (Sangon Biotech, Shanghai) with sequences detailed in Table S3. Quantitative PCR analysis was carried out in 20 *μ*L reactions containing 100 ng cDNA template, employing TaqMan Universal PCR Master Mix (Applied Biosystems) on a Roche LightCycler 96 real-time PCR system. The thermal profile consisted of initial denaturation at 95°C for 10 min, followed by 40 cycles of 95°C for 15 s (denaturation) and 60°C for 60 s (annealing/extension).

### 2.10. Flow Cytometry Assay of Cell Cycle

Transfected cells were collected, and the number of cells was 10 × 10^6^. The cells were washed twice with cold PBS and were suspended in a 15-mL centrifuge tube with 500 *μ*PBS. Add 5 mL cold ethanol and fix overnight at 4°C. The 5 × 10^6^ cells were taken and centrifuged 1000 × g in a 15-mL conical centrifuge tube for 10 min. The supernatant was discarded, and the cells were collected. Rinse the cells twice more with cold PBS. Centrifuge at 1000 × g for 10 min, discard supernatant, and collect cells. The cell cycle analysis kit (Beyotime, Shanghai, China) was used to stain propyridine iodide (PI), and the results were detected by flow cytometry. The excitation wavelength is 488 nm. DNA content and light scattering were analyzed by software.

### 2.11. Western Blotting

HCC cells were inoculated into a six-well plate at a density of 70%–80%. After the cells adhered to the wall, the total protein of the cells was extracted with RIPA lysate (P0013C, Beyotime Biotechnology, China), and the protein concentration was detected. SDS-PAGE was used to detect the expression of related proteins. Incubate the strips in a dilution of the first antibody and incubate overnight at 4°C (PLK1: ab189139, Abcam, United Kingdom; cyclin-dependent kinase 1 [CDK1]: ab133327, Abcam, United Kingdom; KIF2C: 28372-1-AP, Proteintech, China; alpha tubulin: 11224-1-AP, Proteintech, China; CCNB1: 4138, CST, America). On the second day, TBST was used to clean the strips four times for 5 min each time. The second antibody diluent was prepared using the same method and incubated at room temperature for 1–2 h. The strips were again washed with TBST four times for 5 min each time. Finally, images were collected using the multifunctional chemical imaging instrument.

### 2.12. Statistical Analysis

All statistical analyses were conducted with R software (Version 4.3.2). Continuous variables were analyzed using Student's *t*-test for normally distributed data, while nonparametric alternatives (Wilcoxon rank sum test for two-group comparisons and Kruskal–Wallis test for multigroup comparisons) were applied to nonnormally distributed datasets. Associations between variables were evaluated through Spearman's correlation coefficient. The Kaplan–Meier curves and log-rank test were applied to compare survival differences between subgroups. Cox proportional hazards model was used to perform an independent prognostic analysis. Statistical significance was defined as a two-tailed *p* < 0.05.

## 3. Results

**Figure 1:**
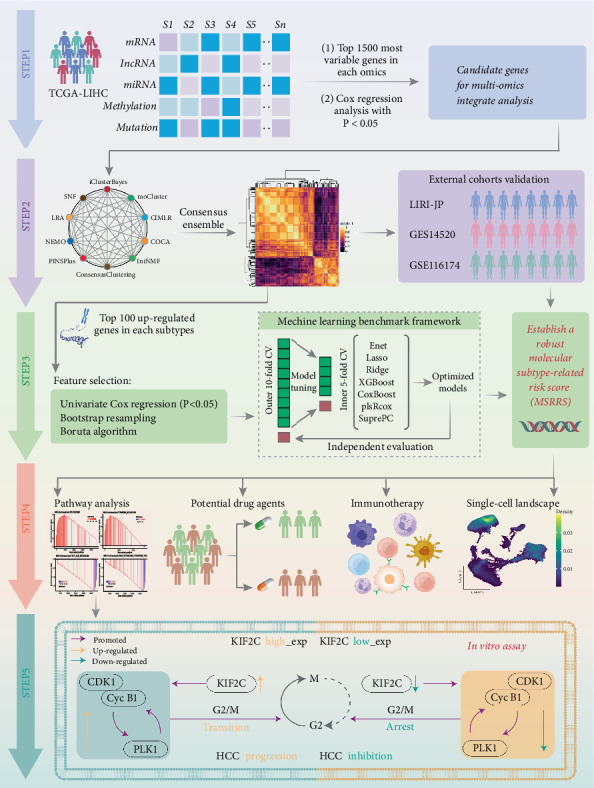
Flowchart of the present study.

The workflow of this research is shown in Figure 1.

### 3.1. Identification of Novel HCC Multiomics MS

**Figure 2:**
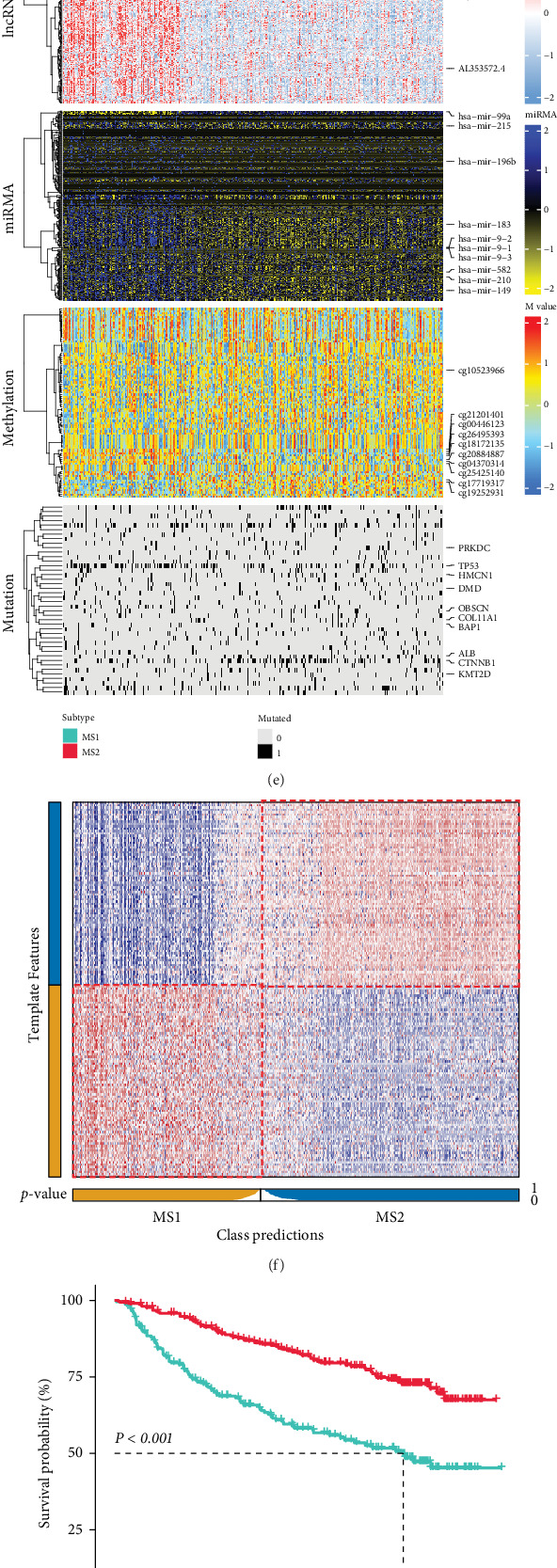
Identification and validation of the multiomics integrative consensus MSs of HCC. (a) The optimal number of multiomics clusters was determined by combining the cluster prediction index, gap statistics, and previous clustering experience with HCC. (b) Clustering of HCC patients through 10 cutting-edge multiomics clustering methods. (c) Consensus clustering matrix for two HCC MSs based on the 10 algorithms. (d) Differences in OS between the two HCC MSs in the TCGA-LIHC cohort. (e) Comprehensive heat map of multiomics integrative consensus MSs. (f) Validation of HCC MSs in the nearest template of the Meta-HCC cohort. (g) Differences in OS between the two HCC MSs in the Meta-HCC cohort. (h) Consistency of MSs with NTP and consistency of MS with PAM in the TCGA-LIHC cohort and consistency of NTP with PAM in the Meta-HCC cohort.

Based on cluster prediction index, gap statistical analysis, silhouette score, and prior research experience, we classified HCC samples from the TCGA-LIHC cohort into two MSs (Figure 2a, Figure S1A, and Table S4). The new molecular clustering with 10 cutting-edge multiomics clustering methods for patients is shown in Figure 2b. Unsurprisingly, there were significant molecular features and prognostic differences between the two MSs (Figure 2c,d). The integrated heat map revealed a distinct distribution of genes and CpG sites exhibiting the greatest degree of variability, along with genes displaying the highest mutation frequency, between the two subtypes. This indicates that such clustering can unveil the heterogeneity of HCC across multiomics dimensions (Figure 2e). Before performing external validation, we first combined three independent HCC cohorts into a Meta-HCC cohort. The results of PCA confirmed that the batch effect was effectively removed (Figure S1B,C). Based on 200 MS-related marker genes (Table S5), we then deployed the 200-gene classifier to the Meta-HCC cohort using the NTP algorithm (Figure 2f). As expected, prognostic differences between the two MSs remained consistent with the TCGA-LIHC cohort (Figure 2g). And the predictions made by NTP and PAM algorithms show a high level of consistency with the actual MSs in the TCGA-LIHC cohort. Additionally, the predictions made by both algorithms demonstrate a strong level of agreement in the Meta-HCC as well (Figure 2h). Finally, the results of DO terms suggested that the 200 marker genes were strongly associated with cancer (Figure S1D), again demonstrating the robustness of the MSs.

### 3.2. Differential Molecular Landscape Across HCC Subtypes

Initial analyses revealed marked variations in hallmark gene set activation profiles across the two MSs (Figure S2A). In detail, MS2 with a better prognosis has more active metabolic activity, whereas MS1 with a worse prognosis is characterized by activation of the cell cycle and inflammatory response, as well as high proliferative activity. Subsequently, we comprehensively analyzed the enrichment of KEGG metabolic pathways between the two MSs. As shown in Figure S2B, there were indeed significantly different metabolic patterns between the two MSs. Precisely, the metabolism of carbohydrate, lipid, amino acid, cofactor, and vitamin, as well as xenobiotics, is more active in MS2, whereas high enrichment of glycan metabolism and nucleotide metabolism may be one of the drivers of the malignant behavior of MS1. Then, to further investigate transcriptomic differences, we analyzed the expression of 23 TFs and potential regulators associated with cancer chromatin remodeling. Unsurprisingly, the expression patterns of the two MSs were significantly different (Figure S2C), which may be another important driver of HCC heterogeneity. Finally, we assessed the association of molecular clustering with TME. Figure S2D demonstrates marked disparities in immune checkpoint gene expression profiles and differential activation states of immune pathways between the two MSs, indicating substantial differences in the TME. For instance, MS1 displayed heightened suppression of antitumor immunity through immune checkpoints as well as enhanced antitumor immune responses via cytolytic activity and T-cell activation. In contrast, MS2 exhibited characteristics more akin to immune desert phenotypes, lacking diverse immune activities. This was further substantiated by the abundance levels of various immune cell types estimated using six independent algorithms, showing predominantly low abundances of immune cell types in MS2 (Figure S2E). Thus, the new molecular clustering demonstrates the ability to differentiate TME which may open up opportunities for personalized immunotherapy strategies for HCC patients.

### 3.3. Development of the MSRRS

**Figure 3:**
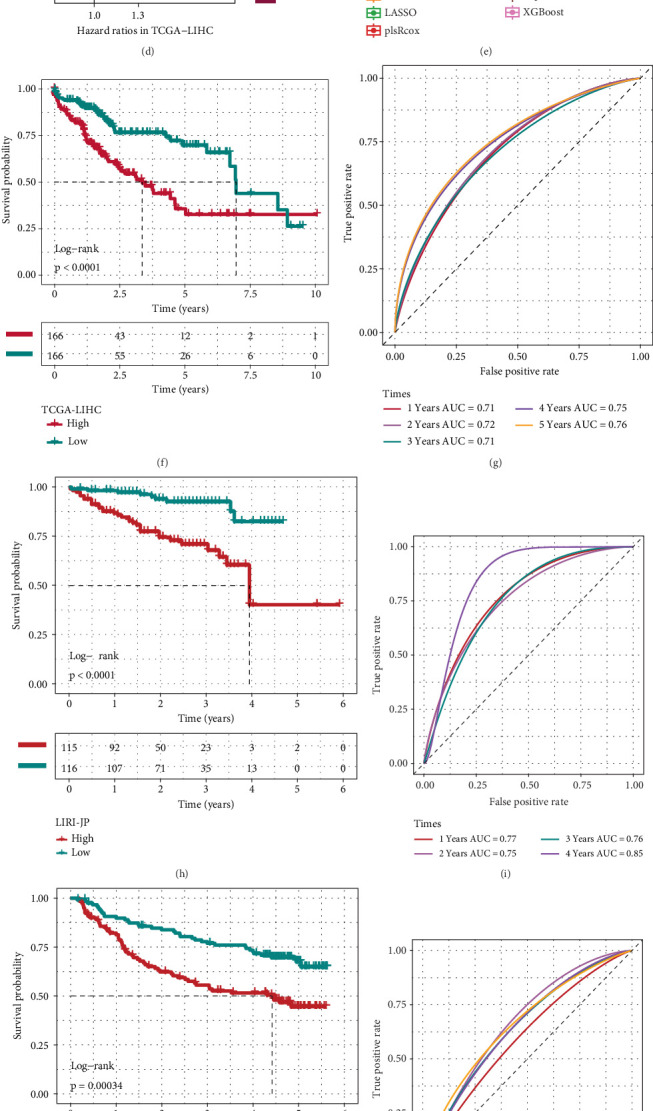
Development and validation of MSRRS. (a) PCA of training and validation cohorts after batch correction. (b) Results of univariate Cox regression analysis of 200 MS-associated markers. (c) Feature gene selection based on the Boruta algorithm. The box plot shows the importance of each gene during model calculation. The green boxes represent important genes. (d) Detailed hazard ratios for 10 MSRRS genes. (e) Comparison of C-index and IBS of seven survival-related machine learning algorithms by nest cross-validation. (f) Differences in OS between high and low MSRRS risk groups in the TCGA-LIHC cohort. (g) Time-dependent ROC curves for MSRRS to predict OS in the TCGA-LIHC cohort. (h–o) Validation of the MSRRS in multiple external HCC cohorts.

First, we removed the batch effect between individual HCC cohorts, and the results of PCA before and after removing the batch effect are displayed in Figure S3A and Figure 3a. Univariate Cox regression analysis of 200 MS-associated markers identified 93 prognostically relevant genes (Table S6), comprising 50 protective genes and 43 risk genes (Figure 3b). Detailed hazard ratios for prognostic genes are shown in the forest plot (Figure S3B).

Subsequently, bootstrapping validation identified 38 resampling-stable candidates from the initial 93 genes (Table S7 and Figure S3C). Then, the number of selected genes was subsequently reduced using the Boruta algorithm (Figure S3D), leading to the identification of 10 genes that were highly correlated with OS (Table S8 and Figure 3c,d). Finally, utilizing the 10 Boruta-selected genes, we conducted a comparative evaluation of seven machine learning algorithms, aiming to identify the hyperparameter-optimized model demonstrating optimal generalizability with minimal overfitting risk. According to the results presented in Figure 3e and Table S9, the Ridge survival model demonstrated superior predictive accuracy, attaining both the maximum mean C-index and minimal mean IBS among all evaluated methods. Therefore, we applied a hyperparameter-tuned Ridge model to the TCGA-LIHC cohort, designating this approach as MSRRS. The details of the hyperparameter tuning of the Ridge model (e.g., the regularization factor *λ*) are shown in Table S1. The Kaplan–Meier analysis further demonstrated significantly reduced OS in high MSRRS patients versus their low-score counterparts (Figure 3f). The time-dependent ROC analysis demonstrated acceptable predictive performance of MSRRS across 5 years in the TCGA-LIHC cohort, with AUC values progressively increasing from 0.71 (1 year) to 0.76 (5 years), peaking at 0.75 and 0.76 in the final 2 years (Figure 3g). This consistency in discriminative ability highlights MSRRS as an effective prognostic indicator for HCC patients. Further correlation analysis suggested that MSRRS was significantly associated with clinical stage and tumor grade (Figure S3E). And subsequent Cox analysis suggested that MSRRS could be independent of age, stage, and grade as an independent prognostic factor for OS of HCC patients (Figure S3F,G). Finally, to assess the generalizability of MSRRS, we also evaluated its performance in predicting OS across multiple independent cohorts. The results demonstrated that MSRRS accurately predicted patients' OS in the LIRI-JP cohort (Figure 3h,i), GSE14520 cohort (Figure 3j,k), GSE116174 cohort (Figure 3l,m), and meta cohort (Figure 3n,o). The collective evidence suggests that the MSRRS holds clinical potential for predicting HCC patient outcomes.

### 3.4. Pathways Associated With MSRRS

**Figure 4:**
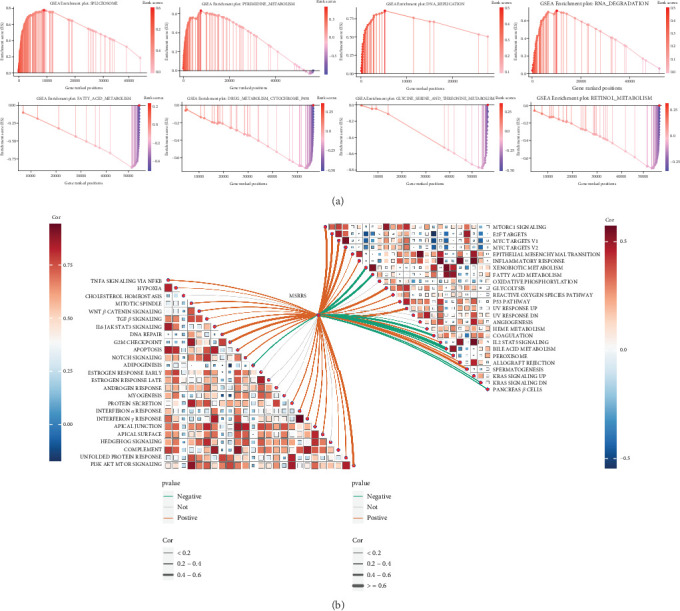
Identification of pathways associated with MSRRS. (a) The Top 4 KEGG pathways identified by GSEA that were enriched in the high MSRRS group and low MSRRS group. (b) Correlation of MSRRS with the GSVA scores for hallmark gene sets.

The GSEA showed that MSRRS exhibited significant positive correlations with multiple oncogenic pathways, notably involving cell cycle progression, spliceosome machinery, and DNA replication processes, whereas many metabolism-related pathways were negatively associated with MSRRS (Table S10). The Top 4 pathways significantly enriched in high MSRRS group and low MSRRS group are shown in Figure 4a. Subsequently, we assessed the correlation between MSRRS and the activity of 50 hallmark gene sets as quantified by GSVA, leading to conclusions consistent with those above (Figure 4b). For example, the activity of the G2M checkpoint and E2F targets was significantly positively correlated with MSRRS, which is consistent with the finding that the cell cycle is enriched in the high MSRRS group. Meanwhile, MSRRS is also associated with multiple immune response pathways, which suggests that it may have the potential to predict the components of TME. Finally, we obtained the genes with significant interactions with the above 10 genes through the GeneMANIA database. As shown in Figure S4, the network of interactions is complex, but the vast majority of gene functions involve cell cycle regulation, which reaffirms the link between MSRRS and cell cycle.

### 3.5. Screening of Potential Therapeutic Targets and Drugs

**Figure 5:**
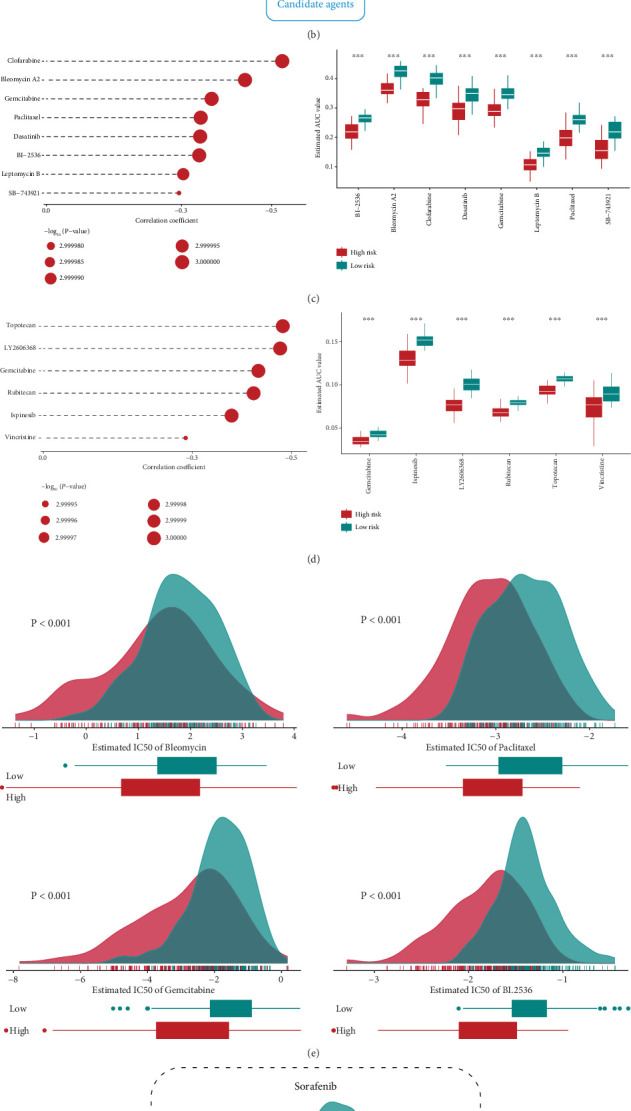
Screening for potential therapeutic targets and drugs associated with MSRRS. (a) Potential therapeutic targets were derived by taking the intersection of proteins significantly positively correlated with MSRRS and genes whose CERES scores were significantly negatively correlated with MSRRS. (b) Comprehensive computational pipeline for discovering potential therapeutic agents for high MSRRS patients. Potential therapeutic agents acquired from (c) CTRP and (d) PRISM databases. (e) Validation of sensitivity of high and low-risk groups to potential therapeutic agents based on the IC_50_ estimated by GDSC database. Difference in IC_50_ of (f) sorafenib and (g) several classical anticancer drugs between high- and low-risk groups.

To identify potential therapeutic targets associated with MSRRS, we first identified druggable targets whose RNA expression was significantly positively correlated with MSRRS (*r* > 0.4). Then, we found genes in which CERES was significantly negatively correlated with MSRRS (*r* < −0.4). As a result, a total of 11 druggable targets (AKR1B1, FKBP1A, CTSC, ACTR3, POLD1, GAPDH, TUBA1B, ACTB, ACAN, TACC3, and ATP6V1C1) fulfilled both of these conditions (Figure 5a). Notably, in most HCC cell lines, CTSC exhibited a CERES score above the nonessential threshold, suggesting limited dependency on this protease in HCC pathogenesis. Consequently, the investigation identified 10 other genes demonstrating higher therapeutic potential for targeted intervention (Figure S5A,B). Subsequently, we uncovered therapeutic agents whose drug sensitivity was associated with MSRRS based on the CTRP and PRISM databases. Candidate drugs were selected based on two criteria: (1) exhibiting reduced AUC (log2FC > 0.2) within the high MSRRS cohort and (2) demonstrating a significant inverse correlation between their AUC values and MSRRS scores (*r* < −0.25). Statistical significance was defined as a two-tailed *p* < 0.05. The pipeline for the screen of potential agents is shown in Figure 5b. The above analyses yielded eight CTRP-derived compounds (clofarabine, bleomycin A2, gemcitabine, paclitaxe, dasatinib, BI-2536, leptomycin B, and SB-743921) and six PRISM-derived compounds (gemcitabine, ispinesib, LY2606368, rubitecan, topotecan, and vincristine) that were screened as potential therapeutic agents (Figure 5c,d). In order to explore the sensitivity of the drugs from multiple perspectives, we conducted comparative analysis of drug response disparities, evaluating IC_50_ variations between high and low MSRRS subgroups using GDSC database. As expected, we obtained results consistent with the aforementioned (Figure 5e). Given that these drugs are not currently first-line treatment options for HCC, we further compared the IC_50_ of the first-line treatment drug sorafenib in the two MS subgroups. The analysis demonstrated that elevated MSRRS levels correlated with enhanced therapeutic responsiveness to sorafenib (Figure 5f). In addition, we identified more classical anticancer drugs whose sensitivity correlates with MSRRS. For example, patients exhibiting reduced MSRRS levels could potentially demonstrate improved therapeutic responses to AKT Inhibitor VIII administration, whereas drugs such as cisplatin, docetaxel, doxorubicin, rapamycin, and JNK Inhibitor VIII may be more effective in patients with high MSRRS (Figure 5g).

### 3.6. MSRRS Has the Ability to Predict ICI Treatment Response

**Figure 6:**
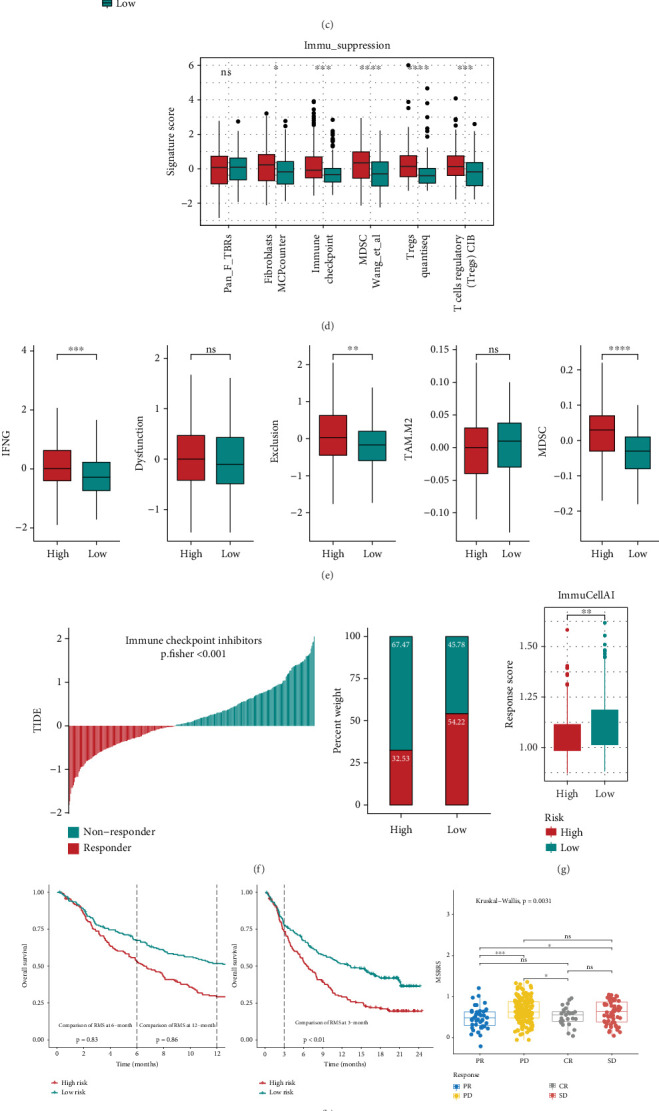
Association between MSRRS and TME. (a) Differences in MSRRS between samples of different immune subtypes. Differences in abundance of (b) different immune cell types, (c) activity of anticancer immune cycle, and (d) enrichment scores of immunosuppressive markers between high- and low-risk groups. (e) Differences in levels of five signatures predicted by the TIDE algorithm between high- and low-risk groups. Differences in (f) TIDE scores and (g) ImmuCellAI response scores between high- and low-risk groups. Validating the correlation between MSRRS and ICI treatment response in (h) IMvigor210, (i) GSE78220, (j) GSE135222, and (k) GSE91061 cohorts.

To characterize TME variations across MSRRS-defined subgroups, we comprehensively assessed the genomic profiles of various immunomodulators and found significantly different multiomics patterns between the two groups (Figure S6A). Also, we note that there is a higher MSRRS in the C1 and C2 immune subtypes, which are characterized by high heterogeneity and high proliferative activity, which is consistent with our labelling of MSRRS annotations (Figure 6a).

It is well known that the components of TME are critical to the response to ICI therapy. Therefore, we first assessed the differences in enrichment of TME cell type signatures between two MSRRS subgroups. As illustrated in Figure 6b, notable variations in immune cell composition were observed between the two cohorts. Particularly, patients with high MSRRS demonstrated markedly elevated MDSC levels compared to their low MSRRS counterparts. These immunosuppressive cells have been widely recognized as key contributors to tumor-associated immunosuppression through multiple biological mechanisms. Subsequently, we assessed differences in the activity of a series of stepwise events associated with the cancer-immunity cycle between high and low MSRRS groups. The results showed that the samples in the high MSRRS group had higher enrichment scores at the stage of releasing cancer cell antigens, suggesting that these samples had a high degree of heterogeneity and more release of cancer antigens. Meanwhile, there was also a significant difference in the trafficking of multiple immune cell types to tumors between the high- and low-risk groups. And this contains both Treg and MDSC, both of which are capable of mediating the suppression of anticancer immunity and are characteristic of TME in the high MSRRS group (Figure 6c). To prove this hypothesis, we further assessed the enrichment levels of multiple immunosuppression-related signatures. Unsurprisingly, the estimated abundance of Treg and MDSC was indeed higher in the high MSRRS group. And the quanTIseq algorithm and the CIBERSORT algorithm yielded consistent predictions in terms of the abundance of Treg (Figure S6B), which again demonstrates the robustness of the above conclusions. Moreover, our analysis revealed markedly higher immune checkpoint activity in the high MSRRS cohort, indicating that immune evasion mechanisms facilitated by these pathways could be more common in these samples (Figure 6d). Meanwhile, specimens with heightened MSRRS exhibited stronger immune exclusion characteristics (Figure S6C). The above findings suggest that there may be differences in the response to ICI among patients in different risk groups, and this hypothesis was confirmed by subsequent analyses of biomarkers for ICI treatment response (Figure S6D).

At this point, we believe that MSRRS can guide the clinical application of ICI. Thus, we first employed the TIDE algorithm to evaluate specimens' sensitivity to ICI therapy. As anticipated, the high MSRRS group exhibited elevated T-cell exclusion indices, concomitant with increased MDSC infiltration and diminished therapeutic responsiveness (Figure 6e,f). Meanwhile, the results from the ImmuCellAI algorithm also suggest that the high-risk group is less responsive to ICI treatment (Figure 6g). So far, the results of multiple algorithms have confirmed a significant positive correlation between MSRRS and MDSC abundance (Figure S6E), which exposes that MDSC may be a bridge between MSRRS and anticancer immunosuppression. Subsequently, we validated the performance of MSRRS in predicting ICI treatment response in multiple immunotherapy cohorts. In the IMvigor210 cohort, patients with elevated MSRRS levels demonstrated significantly reduced OS following ICI therapy compared to counterparts with lower MSRRS levels. Meanwhile, samples that were effective for ICI treatment had a lower MSRRS than insensitive samples (Figure 6h). These findings were also confirmed in the GSE78220, GSE135222, and GSE91061 cohorts (Figures 6i, 6j, and 6k). Given the established roles of TMB and tumor neoantigen burden (TNB) as predictive biomarkers for immunotherapy response, we investigated their correlation with MSRRS. The results indicate that MSRRS is independent of TMB and TNB (Figure S6F,G).

### 3.7. Single-Cell Transcriptional Patterns Associated With MSRRS

**Figure 7:**
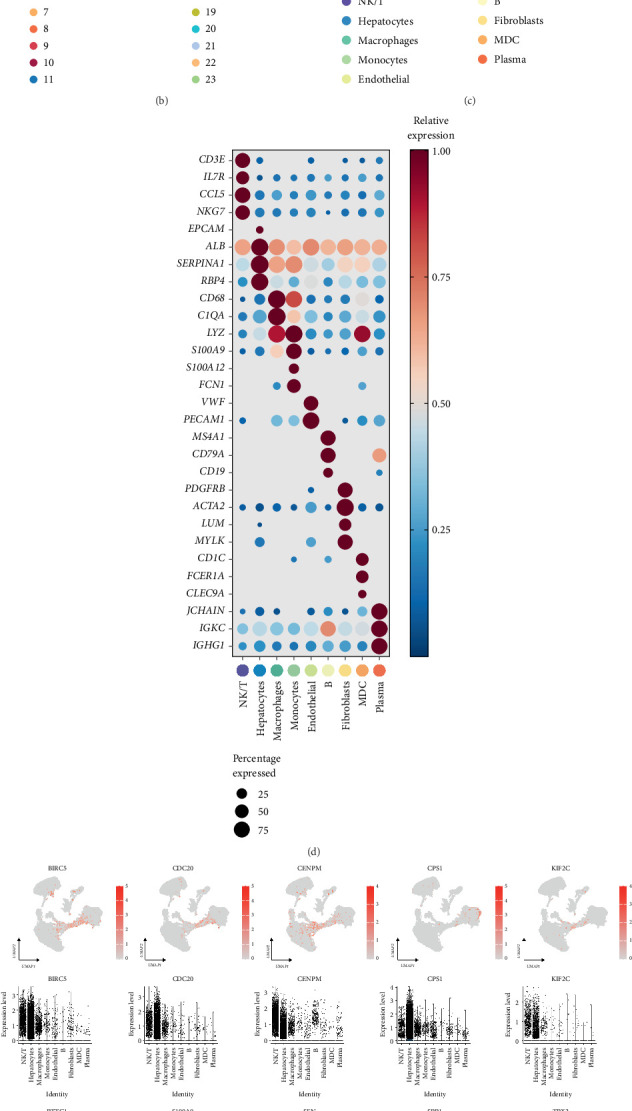
Single-cell transcriptional patterns associated with MSRRS. UMAP analysis of the distribution of (a) 21 HCC samples, (b) 24 cell clusters, and (c) 9 main cell types. (d) Potential marker genes for annotation of nine main cell types. (e) UMAP analysis and (f) dot plot of the distribution of MSRRS genes expression levels in different cell types. (g) UMAP analysis of the distribution of MSRRS gene expression density in different cell types.

After a data quality control process, we obtained 72,491 cells from a total of 16 tumor samples from two independent HCC cohorts and successfully removed the batch effect between samples (Figure 7a). First, we performed dimensionality reduction using UMAP, followed by clustering analysis that identified 24 cell clusters (Figure 7b). The potential marker genes for each cluster are shown in Figure S7. Subsequently, we identified nine predominant cell types by analyzing 29 marker genes (Figure 7c). Marker gene expression profiles across distinct cell types are presented in Figure 7d. To investigate MSRRS's functional role with single-cell resolution, we systematically profiled 10-gene expression patterns across distinct cellular subtypes. The results indicate that each gene is expressed relatively specifically in certain cell types. For example, most genes are expressed at high levels in cancer cells, which confirms their involvement in the progression of HCC. Meanwhile, S100A9 and SPP1 were also specifically expressed in monocytes and macrophages, suggesting that both may be associated with myeloid cell–mediated immunosuppression (Figure 7e,f). And the conclusions of subsequent studies on the expression density of genes in different cell types are consistent with the above findings (Figure 7g), which again confirm the link between MSRRS and the HCC microenvironment.

### 3.8. KIF2C Promotes G2/M Transition in HCC Cells by Targeting CDK1/CCNB1/PLK1 Signaling

To further explore the link between MSRRS and the biological behavior of HCC, we proposed to screen a relatively critical gene and confirm its potential mechanism of regulating HCC cell growth by in vitro experiments. First, we conducted a comprehensive literature review focusing on 10 MSRRS genes. By screening nearly 1000 publications, we systematically summarized the biological functions of each MSRRS gene in HCC (Table S11). Through this comprehensive review, we confirmed that each MSRRS gene indeed plays a critical role in HCC pathogenesis. Notably, numerous studies have examined the roles of SPP1 and BIRC5 in HCC, so their roles in HCC have been initially clarified. However, studies on certain genes in HCC, such as KIF2C, CENPM, and SFN, remain limited. Therefore, we aim to conduct follow-up experiments targeting this category of genes. Subsequently, we analyzed the differences in MSRRS genes expression between HCC and noncancer tissues. In HCC tissues, except for CPS1 and S100A9 expression, which were significantly downregulated, the other eight genes were significantly upregulated (Figure S8A). Interestingly, S100A9, whose expression is downregulated in HCC, was found to be associated with poor OS in our aforementioned survival analysis. And in paired cancerous and noncancerous tissues, we came to the same conclusion (Figure S8B). Consequently, we obtained the CPTAC HCC proteomic cohort from the UALCAN database (https://ualcan.path.uab.edu/) to re-evaluate the expression patterns of MSRRS genes at the protein level. Since the proteins encoded by BIRC5 and PTTG1 were excluded from the quality-controlled proteomic expression matrix, we ultimately analyzed the remaining eight MSRRS proteins. As shown in Figure S8C, the differential expression patterns of these proteins between HCC and normal tissues were entirely consistent with their transcriptional-level findings, and all proteins showed upregulation in HCC tissues except S100A9 and CPS1 which showed downregulation in HCC. Notably, both at the mRNA and protein levels, KIF2C and SFN exhibited markedly significant expression differences between HCC and normal tissues. Subsequent correlation analysis between gene expression profiles and MSRRS revealed CDC20, KIF2C, and SPP1 as genes with marked associations (*r* > 0.7, *p* < 0.05) (Figure S8D). Combining the above factors and the hazard ratios for each gene, we finally identified KIF2C as a target molecule for subsequent in vitro studies.

**Figure 8:**
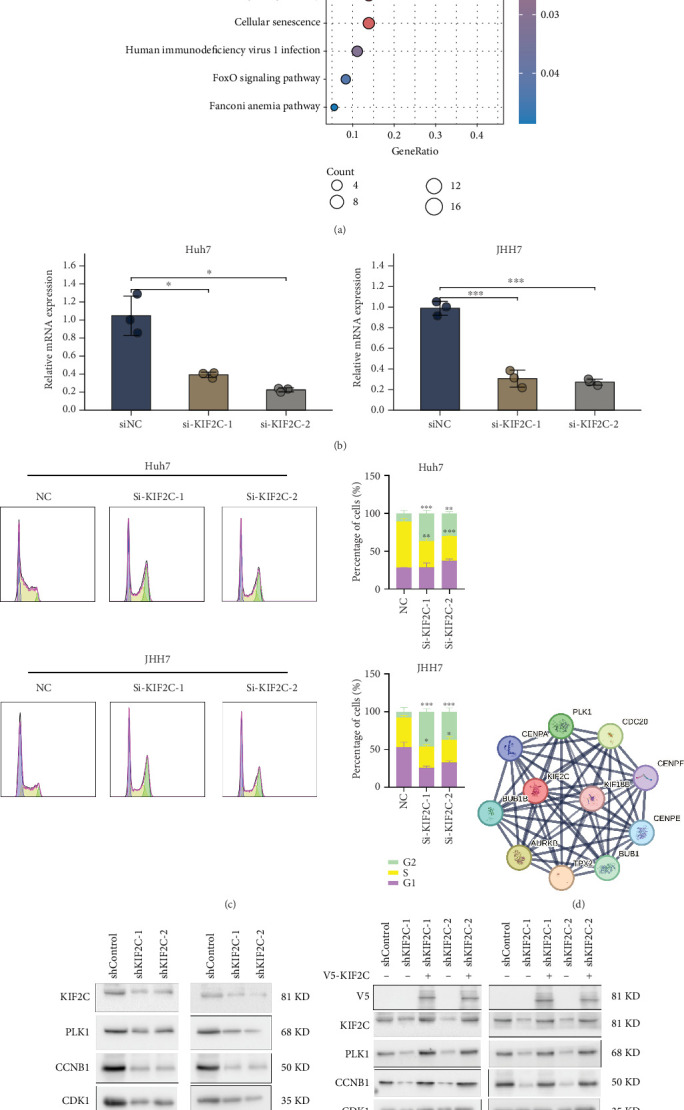
KIF2C promotes G2/M transition in HCC cells by targeting CDK1/CCNB1/PLK1 signaling. (a) Pathway enrichment analysis of KIF2C-related genes in HCC. (b) Real-time quantitative PCR confirmed the reduction in KIF2C mRNA after siRNA knockdown. (c) Flow cytometry assay confirmed that KIF2C knockdown primarily induced cell cycle arrest in G2/M phase. (d) PPI network consisting of proteins interacting with KIF2C. (e) Western blot experiments confirmed the reduced expression of CDK1, CCNB1, and PLK1 in KIF2C knockdown HCC cells at the protein level. (f) Rescue experiments demonstrated that reintroducing V5-tagged KIF2C into depleted cells effectively normalized CDK1, CCNB1, and PLK1 expression to baseline levels.

To investigate the primary role of KIF2C in HCC, we performed pathway enrichment analysis using genes significantly correlated with KIF2C (|*r*| ≥ 0.8, *p* < 0.05) based on the TCGA-LIHC cohort (Table S12). The findings indicate that the cell cycle may be the main pathway by which KIF2C regulates HCC progression (Figure 8a and Table S13). Then, we interrogated the CCLE resource to quantify KIF2C expression in distinct liver CCLs (Figure S8E). By evaluating the KIF2C expression profiles across available cell lines in our laboratory, we identified Huh7 and JHH7 as optimal models for further investigation due to their robust KIF2C expression levels. After knockdown of KIF2C expression (Figure 8b), we explored its effects on the cell cycle of Huh7 cells and JHH7 cells. The results indicated that KIF2C knockdown mainly induced cell cycle arrest in the G2/M phase (Figure 8c). KEGG graph also demonstrates KIF2C's predominant involvement in cell cycle control during G2/M phase, while CDK1/CCNB1/PLK1 signaling may be the most critical pathway (Figure S8F). The results of the PPI network were also consistent with the aforementioned, suggesting that KIF2C has a significant reciprocal relationship with PLK1 (Figure 8d). Western blot experiments confirmed the reduced expression of CDK1, CCNB1, and PLK1 in KIF2C knockdown cancer cells at the protein level (Figure 8e). Subsequent rescue experiments demonstrated that reintroducing V5-tagged KIF2C into depleted cells effectively normalized CDK1, CCNB1, and PLK1 expression to baseline levels, confirming these molecular changes directly result from KIF2C depletion (Figure 8f). In summary, we can conclude that KIF2C promotes G2/M transition in HCC cells through CDK1/CCNB1/PLK1 pathway.

## 4. Discussion

Revealing the heterogeneity of cancers and establishing molecular classifications are essential for achieving precise and individualized cancer treatment. This approach has been successfully demonstrated in various cancer types, with breast cancer serving as one of the most prominent examples [37]. Although HCC is one of the few cancer types with a relatively clear etiology, its heterogeneity has not been thoroughly investigated to date, which has hindered the advancement of personalized treatment for HCC. In recent years, advances in systemic therapies, including TKIs and ICIs, have revolutionized the treatment paradigm for advanced stage or unresectable HCC. However, the limited treatment response rate has resulted in the overall prognosis of HCC patients remaining unsatisfactory. Currently, the MSs of HCC have been initially explored, and it is widely recognized that HCC can be divided into proliferation class and nonproliferation class. While this classification thoroughly assesses the numerous molecular features of HCC, the current treatment options for HCC remain largely determined by clinical stage, which suggests that current HCC classifications are still insufficient in guiding personalized treatment options.

Throughout all phases of tumorigenesis, oncogene activity is precisely modulated through distinct molecular mechanisms, including genetic alterations such as mutations and epigenetic regulation involving DNA methylation or histone remodeling [38]. Most previous studies have categorized HCC samples only at the transcriptomic level, making it difficult to accurately explore HCC heterogeneity [39]. Here, we employ an integrative multiomics approach to comprehensively characterize the genomic heterogeneity landscape in HCC. We also discarded the traditional clustering algorithms and instead integrated the latest 10 clustering algorithms and successfully demonstrated a novel molecular classification method for HCC. The two HCC MSs we identified differed significantly in prognostic and pan-genomic landscapes and had stable clustering results across multiple HCC cohorts, both affirming our successful exploration of HCC heterogeneity. A few of these findings are similar to our previous findings, such as the assessment of the immune microenvironment and the activity of various metabolic pathways [40]. But there are still more unanswered questions to be explored in the future.

So what are the similarities and differences between the multiomics MSs we identified and the traditional molecular classifications of HCC? Before unraveling this mystery, we must specially emphasize that due to the high heterogeneity of HCC, no two HCC samples share identical genomic profiles. Therefore, all current molecular classifications can only categorize samples based on relatively broad characteristic features. In the traditional molecular classification of HCC, the proliferation and nonproliferation subclasses each account for approximately 50% of all cases. The distinction between these two molecular subclasses encompasses nearly all aspects of molecular biology, including pathological features (e.g., IHC markers and cell differentiation), etiological factors (e.g., HBV infection and alcohol consumption), genomic characteristics, immunological profiles, and clinical indicators [11]. However, these molecular characteristics are derived from aggregated findings of numerous previous studies, which may not guarantee consistency in study populations or comprehensiveness in research scope. For instance, while HBV infection and TP53 mutations are currently recognized as key features of proliferative HCC, this association is not strictly correlated, as these two factors do not absolutely co-occur in clinical practice. Relying on findings from a previous study [41], we successfully matched cases from the TCGA-LIHC cohort with proliferation/nonproliferation subclasses and compared our identified multiomics MSs, MSRRS subgroups, and traditional HCC classifications (Figure S9). Although the exclusion of some cases during analysis led to a proportional imbalance between high-/low-risk groups or proliferation/nonproliferation subclasses, this did not affect the final conclusions. The results show that our molecular classification correlates significantly with the traditional classification of HCC but also exhibits some differences. For example, the proliferation class HCC is significantly reassigned by our clustering. The main reason for this discrepancy may be that our clustering focuses on deeper clustering at the genomic level only. However, this does not indicate a bias in our findings, as almost all differences at the biological level are embedded in genomic features; this suggests precisely that our clustering may be better suited to dissecting the heterogeneity of HCC. In addition, the high concordance between MSs and risk subgroups again demonstrates the robustness of our clustering. Nevertheless, thoroughly dissecting the heterogeneity of HCC remains a formidable challenge. Only through closely integrating our discoveries with existing findings can we make substantive progress in unraveling the complexity of HCC heterogeneity.

Next we explore the clinical implications of our findings. Considering that machine learning has emerged as a powerful tool for multiomics data analysis, with growing applications in clinical survival prediction [28, 42, 43], we successfully constructed MSRRS based on the characterization of two HCC MSs by machine learning algorithms. The MSRRS demonstrated robust prognostic prediction capabilities for HCC patients, exhibiting significantly higher accuracy compared to conventional prediction models in clinical validation studies [39, 40, 44]. This highlights the value of MSRRS in stratifying patient prognosis. In recent years, many studies have incorporated prognostic biomarkers and clinical characteristics into nomogram to improve the accuracy of predicting patient prognosis. Therefore, we also attempted to integrate MSRRS with clinical stage into a nomogram based on the results of multivariate Cox analysis (Figure S10). Although the calibration curves suggest that the nomogram is highly consistent with the actual OS of patients, the decision curves found that the predictive performance of MSRRS combined with clinical stage did not exceed that of MSRRS alone, which once again proves that MSRRS is effective in predicting patient OS. Notably, our novel MSRRS system could provide actionable insights for optimizing therapeutic regimens in HCC management. Currently, multikinase inhibitors or antiangiogenic agents combined with ICIs are the first-line therapeutic strategy for unresectable or advanced HCC [4, 11]. Of these, sorafenib was for a long time the preferred therapeutic regimen for HCC since its approval, although in recent years it has been gradually replaced by other TKIs such as lenvatinib [11, 45]. Our analysis revealed a positive correlation between elevated MSRRS and improved pharmacological response to sorafenib treatment. A previous study has found that in HCC cells treated with sorafenib, phospho-STAT3 is inhibited, thereby reducing the expression level of survivin [46]. Survivin, encoded by the BIRC5 gene (one of the MSRRS-related genes), shows an association between its expression and sorafenib sensitivity, which partially supports the potential of MSRRS as a predictive biomarker for sorafenib therapeutic efficacy. Unfortunately, we were unable to assess the association of MSRRS with the sensitivity of several other TKIs approved for the treatment of HCC, at this time, which will need to be further evaluated in the future. The introduction of ICI has also significantly enhanced prognostic outcomes for intermediate-advanced HCC patients in recent years, solidifying their position as fundamental components in contemporary HCC systemic therapy [47]. We found that MSRRS was strongly associated with TME in HCC samples, not only in terms of abundance of immune cells and activity of immune function but also in terms of response to ICI treatment. Specifically, cases in the low-risk group had a higher response rate to ICI and a more pronounced survival benefit. High-risk samples had higher levels of Treg and MDSC enrichment, which may be an important reason for their poor response to ICI. A recent mechanistic study revealed that in HCC cells, the tRNA-derived fragment tRF5-GlyGCC interacts with KDM6B to epigenetically enhance Runx2 expression. This regulatory axis subsequently stimulates transcriptional activation of S100A9, which promotes MDSC recruitment, ultimately leading to indirect suppression of NK cell antitumor functions through MDSC-mediated immunosuppression [48]. Meanwhile, inhibition of S100A9 or myeloid-specific knockout of TLR4/RAGE, the receptors of S100A9, impeded ETV5-induced PMN-MDSC recruitment [49]. These findings provide evidence for S100A9-mediated recruitment of MDSCs and partially explain why cases in the high-risk group exhibit poor response to ICI therapy. Meanwhile, SPP1 was found to modulate both MDSCs and Tregs to promote immune evasion [50]. Studies exploring from the perspective of macrophages or monocytes also revealed potential mechanisms of MSRRS in predicting the efficacy of ICI. For instance, elevated levels of SPP1+ macrophages have been found to be associated with poor response to ICI therapy [51, 52]. S100A9 + CD14 + monocytes were also found to contribute to resistance to ICI in HCC by attenuating T-cell–mediated antitumor function [53]. The above findings coincide precisely with our conclusions. However, our results are based solely on computational algorithm estimates. Future studies should involve recruiting independent HCC immunotherapy cohorts and validating these predictions through experimental techniques such as flow cytometry or multiplex immunohistochemistry. In addition, we also identified several potential targets that are significantly correlated with MSRRS and have the potential to be used in the treatment of HCC. Genes such as ACTB [54], TUBA1B [55], POLD1 [56], TACC3 [57, 58], and AKR1B1 [59], for example, have all been studied for their association with HCC. Although these targets are not currently in the biological testing phase, our findings will undoubtedly provide more opportunities for future targeted therapies for HCC. Currently, chemotherapy has been used less and less in systemic treatment of HCC, but its use in transcatheter arterial chemoembolization (TACE) and hepatic artery infusion chemotherapy (HAIC) is still necessary [60, 61]. Our analysis indicates that patients in different risk subgroups exhibit significant differences in their responsiveness to several standard chemotherapeutic agents. For instance, patients in the high-risk group demonstrate heightened sensitivity to drugs such as vincristine and paclitaxel. These findings have been partially validated by both in vivo and in vitro studies. Taking BIRC5 as an example, its expression level may correlate with the sensitivity of HCC cells to vincristine [62] and paclitaxel [63], which aligns with our hypothesized conclusions. This evidence suggests that our proposed risk stratification is an important tool for optimizing personalized treatment strategies.

Another central theme is how to explain the HCC heterogeneity we have tapped into at the molecular level. We found significant differences in cellular processes, genetic information processing, and metabolic activity between samples from two subgroups, which can serve as a hallmark feature of HCC heterogeneity. This finding has actually been demonstrated in our previous studies and is now further confirmed in a multiomics perspective [39, 40]. The cell cycle is one of the central cellular processes that regulates cell growth and death [64]. We found that the activity of G2M checkpoint, a crucial set of genes that determines whether a cell can enter M phase properly [65], varied dramatically across risk groups. This further focuses our vision on the link between G2M phase transition and HCC heterogeneity.

CDK1 is known as the “commander-in-chief” of the G2/M phase transition and mitosis. The cyclin B family has three members, Cyclin B1, Cyclin B2, and Cyclin B3, the first two of which are able to bind to CDK1 during mitosis, thus activating the kinase activity of CDK1 [66]. Currently, it has been demonstrated that the CDK1/CCNB1 axis can promote HCC progression [67]. PLK1, a member of the polo-like kinase family, is activated by the CDK1/CCNB1 complex, which acts as a downstream target and forms a positive feedback loop [68]. In the present study, we innovatively found that KIF2C was able to promote G2/M transformation in HCC cells through CDK1/CCNB1/PLK1 pathway. A comprehensive review of studies on the role of KIF2C in HCC reveals that current research in this field remains at a nascent stage (Table 1). Over the years, despite the well-established findings of its upregulated expression and association with poor prognosis, research regarding its mechanistic involvement in HCC progression has remained confined to demonstrating its capacity to regulate the cell cycle and thereby promote the proliferation of HCC cells in vitro [69]. It was only in recent years that studies have found that the putative oncogene KIF2C exhibits multifaceted oncogenic properties in HCC, significantly enhancing malignant phenotypes including tumor cell proliferation, migratory capacity, and invasive potential, ultimately driving metastatic progression. Potential pathways may include the Ras/MAPK signaling and the MEK/ERK signaling [70, 71]. Meanwhile, as a transcriptional target of Wnt/*β*-catenin signaling, KIF2C serves as a critical molecular bridge connecting this pathway with mTORC1 activation mechanisms, with significant implications for HCC pathogenesis [72]. More importantly, a recent study has shown that silencing KIF2C inhibits HCC progression and maintains the cell cycle in the G2 phase [73]. This conclusion is strikingly consistent with our research findings, further validating that KIF2C serves as a critical regulator of the G2/M phase transition during the HCC cell cycle. The liver serves as the most vital metabolic organ in the human body, endowing HCC with distinct metabolic characteristics and a unique tumor immune microenvironment. To investigate whether KIF2C exerts specific oncogenic effects in HCC, we systematically evaluated the expression patterns of KIF2C in pan-cancer cohorts. Notably, the mRNA and protein levels of KIF2C are upregulated in many cancer types (Figure S11A,B), and its mRNA levels correlate with the prognosis of patients with multiple cancer types (Figure S11C), demonstrating its oncogene properties. Next, let us select two solid tumors with higher incidence rates as examples. In the context of lung cancer, although no studies have directly confirmed a direct interaction between KIF2C and CDK1/CCNB1, research has identified a significant positive correlation in their expression levels [74–76]. This suggests that KIF2C in lung cancer may exert its oncogenic effects and influence patient prognosis through pathways similar to those observed in HCC. In breast cancer, there is also evidence that the CDK1/CCNB1/PLK1 axis may be one of the potential pathways by which KIF2C affects tumor progression [77, 78]. However, the effect of KIF2C on OS in breast cancer patients was not significant. Although some studies have demonstrated that KIF2C can affect the prognosis of breast cancer patients [79, 80], we believe that this difference is more due to the stratification of the population and the choice of statistical methods. Thus, the oncogenic role played by KIF2C in breast cancer may not be the dominant mechanism of tumor progression. Nevertheless, we still maintain that KIF2C may exert oncogenic effects through analogous mechanisms across multiple cancer types, which endows it with potential as a pan-cancer therapeutic target.

Although the bootstrap–Boruta integrated algorithm reduces the risk of overfitting, our correlation analysis in Figure S8D revealed potential synergistic interactions within biological pathways among MSRRS genes. For instance, the expression levels of TPX2, CENPM, PTTG1, BIRC5, CDC20, and KIF2C showed significant positive correlations, suggesting that they may participate in a shared molecular interaction network regulating HCC progression, possibly even exhibiting cooperative effects. To further elucidate the heterogeneity of HCC mediated by MSRRS genes, we systematically reviewed the previously established roles of each MSRRS gene in HCC (Table S11). Through this integration of the literature, we identified substantial evidence supporting our hypotheses. For example, studies have revealed that knockout of either TPX2 or CDC20 can induce G2/M phase arrest in HCC cells [81–83]. Furthermore, a previous study demonstrated that in HCC, PLK1 depletion not only induced G2/M phase arrest but also resulted in downregulation of survivin expression, which suggests that BIRC5 may be downstream of PLK1 [84]. The above findings confirm that multiple MSRRS genes may work together to promote the G2M phase transition in HCC and further refine the potential targets of the CDK1/CCNB1/PLK1 axis. Meanwhile, we also found evidence of multiple MSRRS genes interacting with the PI3K/AKT pathway [70, 85–89], suggesting that PI3K/AKT signaling may be an important bridge for MSRRS genes to promote HCC progression and mediate HCC heterogeneity. Overall, there must exist an unknown and intricate interaction network among MSRRS genes, and unraveling this complex network step by step will remain a key challenge in effectively elucidating the heterogeneity of HCC.

In this study, a robust predictive model was successfully established from a multiomics perspective by integrating 10 clustering algorithms and applying a machine learning framework. This model has the potential to accurately predict patient prognosis as well as guide clinical anticancer drug selection. The in-depth analysis of the MSRRS genes has also biologically explained the novel molecular mechanisms driving HCC heterogeneity. Nonetheless, we acknowledge that our study still has some limitations. For instance, the cohort we included lacks independent cohorts from our center, so the clinical generalizability of the foundings requires further validation. In the future, we plan to validate the significance of MSRRS in clinical prognostic stratification and personalized treatment through internal cohort studies and to investigate the underlying mechanisms by which it influences the efficacy of ICI, chemotherapy, or targeted therapy via both in vivo and in vitro experiments. Additionally, the precise molecular pathways through which the MSRRS gene contributes to the carcinogenic process require further in-depth mechanistic investigation, which will constitute another key focus of future in vivo and in vitro studies. Furthermore, since this study primarily investigates transcriptional regulation, proteomic and phosphoproteomic analyses should be supplemented to validate the heterogeneity patterns at the posttranslational level. We also intend to refine this part of the research in future studies.

## 5. Conclusion

Through multiomics integrative clustering analysis, we successfully stratified HCC into two distinct MSs demonstrating divergent clinical outcomes and heterogeneous genomic profiles. Subsequently, a robust MSRRS was developed through a machine learning algorithm framework that has the potential to predict patient prognosis, estimate drug sensitivity, and assess the TME. Finally, in vitro experiments confirmed that the MSRRS gene KIF2C could promote G2/M transition in HCC cells by targeting CDK1/CCNB1/PLK1 signaling. Our findings effectively exposed the heterogeneity of HCC and have the potential to predict prognosis and guide individualized precision therapy.

## Figures and Tables

**Table 1:** Literature review on the role of KIF2C in HCC.

**Year**	**Regulation**	**Prognostic factors**	**Clinical significance**	**Data source**	**Mechanism**	**Research methods**	**PMID**
2017	Upregulated	Poor RFS Poor OS	Poor differentiation Late TNM stage	RNA-seq	—	—	28901309

2020	Upregulated	Poor DFS Poor OS	Poor differentiation Late TNM stage High AFP levels	RNA-seq	KIF2C promotes cell proliferation via regulating the G1-to-S transition of the cell cycle.	In vitro	32509165
	Upregulated	Poor RFS Poor OS	—	RNA-seq Gene microarray	—	—	32775402
	Upregulated	Poor OS	—	RNA-seq Gene microarray	—	—	32391055

2021	Upregulated	Poor DFS Poor OS	Poor differentiation High risk of relapse	RNA-seq IHC	• KIF2C promotes HCC cell proliferation, migration, invasion, and metastasis. • TBC1D7 as a binding partner of KIF2C and this interaction disrupts the formation of the TSC complex, resulting in the enhancement of mTORC1 signal transduction. • KIF2C is a direct target of the Wnt/*β*-catenin pathway and acts as a key factor in mediating the crosstalk between Wnt/*β*-catenin and mTORC1 signaling.	In vivo In vitro	32748349
	Upregulated	Poor OS	—	RNA-seq	KIF2C promotes HCC growth and metastasis via activating MEK/ERK pathway.	In vitro	34494081

2022	Upregulated	Poor RFS Poor DFS Poor OS	Poor differentiation Late TNM stage	RNA-seq Gene microarray IHC qRT-PCR	• KIF2C promoted HCC cell proliferation, migration, invasion, and an accelerated cell cycle and inhibited apoptosis. • KIF2C promoted HCC invasion and metastasis through activation of the EMT. • KIF2C promoted HCC through the Ras/MAPK and PI3K/AKT signaling pathway.	In vitro Omics	35284538
	Upregulated	Poor PFI Poor OS	—	RNA-seq Gene microarray	—	—	36199789
	—	—	—	—	Lactoyl-ph4, dihydrobiopterin, 7-biopterin, and mizoribine may be potential small molecule drug candidates capable of binding to KIF2C.	Molecular docking	36032071

2023	—	—	—	—	ANLN facilitates HCC bone metastases through KIF2C/mTORC1 signaling.	In vitro	36923927

2024	Upregulated	—	—	RNA-seq Gene microarray qRT-PCR	• KIF2C silencing inhibits HCC cell proliferation, migration, and invasion, promotes apoptosis, and keeps the cell cycle in G2 phase. • KIF2C silencing amplifies the sensitivity of HCC cells to cisplatin by regulating the PI3K/AKT/MAPK signaling pathway.	In vitro	38170762

Abbreviations: DFS, disease-free survival; RFS, relapse-free survival; PFI, progress-free interval.

## Data Availability

All data generated or analyzed during this study are included in this published article (and its supporting information files).

## Nomenclature

HCC: hepatocellular carcinoma
MSRRS: molecular subtype–related risk score
MS: molecular subtype
PLC: primary liver cancer
HBV: hepatitis B virus
HCV: hepatitis C virus
AFB1: aflatoxin B1
ICI: immune checkpoint inhibitor
TKI: tyrosine kinase inhibitor
PCA: principal component analysis
TPM: transcripts per million
UMAP: uniform manifold approximation and projection
NTP: nearest template prediction
PAM: partition around medoids
DO: disease ontology
GSVA: gene set variation analysis
GSEA: gene set enrichment analysis
ssGSEA: single-sample gene set enrichment analysis
TF: transcription factors
CV: cross-validation
CCL: cancer cell lines
TME: tumor microenvironment
CDK1: cyclin-dependent kinase 1
OS: overall survival
DFS: disease-free survival
RFS: relapse-free survival
PFI: progress-free interval

## Supporting Information

Supporting Information Additional supporting information can be found online in the Supporting Information section. Figure S1 Multiomics integrative consensus analysis based on the TCGA-LIHC cohort. (A) Evaluation of sample homogeneity through silhouette scores derived from consensus ensemble results. (B, C) PCA results before and after batch correction. (D) Results of DO terms enriched by 200 MS-related marker genes. Figure S2. Genomic landscapes between two HCC MSs. (A, B) GSVA scores for the hallmark gene sets and metabolism-related KEGG pathways. (C) Regulon activity profiles for 23 TFs and potential chromatin remodeling-associated regulators. (D) Immune checkpoint gene expression levels and ssGSEA scores of immune-related pathways. (E) Abundance of different immune cell types estimated by six independent algorithms. Figure S3. Development of MSRRS and its correlation with clinical characteristics. (A) PCA of training and validation cohorts before batch correction. (B) Detailed hazard ratios for 93 prognostic genes. (C) Results of bootstrap resampling of 93 prognostic genes. (D) Feature gene selection based on the Boruta algorithm. Green indicates genes considered important by the Boruta algorithm. (E) Correlation between MSRRS and clinical characteristics. (F, G) Univariate and multivariate Cox regression analysis of MSRRS and clinical characteristics. Figure S4. Molecular interaction networks associated with 10 MSRRS genes obtained from the GeneMANIA database. Figure S5. Correlation analysis of protein expression levels and CERES scores of potential therapeutic targets with MSRRS. (A) Protein expression. (B) CERES scores. Figure S6. Correlation between MSRRS and TME. (A) Differences in expression of various immunomodulators between high- and low-risk groups. (B) Correlation of MSRRS with predicted Treg cell abundance. (C, D) Differences in the activity of immune exclusion signatures and immunotherapy biomarkers between high- and low-risk groups. (E) Correlation of MSRRS with predicted MDSC cell abundance. (F, G) Differences in TMB and TNB between high- and low-risk groups. Figure S7. Potential marker genes for annotation of 24 cell clusters. Figure S8. MSRRS gene expression in HCC tissues and cells. (A, B) Differences in MSRRS gene expression between HCC and normal tissues in TCGA-LIHC cohort. (C) Differences in MSRRS protein expression between HCC and normal tissues in CPTAC HCC cohort. (D) Correlation between MSRRS gene expression and MSRRS in the TCGA-LIHC cohort. (E) Expression levels of KIF2C in different liver cancer cell lines obtained from CCLE database. (F) Details of enrichment of KIF2C-related genes in the cell cycle pathway in HCC. Figure S9. Distribution of two MSs and high and low MSRRS subgroups with proliferation/nonproliferation HCC subclasses in the TCGA-LIHC cohort. Figure S10. MSRRS-related clinical nomogram, calibration curves, and decision curves. Figure S11. Pan-cancer analysis of KIF2C. (A, B) KIF2C mRNA expression levels in the TCGA pan-cancer cohort and protein expression levels in the CPTAC pan-cancer cohort. (C) Correlation of KIF2C mRNA expression with OS in patients with different cancer types in the TCGA pan-cancer cohort. Table S1. Hyperparameters used in the machine learning algorithms. Table S2. The list of curated targetable genes. Table S3. Sequence of RT-qPCR primers and details of siRNAs. Table S4. The results of multiomics consensus ensemble analysis. Table S5. The Top 100 upregulated genes in each subtype. Table S6. The results of univariate Cox analysis of 200 MS-related marker genes. Table S7. The result of bootstrapping-based Cox regression analysis. Table S8. The results of Boruta algorithm to identify genes highly associated with OS. Table S9. The result of machine learning benchmark. Table S10. The result of GSEA. Table S11. Literature review on the role of 10 MSRRS genes in HCC. Table S12. Genes significantly correlated with KIF2C in the TCGA-LIHC cohort. Table S13. The results of KEGG enrichment analysis of KIF2C.
