# Supplementary material for: Consensus Integration of Multiomics Data With Machine Learning Algorithms Reveals Heterogeneous Molecular Subtypes and Enables Personalized Treatment Strategies for Hepatocellular Carcinoma
Source: Hum Mutat. 2025 Nov 24;2025:9967779. doi: 10.1155/humu/9967779 (PMC12668863; doi:10.1155/humu/9967779)
Supplement: Supporting Information — Additional supporting information can be found online in the Supporting Information section. Figure S1 Multiomics integrative consensus analysis based on the TCGA-LIHC cohort. (A) Evaluation of sample homogeneity through silhouette scores derived from consensus ensemble results. (B, C) PCA results before and after batch correction. (D) Results of DO terms enriched by 200 MS-related marker genes. Figure S2. Genomic landscapes between two HCC MSs. (A, B) GSVA scores for the hallmark gene sets and metabolism-related KEGG pathways. (C) Regulon activity profiles for 23 TFs and potential chromatin remodeling-associated regulators. (D) Immune checkpoint gene expression levels and ssGSEA scores of immune-related pathways. (E) Abundance of different immune cell types estimated by six independent algorithms. Figure S3. Development of MSRRS and its correlation with clinical characteristics. (A) PCA of training and validation cohorts before batch correction. (B) Detailed hazard ratios for 93 prognostic genes. (C) Results of bootstrap resampling of 93 prognostic genes. (D) Feature gene selection based on the Boruta algorithm. Green indicates genes considered important by the Boruta algorithm. (E) Correlation between MSRRS and clinical characteristics. (F, G) Univariate and multivariate Cox regression analysis of MSRRS and clinical characteristics. Figure S4. Molecular interaction networks associated with 10 MSRRS genes obtained from the GeneMANIA database. Figure S5. Correlation analysis of protein expression levels and CERES scores of potential therapeutic targets with MSRRS. (A) Protein expression. (B) CERES scores. Figure S6. Correlation between MSRRS and TME. (A) Differences in expression of various immunomodulators between high- and low-risk groups. (B) Correlation of MSRRS with predicted Treg cell abundance. (C, D) Differences in the activity of immune exclusion signatures and immunotherapy biomarkers between high- and low-risk groups. (E) Correlation of MSRRS with predict [file 9967779.f1.zip › Supply/Fig S2.pdf]

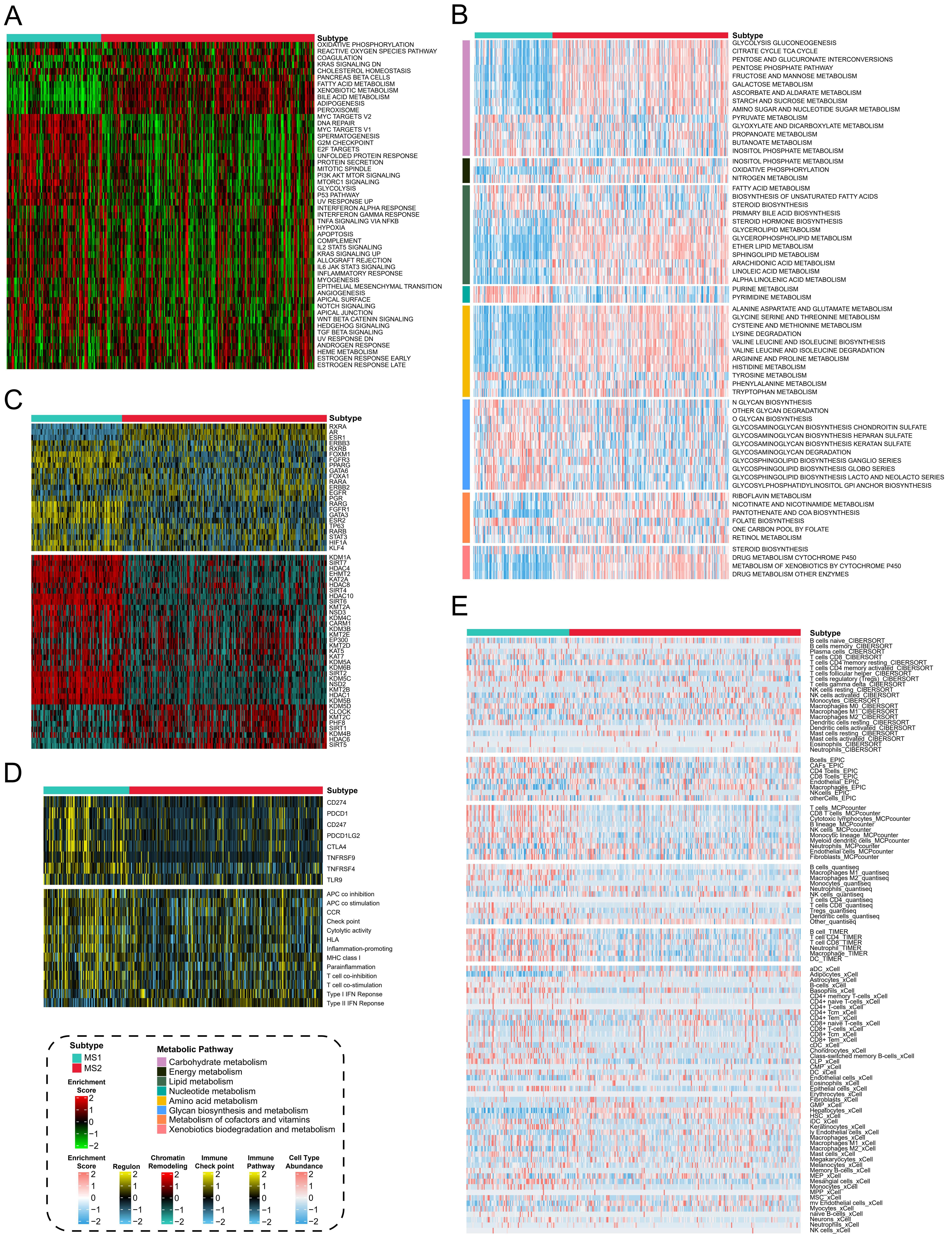
A
Subtype
OXIDATIVE PHOSPHORYLATION
REACTIVE OXYGEN SPECIES PATHWAY
COAGULATION
KRAS SIGNALING DN
CHOLESTEROL HOMEOSTASIS
PANCREAS BETA CELLS
FATTY ACID METABOLISM
XENOBIOTIC METABOLISM
BILE ACID METABOLISM
ADIPOGENESIS
PEROXISOME
MYC TARGETS V2
DNA REPAIR
MYC TARGETS V1
SPERMATOGENESIS
G2M CHECKPOINT
E2F TARGETS
UNFOLDED PROTEIN RESPONSE
PROTEIN SECRETION
MITOTIC SPINDLE
PI3K AKT MTOR SIGNALING
MTORC1 SIGNALING
GLYCOLYSIS
P53 PATHWAY
UV RESPONSE UP
INTERFERON ALPHA RESPONSE
INTERFERON GAMMA RESPONSE
TNFA SIGNALING VIA NFKB
HYPOXIA
APOPTOSIS
COMPLEMENT
IL2 STAT5 SIGNALING
KRAS SIGNALING UP
ALLOGRAFT REJECTION
IL6 JAK STAT3 SIGNALING
INFLAMMATORY RESPONSE
MYOGENESIS
EPITHELIAL MESENCHYMAL TRANSITION
ANGIOGENESIS
APICAL SURFACE
NOTCH SIGNALING
APICAL JUNCTION
WNT BETA CATENIN SIGNALING
HEDGEHOG SIGNALING
TGF BETA SIGNALING
UV RESPONSE DN
ANDROGEN RESPONSE
HEME METABOLISM
ESTROGEN RESPONSE EARLY
ESTROGEN RESPONSE LATE
B
Subtype
GLYCOLYSIS GLUCONEOGENESIS
CITRATE CYCLE TCA CYCLE
PENTOSE AND GLUCURONATE INTERCONVERSIONS
PENTOSE PHOSPHATE PATHWAY
FRUCTOSE AND MANNOSE METABOLISM
GALACTOSE METABOLISM
ASCORBATE AND ALDARATE METABOLISM
STARCH AND SUCROSE METABOLISM
AMINO SUGAR AND NUCLEOTIDE SUGAR METABOLISM
PYRUVATE METABOLISM
GLYOXYLATE AND DICARBOXYLATE METABOLISM
PROPANOATE METABOLISM
BUTANOATE METABOLISM
INOSITOL PHOSPHATE METABOLISM
INOSITOL PHOSPHATE METABOLISM
OXIDATIVE PHOSPHORYLATION
NITROGEN METABOLISM
FATTY ACID METABOLISM
BIOSYNTHESIS OF UNSATURATED FATTY ACIDS
STEROID BIOSYNTHESIS
PRIMARY BILE ACID BIOSYNTHESIS
STEROID HORMONE BIOSYNTHESIS
GLYCEROLIPID METABOLISM
GLYCEROPHOSPHOLIPID METABOLISM
ETHER LIPID METABOLISM
SPHINGOLIPID METABOLISM
ARACHIDONIC ACID METABOLISM
LINOLEIC ACID METABOLISM
ALPHA LINOLENIC ACID METABOLISM
PURINE METABOLISM
PYRIMIDINE METABOLISM
ALANINE ASPARTATE AND GLUTAMATE METABOLISM
GLYCINE SERINE AND THREONINE METABOLISM
CYSTEINE AND METHIONINE METABOLISM
LYSINE DEGRADATION
VALINE LEUCINE AND ISOLEUCINE BIOSYNTHESIS
VALINE LEUCINE AND ISOLEUCINE DEGRADATION
ARGININE AND PROLINE METABOLISM
HISTIDINE METABOLISM
TYROSINE METABOLISM
PHENYLALANINE METABOLISM
TRYPTOPHAN METABOLISM
N GLYCAN BIOSYNTHESIS
OTHER GLYCAN DEGRADATION
O GLYCAN BIOSYNTHESIS
GLYCOSAMINOGLYCAN BIOSYNTHESIS CHONDROITIN SULFATE
GLYCOSAMINOGLYCAN BIOSYNTHESIS HEPARAN SULFATE
GLYCOSAMINOGLYCAN BIOSYNTHESIS KERATAN SULFATE
GLYCOSAMINOGLYCAN DEGRADATION
GLYCOSPHINGOLIPID BIOSYNTHESIS GANGLIO SERIES
GLYCOSPHINGOLIPID BIOSYNTHESIS GLOBO SERIES
GLYCOSPHINGOLIPID BIOSYNTHESIS LACTO AND NEOLACTO SERIES
GLYCOSYLPHOSPHATIDYLINOSITOL GPI ANCHOR BIOSYNTHESIS
RIBOFLAVIN METABOLISM
NICOTINATE AND NICOTINAMIDE METABOLISM
PANTOTHENATE AND COA BIOSYNTHESIS
FOLATE BIOSYNTHESIS
ONE CARBON POOL BY FOLATE
RETINOL METABOLISM
STEROID BIOSYNTHESIS
DRUG METABOLISM CYTOCHROME P450
METABOLISM OF XENOBIOTICS BY CYTOCHROME P450
DRUG METABOLISM OTHER ENZYMES
C
Subtype
RXRA
AR
ESR1
ERBB3
RXRB
FOXM1
FGFR3
PPARG
GATA6
FOXA1
RARA
ERBB2
EGFR
PGR
RARG
FGFR1
GATA3
ESR2
TP63
RARB
STAT3
HIF1A
KLF4
KDM1A
SIRT7
HDAC4
EHMT2
KAT2A
HDAC8
SIRT4
HDAC10
SIRT6
KMT2A
NSD3
KDM4C
CARM1
KDM3B
KMT2E
EP300
KMT2D
KAT5
KAT7
KDM5A
KDM6B
KDM5C
NSD2
KMT2B
HDAC1
KDM5B
KDM5D
CLOCK
KMT2C
PHF8
SIRT1
KDM4B
HDAC6
SIRT5
D
Subtype
CD274
PDCD1
CD247
PDCD1LG2
CTLA4
TNFRSF9
TNFRSF4
TLR9
APC co inhibition
APC co stimulation
CCR
Check point
Cytolytic activity
HLA
Inflammation-promoting
MHC class I
Parainflammation
T cell co-inhibition
T cell co-stimulation
Type I IFN Reponse
Type II IFN Reponse
E
Subtype
B cells naive_CIBERSORT
B cells memory_CIBERSORT
Plasma cells_CIBERSORT
T cells CD8_CIBERSORT
T cells CD4 memory resting_CIBERSORT
T cells CD4 memory activated_CIBERSORT
T cells follicular helper_CIBERSORT
T cells regulatory (Tregs)_CIBERSORT
T cells gamma delta_CIBERSORT
NK cells resting_CIBERSORT
NK cells activated_CIBERSORT
Monocytes_CIBERSORT
Macrophages M0_CIBERSORT
Macrophages M1_CIBERSORT
Macrophages M2_CIBERSORT
Dendritic cells resting_CIBERSORT
Dendritic cells activated_CIBERSORT
Mast cells resting_CIBERSORT
Mast cells activated_CIBERSORT
Eosinophils_CIBERSORT
Neutrophils_CIBERSORT
Bcells_EPIC
CAFs_EPIC
CD4 Tcells_EPIC
CD8 Tcells_EPIC
Endothelial_EPIC
Macrophages_EPIC
NKcells_EPIC
otherCells_EPIC
T cells_MCPcounter
CD8 T cells_MCPcounter
Cytotoxic lymphocytes_MCPcounter
B lineage_MCPcounter
NK cells_MCPcounter
Monocytic lineage_MCPcounter
Myeloid dendritic cells_MCPcounter
Neutrophils_MCPcounter
Endothelial cells_MCPcounter
Fibroblasts_MCPcounter
B cells_quantiseq
Macrophages M1_quantiseq
Macrophages M2_quantiseq
Monocytes_quantiseq
Neutrophils_quantiseq
NK cells_quantiseq
T cells CD4_quantiseq
T cells CD8_quantiseq
Tregs_quantiseq
Dendritic cells_quantiseq
Other_quantiseq
B cell_TIMER
T cell CD4_TIMER
T cell CD8_TIMER
Neutrophil_TIMER
Macrophage_TIMER
DC_TIMER
aDC_xCell
Adipocytes_xCell
Astrocytes_xCell
B-cells_xCell
Basophils_xCell
CD4+ memory T-cells_xCell
CD4+ naive T-cells_xCell
CD4+ T-cells_xCell
CD4+ Tcm_xCell
CD4+ Tem_xCell
CD8+ naive T-cells_xCell
CD8+ T-cells_xCell
CD8+ Tcm_xCell
CD8+ Tem_xCell
cDC_xCell
Chondrocytes_xCell
Class-switched memory B-cells_xCell
CLP_xCell
CMP_xCell
DC_xCell
Endothelial cells_xCell
Eosinophils_xCell
Epithelial cells_xCell
Erythrocytes_xCell
Fibroblasts_xCell
GMP_xCell
Hepatocytes_xCell
HSC_xCell
iDC_xCell
Keratinocytes_xCell
ly Endothelial cells_xCell
Macrophages_xCell
Macrophages M1_xCell
Macrophages M2_xCell
Mast cells_xCell
Megakaryocytes_xCell
Melanocytes_xCell
Memory B-cells_xCell
MEP_xCell
Mesangial cells_xCell
Monocytes_xCell
MPP_xCell
MSC_xCell
mv Endothelial cells_xCell
Myocytes_xCell
naive B-cells_xCell
Neurons_xCell
Neutrophils_xCell
NK cells_xCell
Subtype
MS1
MS2
Enrichment Score
2
1
0
−1
−2
Metabolic Pathway
Carbohydrate metabolism
Energy metabolism
Lipid metabolism
Nucleotide metabolism
Amino acid metabolism
Glycan biosynthesis and metabolism
Metabolism of cofactors and vitamins
Xenobiotics biodegradation and metabolism
Enrichment Score
Regulon
Chromatin Remodeling
Immune Check point
Immune Pathway
Cell Type Abundance
2
1
0
−1
−2
