# Supplementary figures and images for: Consensus Integration of Multiomics Data With Machine Learning Algorithms Reveals Heterogeneous Molecular Subtypes and Enables Personalized Treatment Strategies for Hepatocellular Carcinoma
Source: Hum Mutat. 2025 Nov 24;2025:9967779. doi: 10.1155/humu/9967779 (PMC12668863; doi:10.1155/humu/9967779)

A

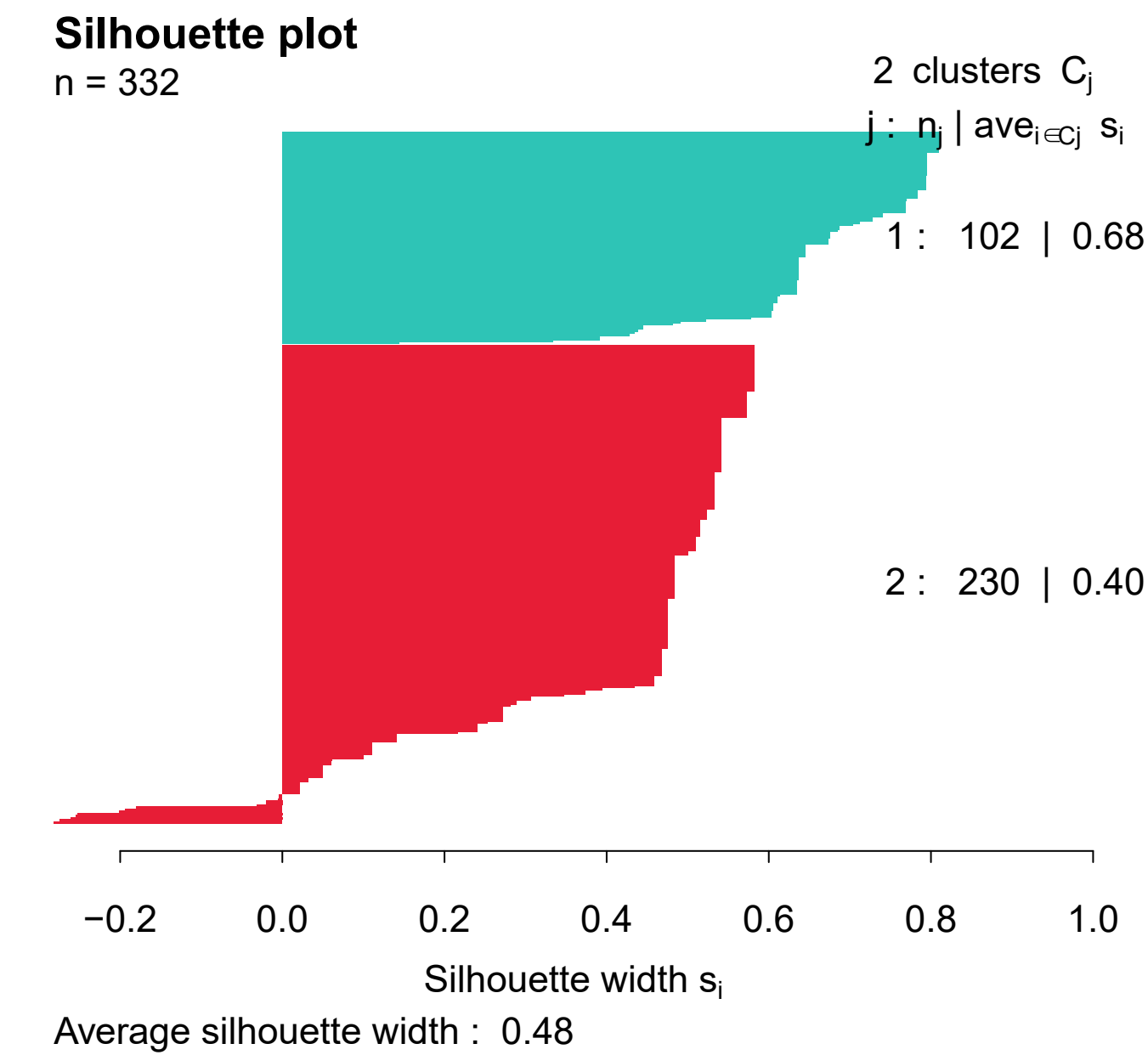

B

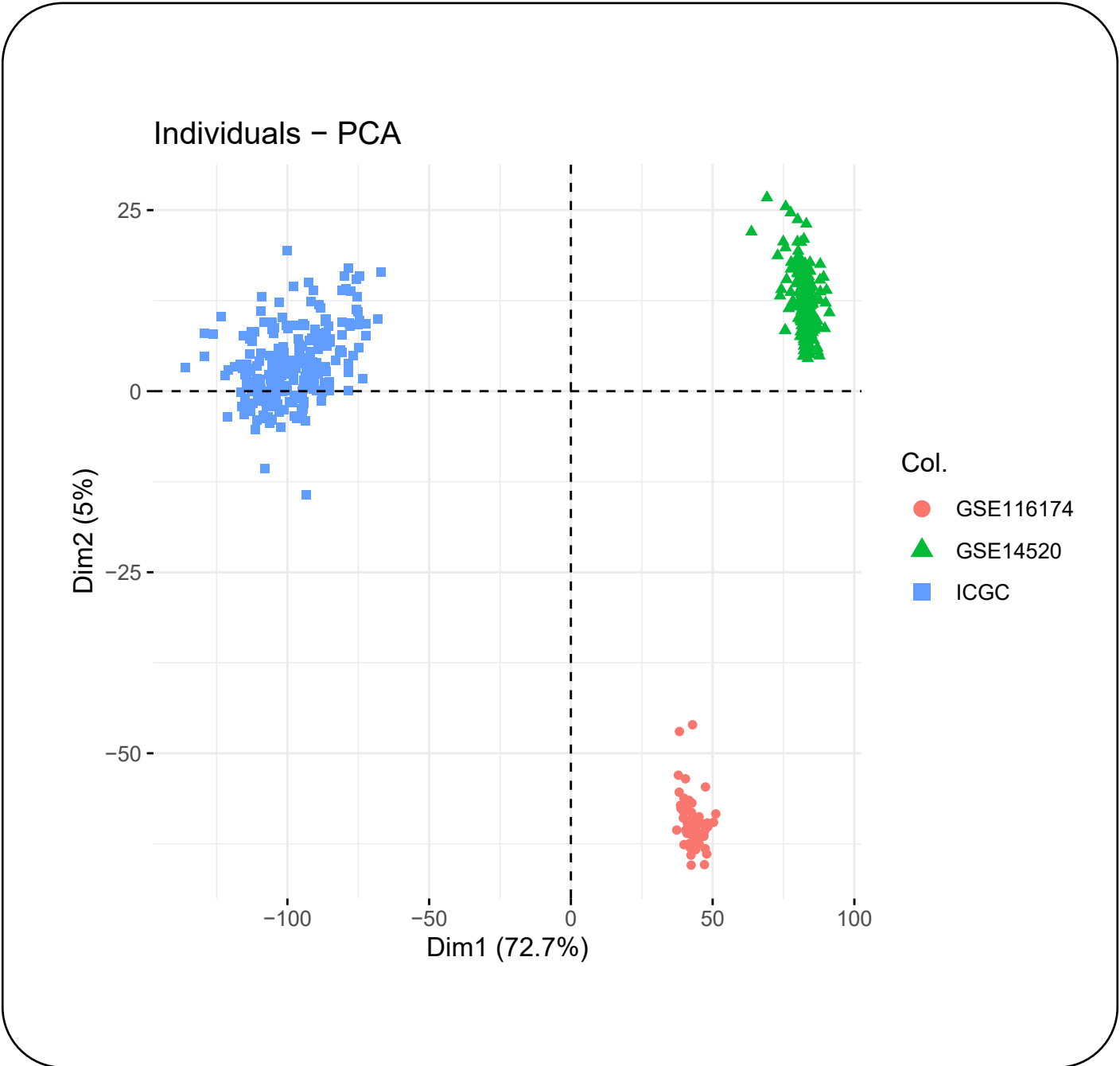

C

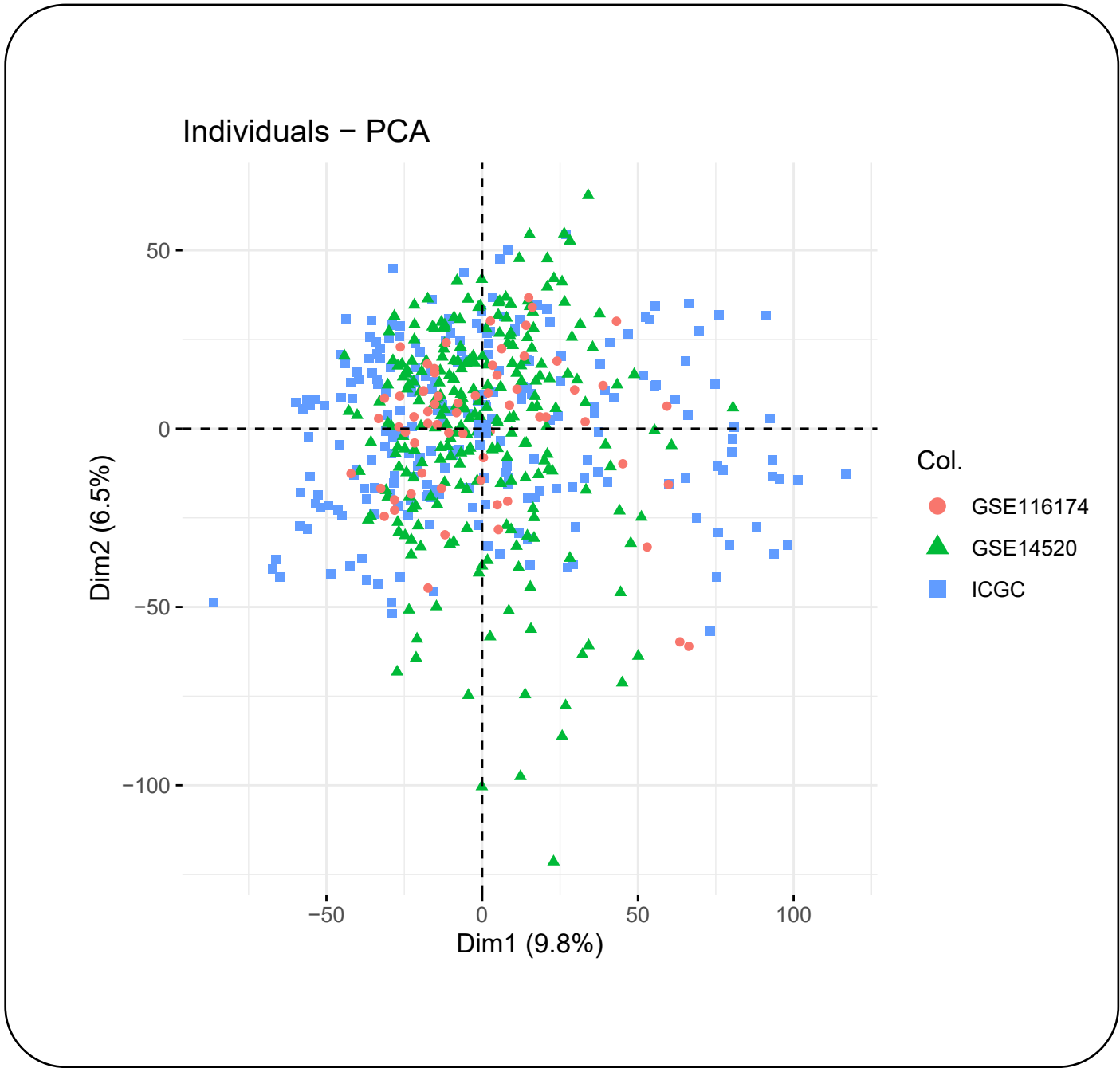

D

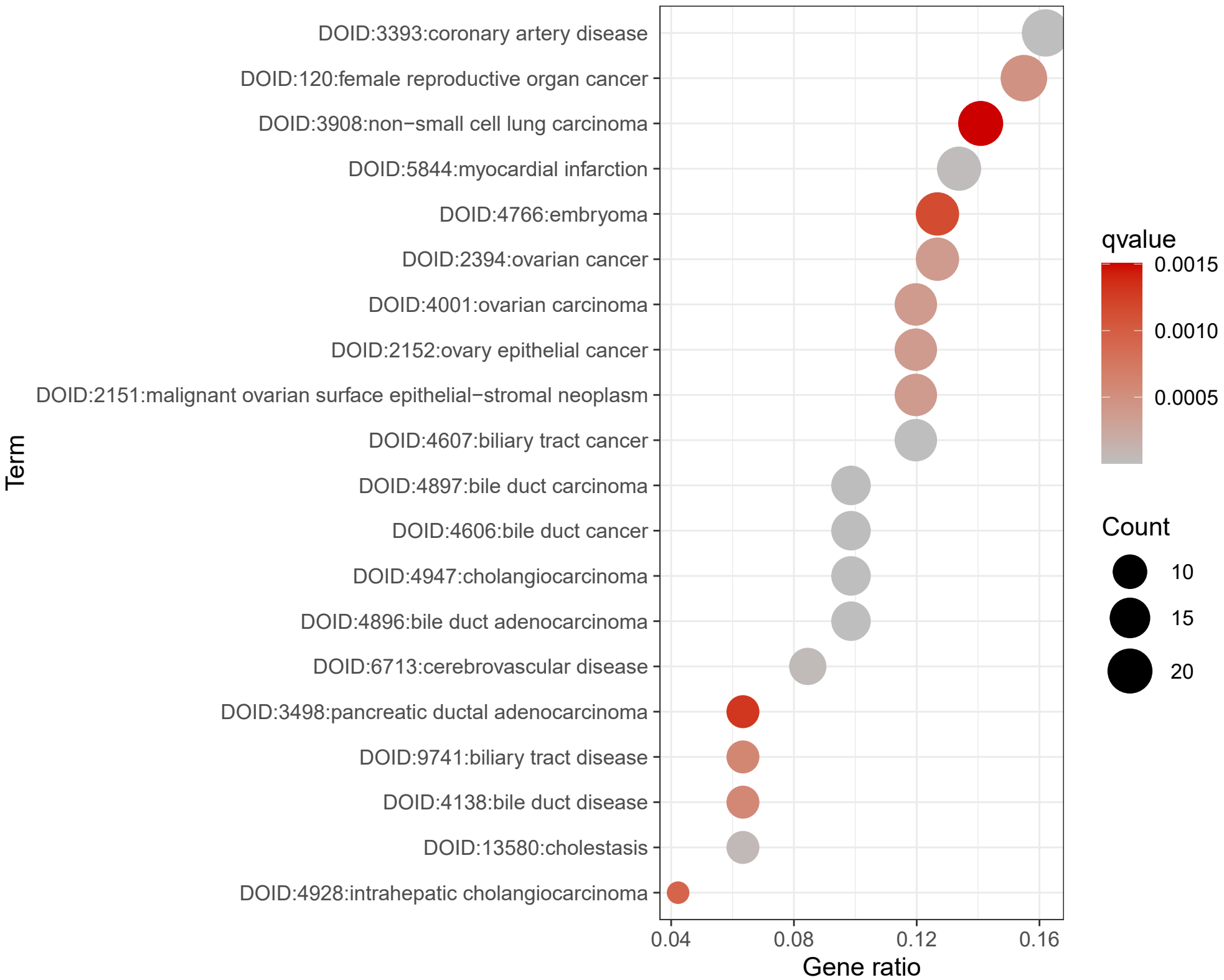

Supplement: Supporting Information — Additional supporting information can be found online in the Supporting Information section. Figure S1 Multiomics integrative consensus analysis based on the TCGA-LIHC cohort. (A) Evaluation of sample homogeneity through silhouette scores derived from consensus ensemble results. (B, C) PCA results before and after batch correction. (D) Results of DO terms enriched by 200 MS-related marker genes. Figure S2. Genomic landscapes between two HCC MSs. (A, B) GSVA scores for the hallmark gene sets and metabolism-related KEGG pathways. (C) Regulon activity profiles for 23 TFs and potential chromatin remodeling-associated regulators. (D) Immune checkpoint gene expression levels and ssGSEA scores of immune-related pathways. (E) Abundance of different immune cell types estimated by six independent algorithms. Figure S3. Development of MSRRS and its correlation with clinical characteristics. (A) PCA of training and validation cohorts before batch correction. (B) Detailed hazard ratios for 93 prognostic genes. (C) Results of bootstrap resampling of 93 prognostic genes. (D) Feature gene selection based on the Boruta algorithm. Green indicates genes considered important by the Boruta algorithm. (E) Correlation between MSRRS and clinical characteristics. (F, G) Univariate and multivariate Cox regression analysis of MSRRS and clinical characteristics. Figure S4. Molecular interaction networks associated with 10 MSRRS genes obtained from the GeneMANIA database. Figure S5. Correlation analysis of protein expression levels and CERES scores of potential therapeutic targets with MSRRS. (A) Protein expression. (B) CERES scores. Figure S6. Correlation between MSRRS and TME. (A) Differences in expression of various immunomodulators between high- and low-risk groups. (B) Correlation of MSRRS with predicted Treg cell abundance. (C, D) Differences in the activity of immune exclusion signatures and immunotherapy biomarkers between high- and low-risk groups. (E) Correlation of MSRRS with predict [file 9967779.f1.zip › Supply/Fig S1.pdf]

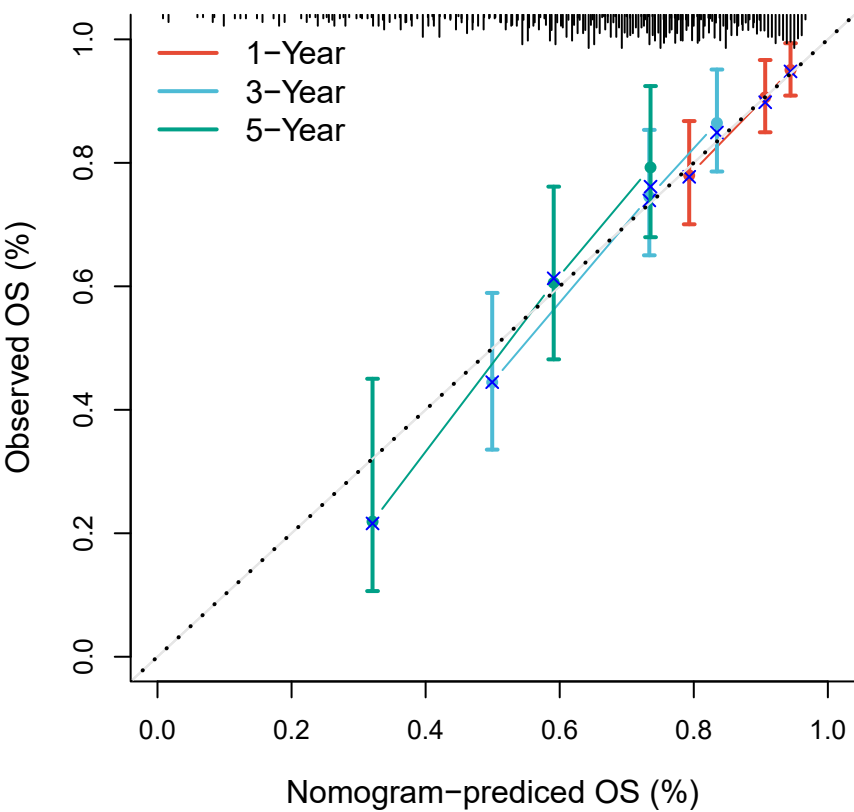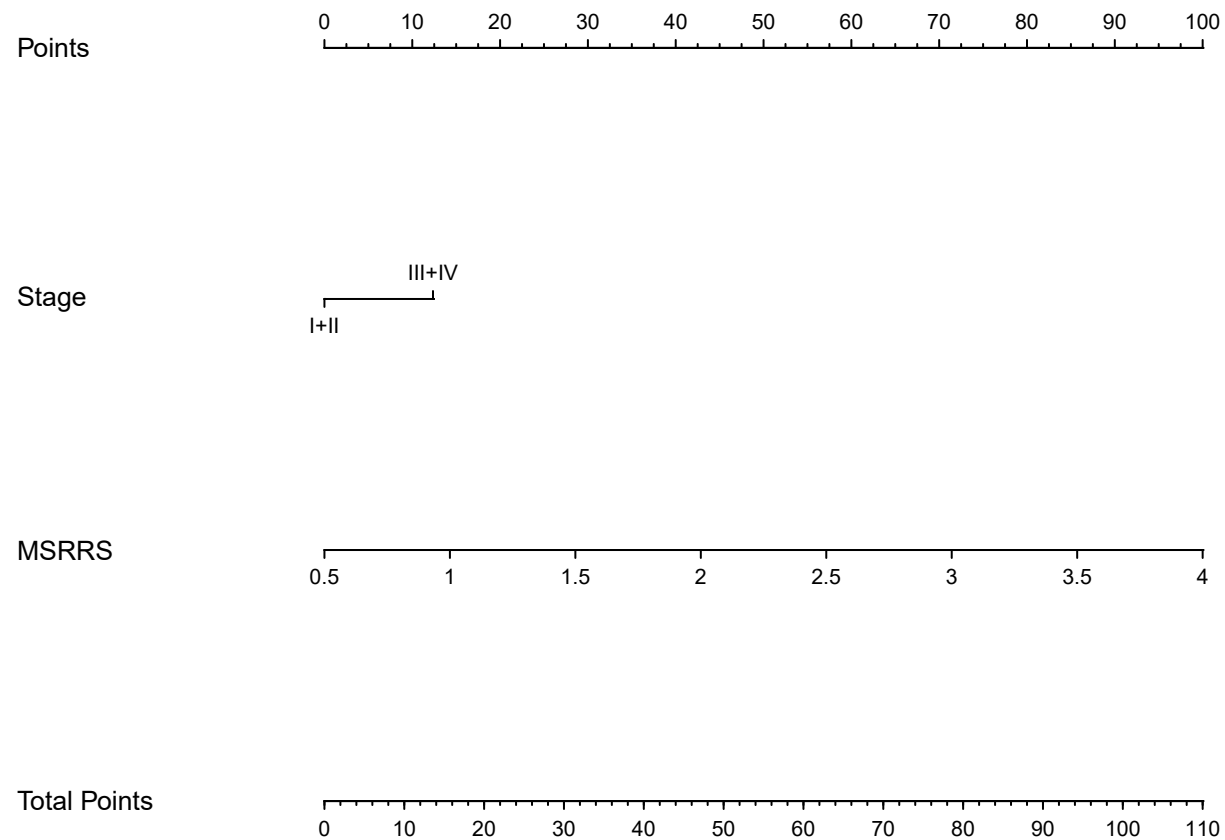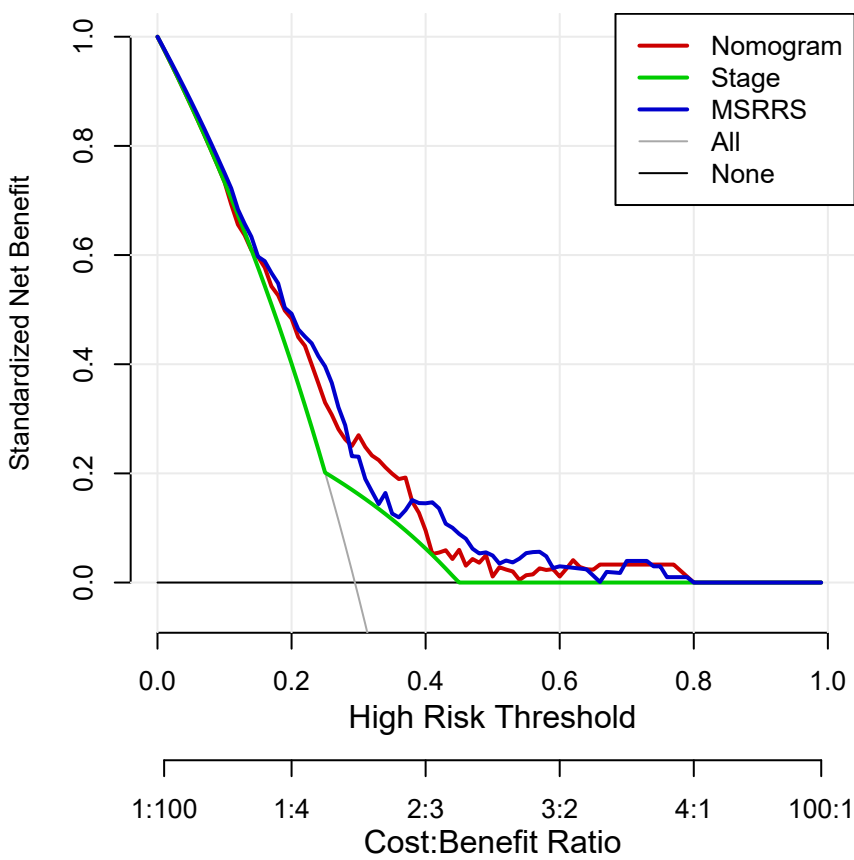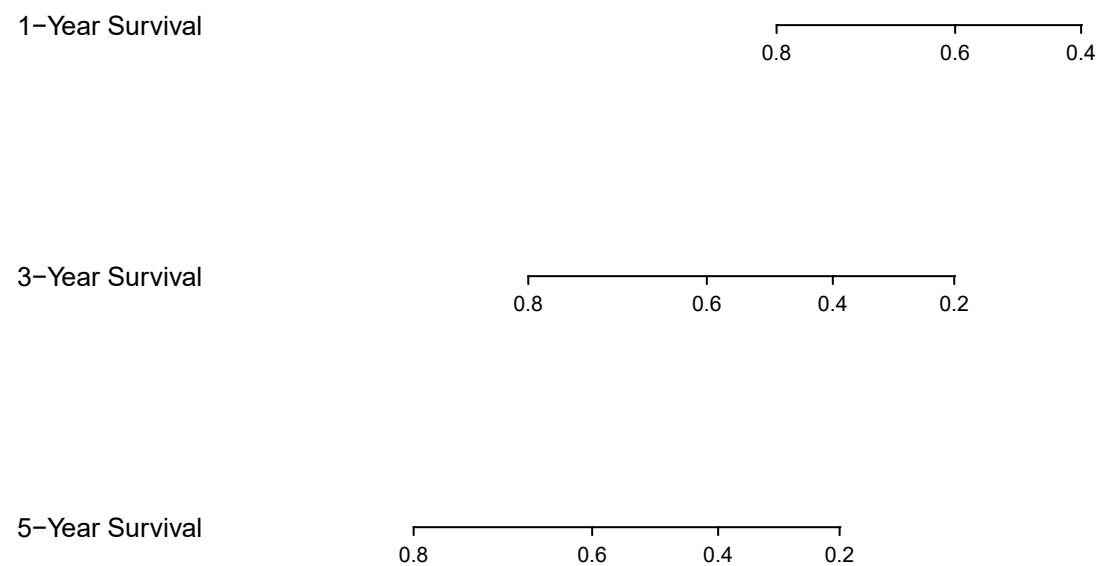

Supplement: Supporting Information — Additional supporting information can be found online in the Supporting Information section. Figure S1 Multiomics integrative consensus analysis based on the TCGA-LIHC cohort. (A) Evaluation of sample homogeneity through silhouette scores derived from consensus ensemble results. (B, C) PCA results before and after batch correction. (D) Results of DO terms enriched by 200 MS-related marker genes. Figure S2. Genomic landscapes between two HCC MSs. (A, B) GSVA scores for the hallmark gene sets and metabolism-related KEGG pathways. (C) Regulon activity profiles for 23 TFs and potential chromatin remodeling-associated regulators. (D) Immune checkpoint gene expression levels and ssGSEA scores of immune-related pathways. (E) Abundance of different immune cell types estimated by six independent algorithms. Figure S3. Development of MSRRS and its correlation with clinical characteristics. (A) PCA of training and validation cohorts before batch correction. (B) Detailed hazard ratios for 93 prognostic genes. (C) Results of bootstrap resampling of 93 prognostic genes. (D) Feature gene selection based on the Boruta algorithm. Green indicates genes considered important by the Boruta algorithm. (E) Correlation between MSRRS and clinical characteristics. (F, G) Univariate and multivariate Cox regression analysis of MSRRS and clinical characteristics. Figure S4. Molecular interaction networks associated with 10 MSRRS genes obtained from the GeneMANIA database. Figure S5. Correlation analysis of protein expression levels and CERES scores of potential therapeutic targets with MSRRS. (A) Protein expression. (B) CERES scores. Figure S6. Correlation between MSRRS and TME. (A) Differences in expression of various immunomodulators between high- and low-risk groups. (B) Correlation of MSRRS with predicted Treg cell abundance. (C, D) Differences in the activity of immune exclusion signatures and immunotherapy biomarkers between high- and low-risk groups. (E) Correlation of MSRRS with predict [file 9967779.f1.zip › Supply/Fig S10.pdf]

A

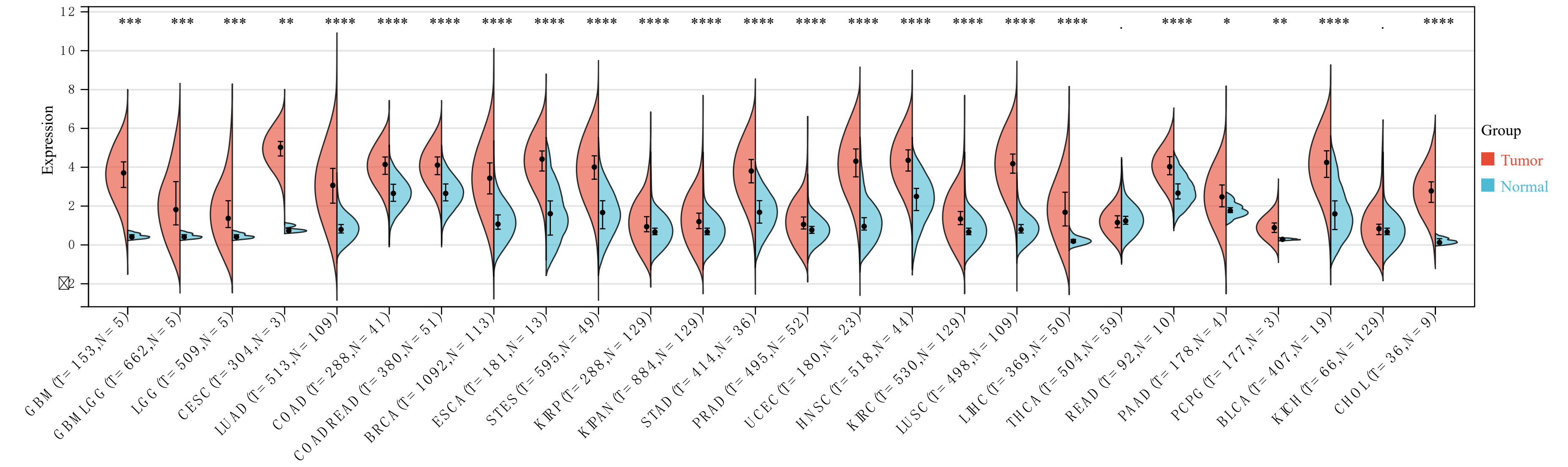

B

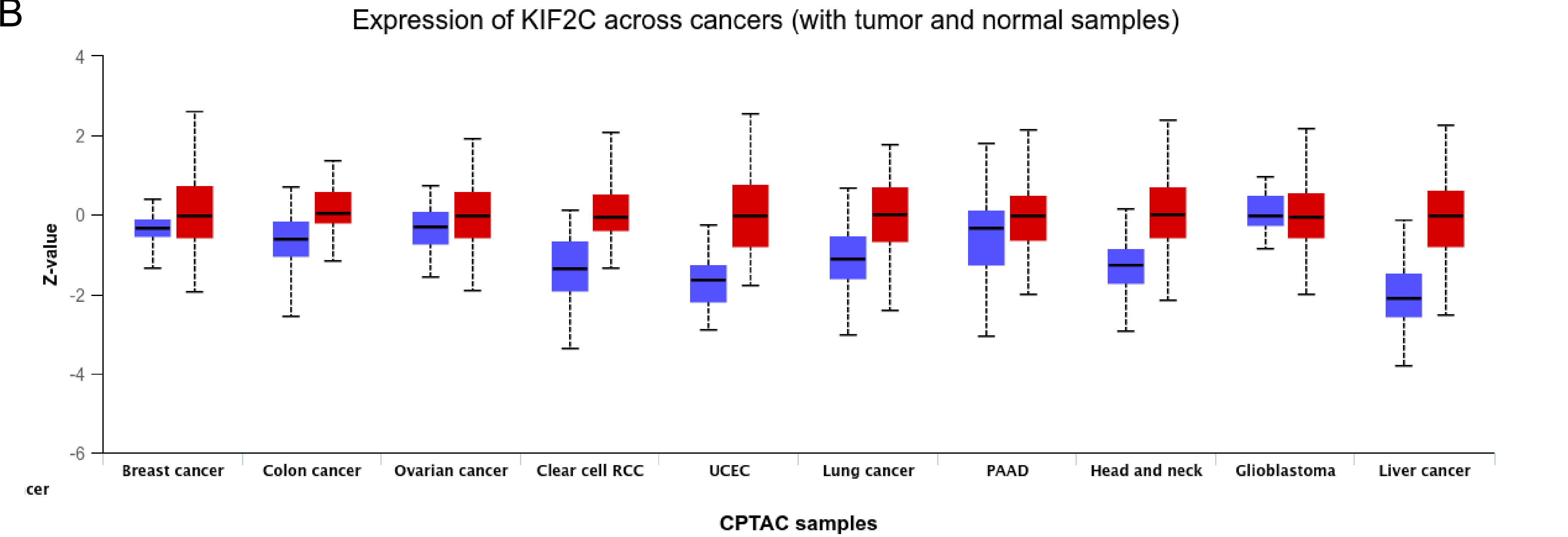

C

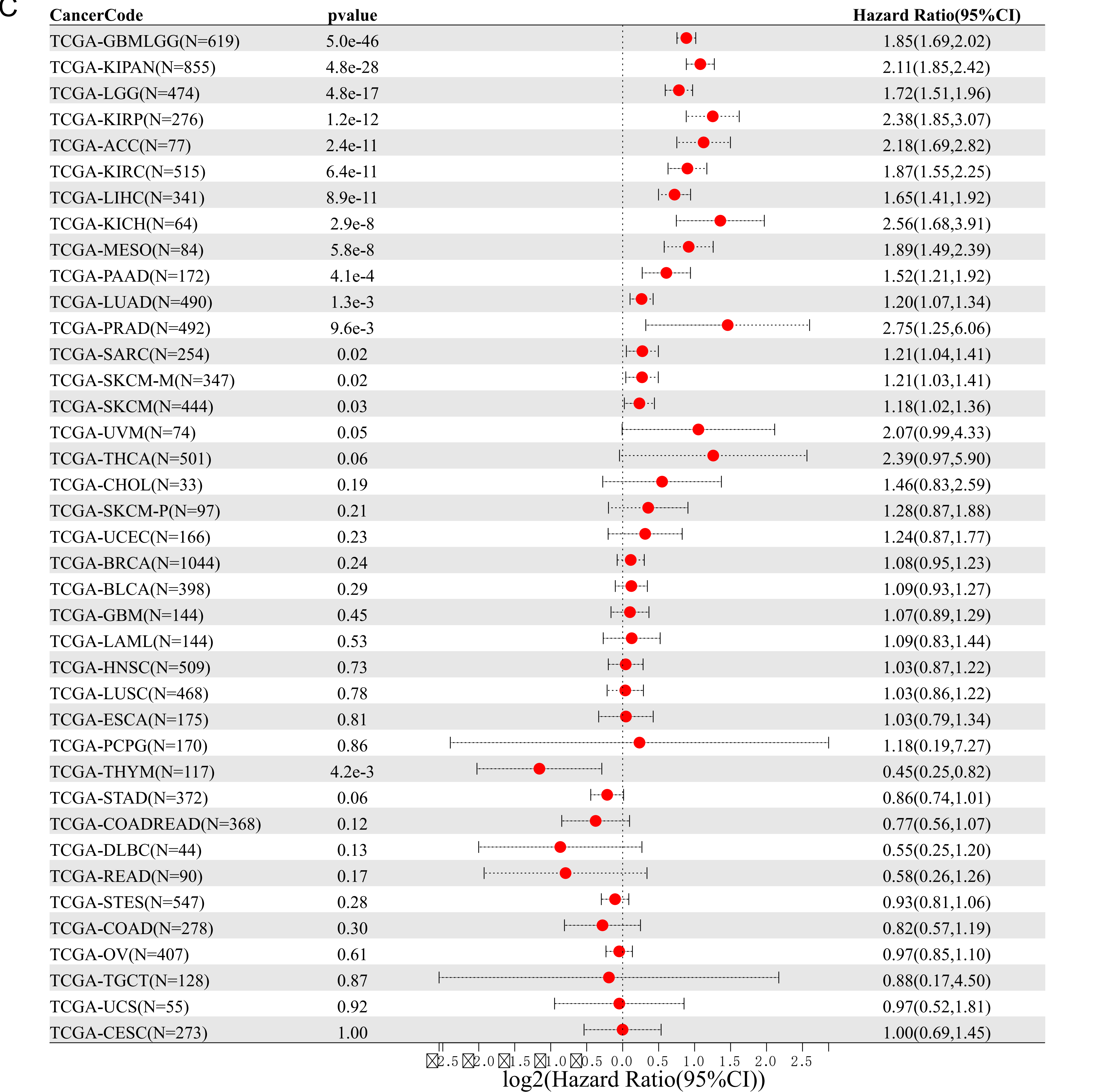

Supplement: Supporting Information — Additional supporting information can be found online in the Supporting Information section. Figure S1 Multiomics integrative consensus analysis based on the TCGA-LIHC cohort. (A) Evaluation of sample homogeneity through silhouette scores derived from consensus ensemble results. (B, C) PCA results before and after batch correction. (D) Results of DO terms enriched by 200 MS-related marker genes. Figure S2. Genomic landscapes between two HCC MSs. (A, B) GSVA scores for the hallmark gene sets and metabolism-related KEGG pathways. (C) Regulon activity profiles for 23 TFs and potential chromatin remodeling-associated regulators. (D) Immune checkpoint gene expression levels and ssGSEA scores of immune-related pathways. (E) Abundance of different immune cell types estimated by six independent algorithms. Figure S3. Development of MSRRS and its correlation with clinical characteristics. (A) PCA of training and validation cohorts before batch correction. (B) Detailed hazard ratios for 93 prognostic genes. (C) Results of bootstrap resampling of 93 prognostic genes. (D) Feature gene selection based on the Boruta algorithm. Green indicates genes considered important by the Boruta algorithm. (E) Correlation between MSRRS and clinical characteristics. (F, G) Univariate and multivariate Cox regression analysis of MSRRS and clinical characteristics. Figure S4. Molecular interaction networks associated with 10 MSRRS genes obtained from the GeneMANIA database. Figure S5. Correlation analysis of protein expression levels and CERES scores of potential therapeutic targets with MSRRS. (A) Protein expression. (B) CERES scores. Figure S6. Correlation between MSRRS and TME. (A) Differences in expression of various immunomodulators between high- and low-risk groups. (B) Correlation of MSRRS with predicted Treg cell abundance. (C, D) Differences in the activity of immune exclusion signatures and immunotherapy biomarkers between high- and low-risk groups. (E) Correlation of MSRRS with predict [file 9967779.f1.zip › Supply/Fig S11.pdf]

A

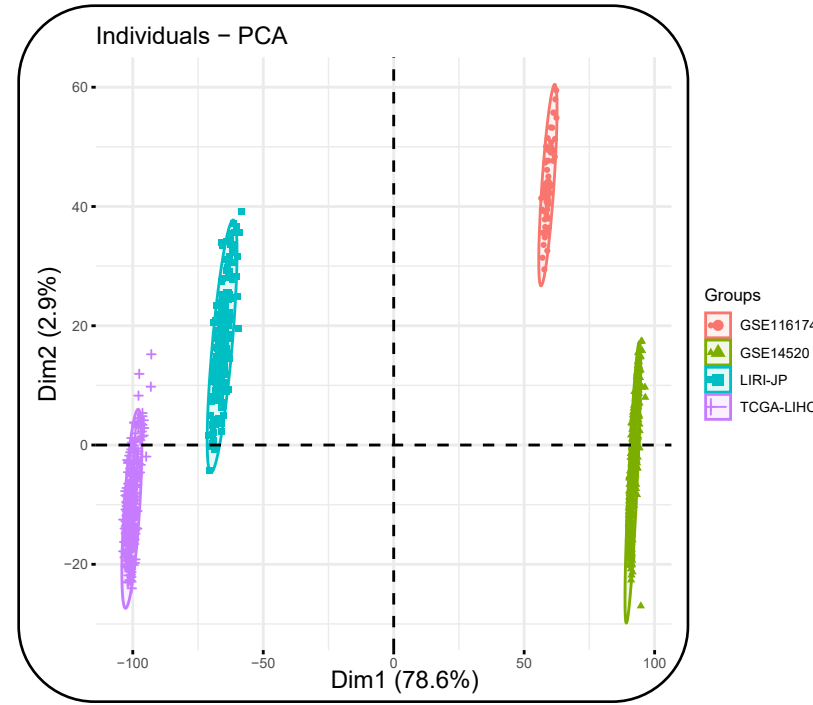

B

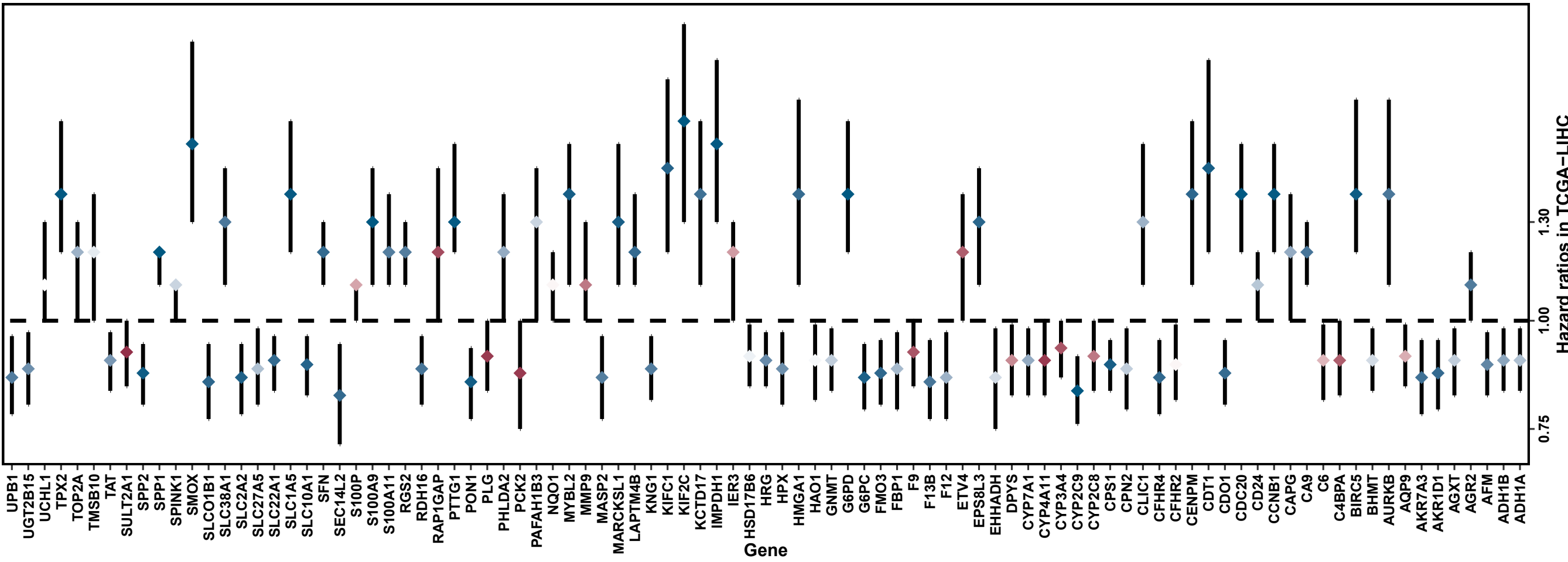

C

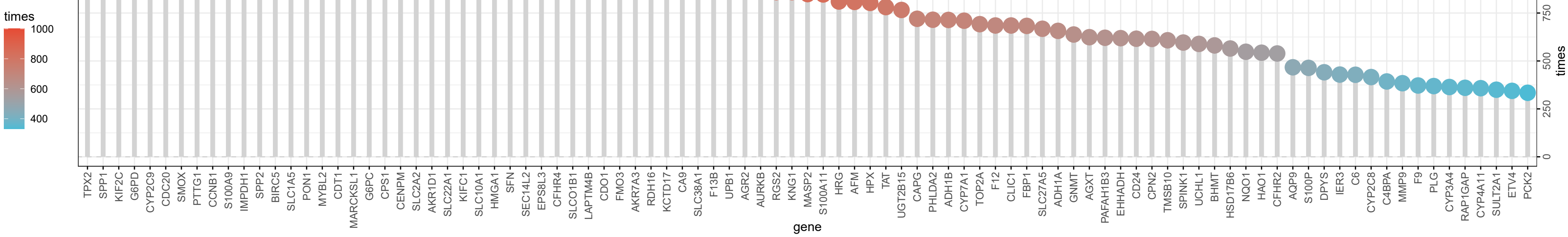

D

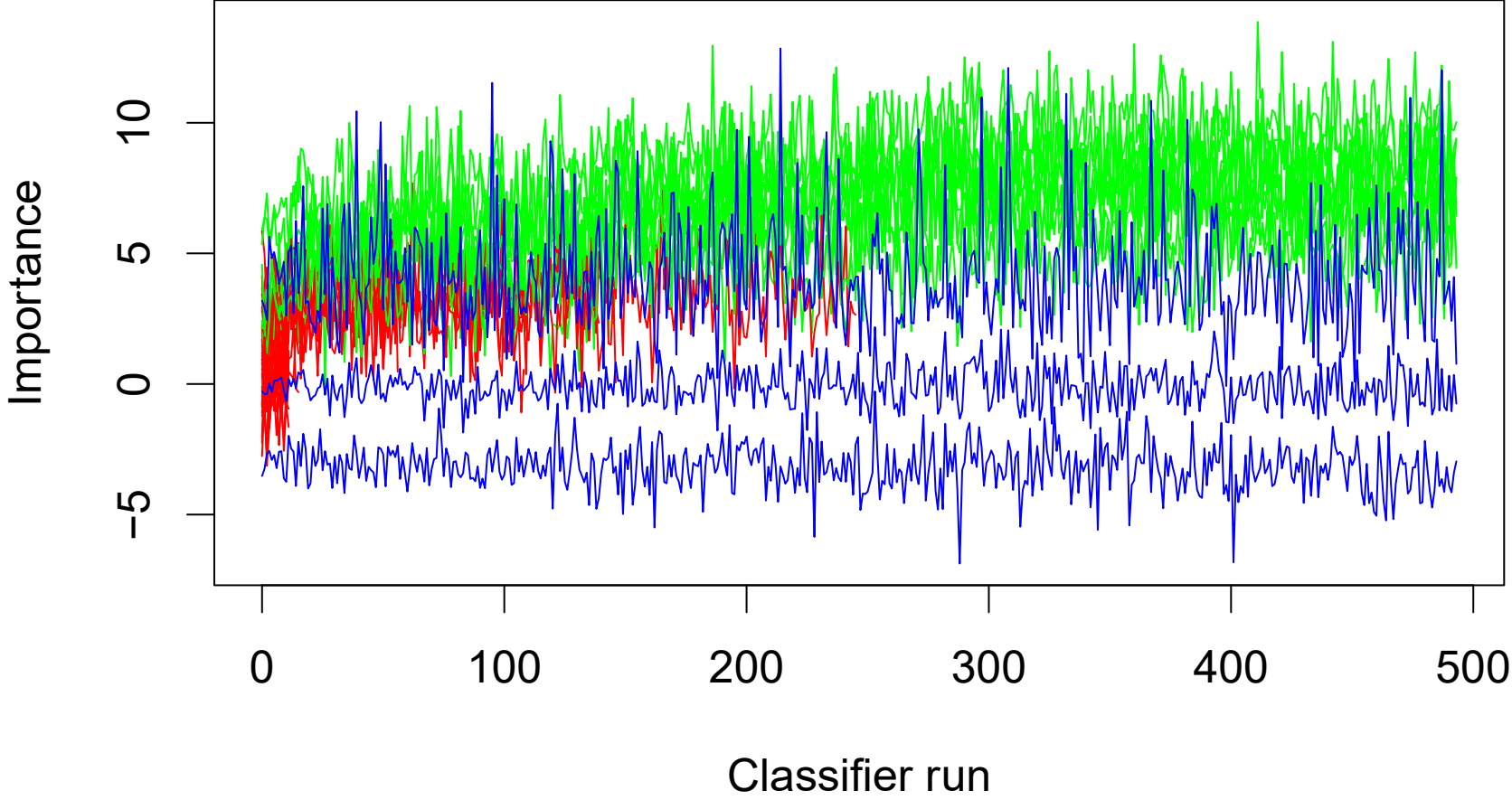

E

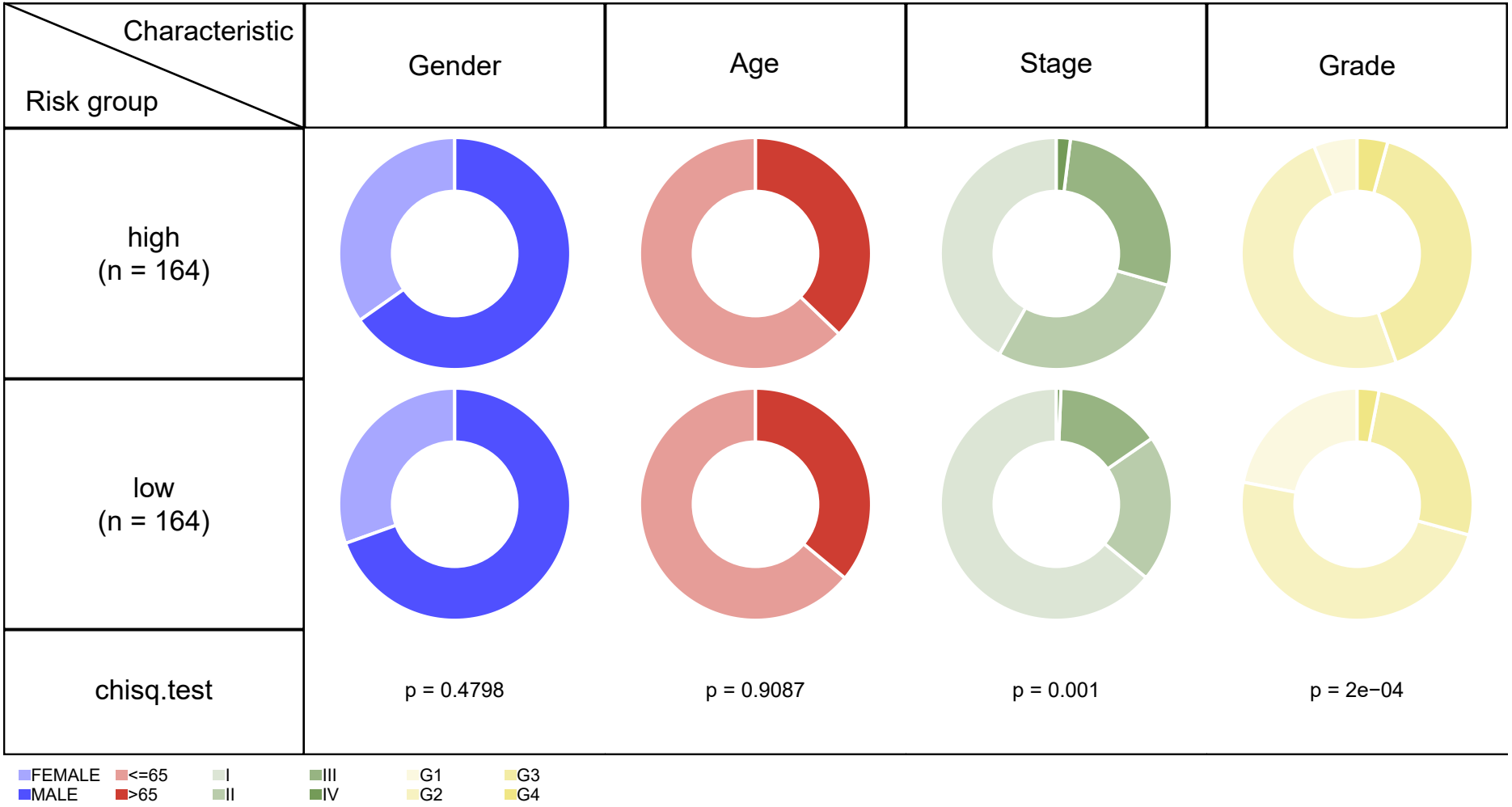

F

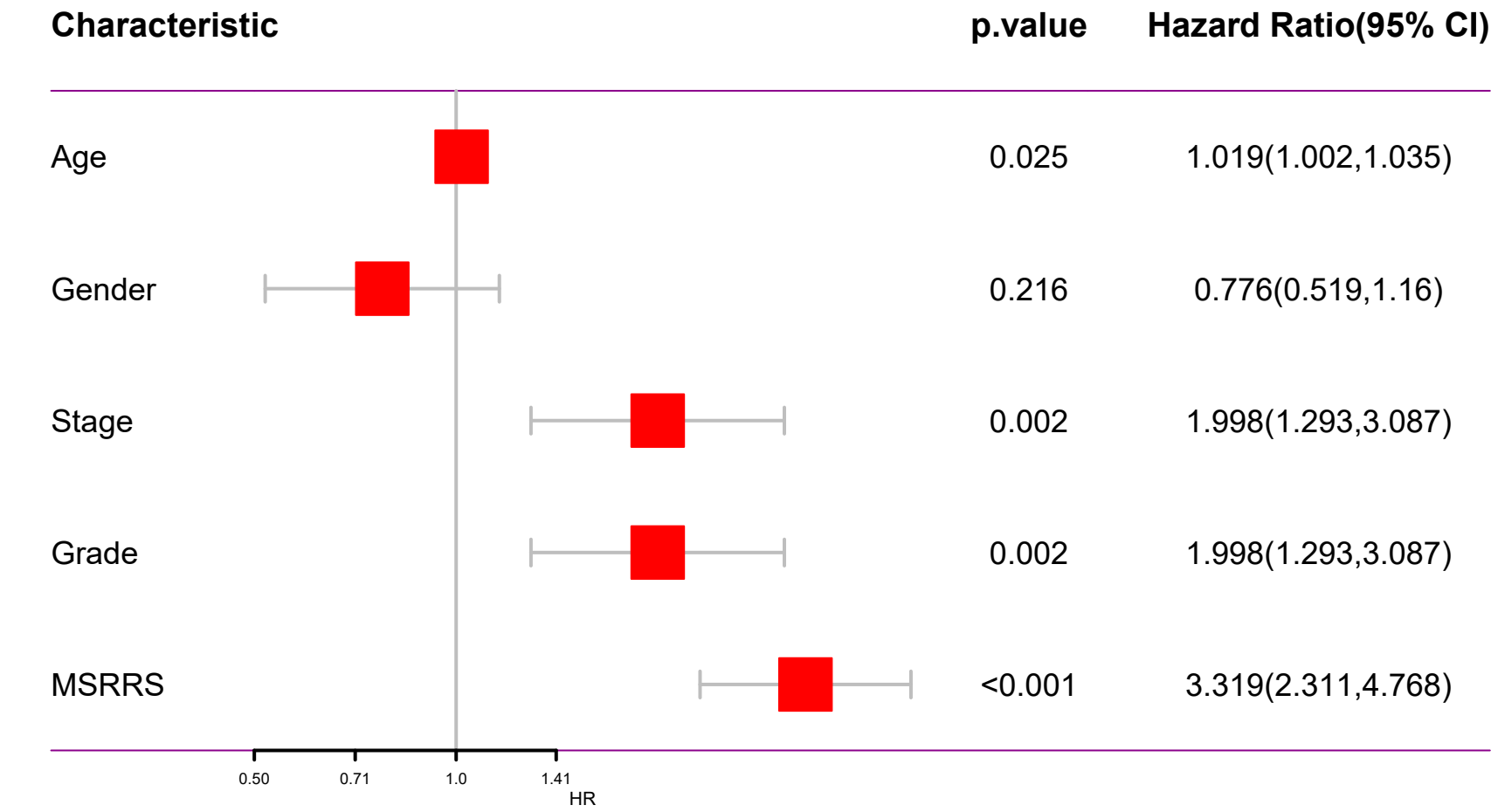

G

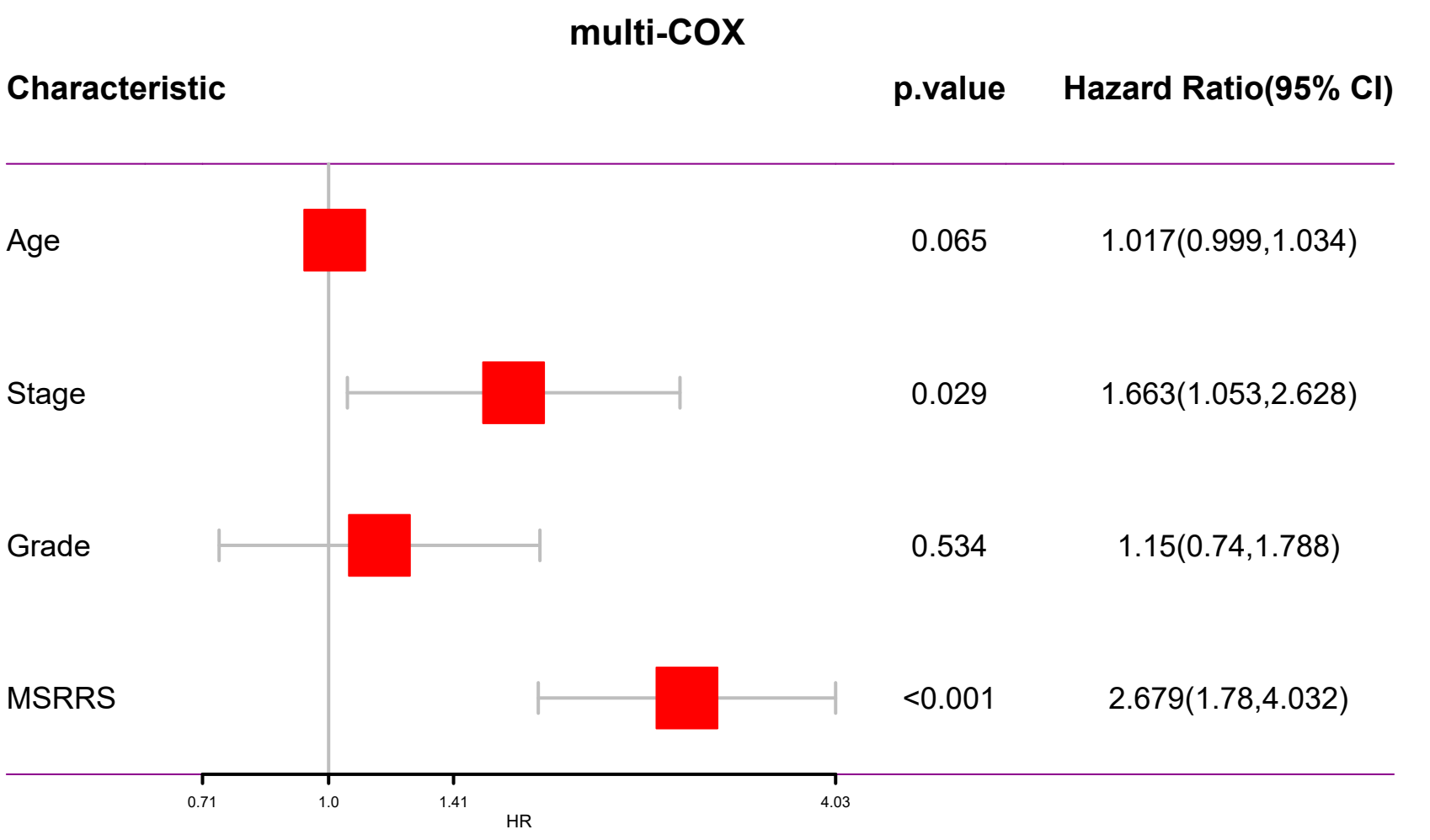

Supplement: Supporting Information — Additional supporting information can be found online in the Supporting Information section. Figure S1 Multiomics integrative consensus analysis based on the TCGA-LIHC cohort. (A) Evaluation of sample homogeneity through silhouette scores derived from consensus ensemble results. (B, C) PCA results before and after batch correction. (D) Results of DO terms enriched by 200 MS-related marker genes. Figure S2. Genomic landscapes between two HCC MSs. (A, B) GSVA scores for the hallmark gene sets and metabolism-related KEGG pathways. (C) Regulon activity profiles for 23 TFs and potential chromatin remodeling-associated regulators. (D) Immune checkpoint gene expression levels and ssGSEA scores of immune-related pathways. (E) Abundance of different immune cell types estimated by six independent algorithms. Figure S3. Development of MSRRS and its correlation with clinical characteristics. (A) PCA of training and validation cohorts before batch correction. (B) Detailed hazard ratios for 93 prognostic genes. (C) Results of bootstrap resampling of 93 prognostic genes. (D) Feature gene selection based on the Boruta algorithm. Green indicates genes considered important by the Boruta algorithm. (E) Correlation between MSRRS and clinical characteristics. (F, G) Univariate and multivariate Cox regression analysis of MSRRS and clinical characteristics. Figure S4. Molecular interaction networks associated with 10 MSRRS genes obtained from the GeneMANIA database. Figure S5. Correlation analysis of protein expression levels and CERES scores of potential therapeutic targets with MSRRS. (A) Protein expression. (B) CERES scores. Figure S6. Correlation between MSRRS and TME. (A) Differences in expression of various immunomodulators between high- and low-risk groups. (B) Correlation of MSRRS with predicted Treg cell abundance. (C, D) Differences in the activity of immune exclusion signatures and immunotherapy biomarkers between high- and low-risk groups. (E) Correlation of MSRRS with predict [file 9967779.f1.zip › Supply/Fig S3.pdf]

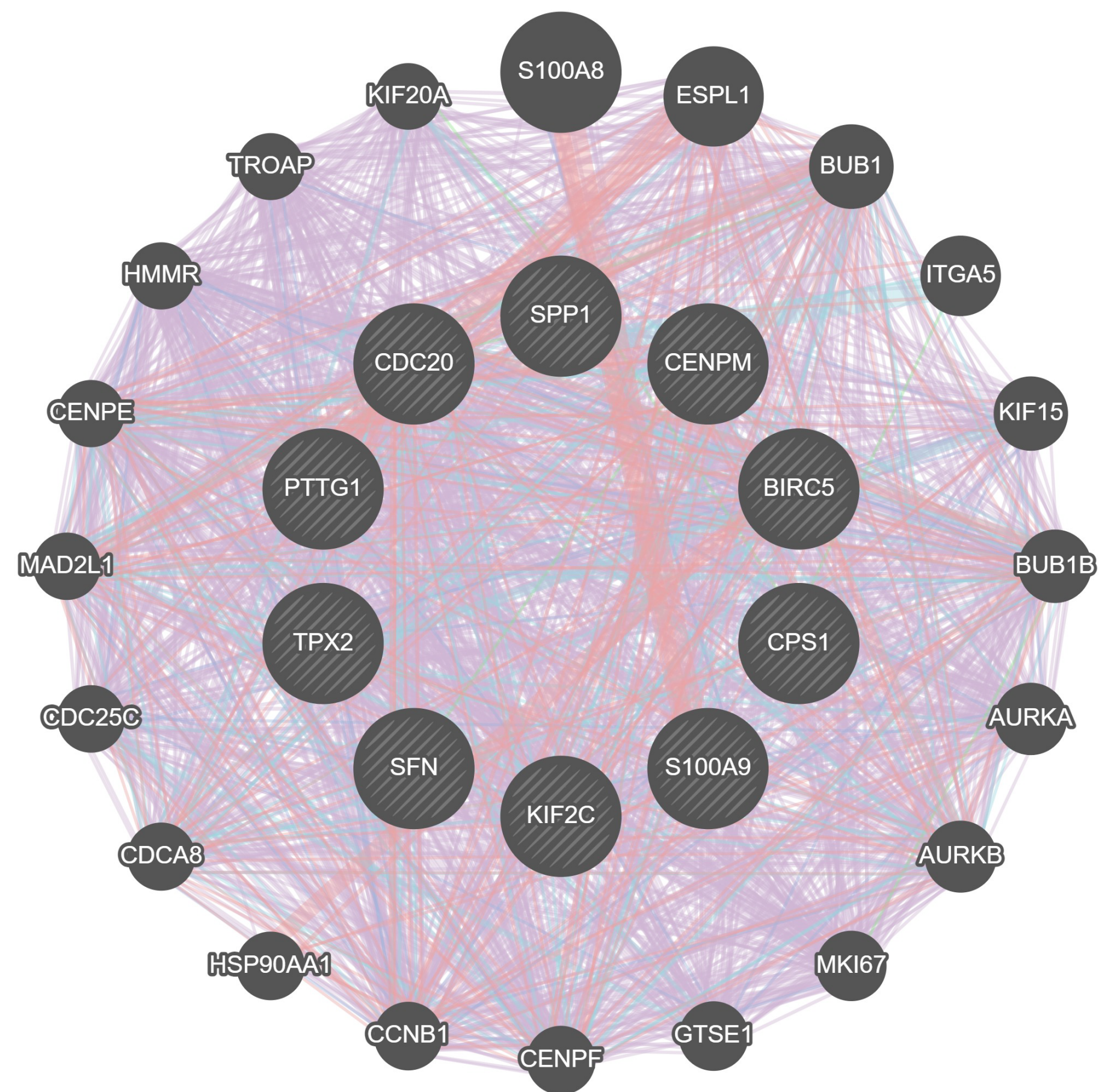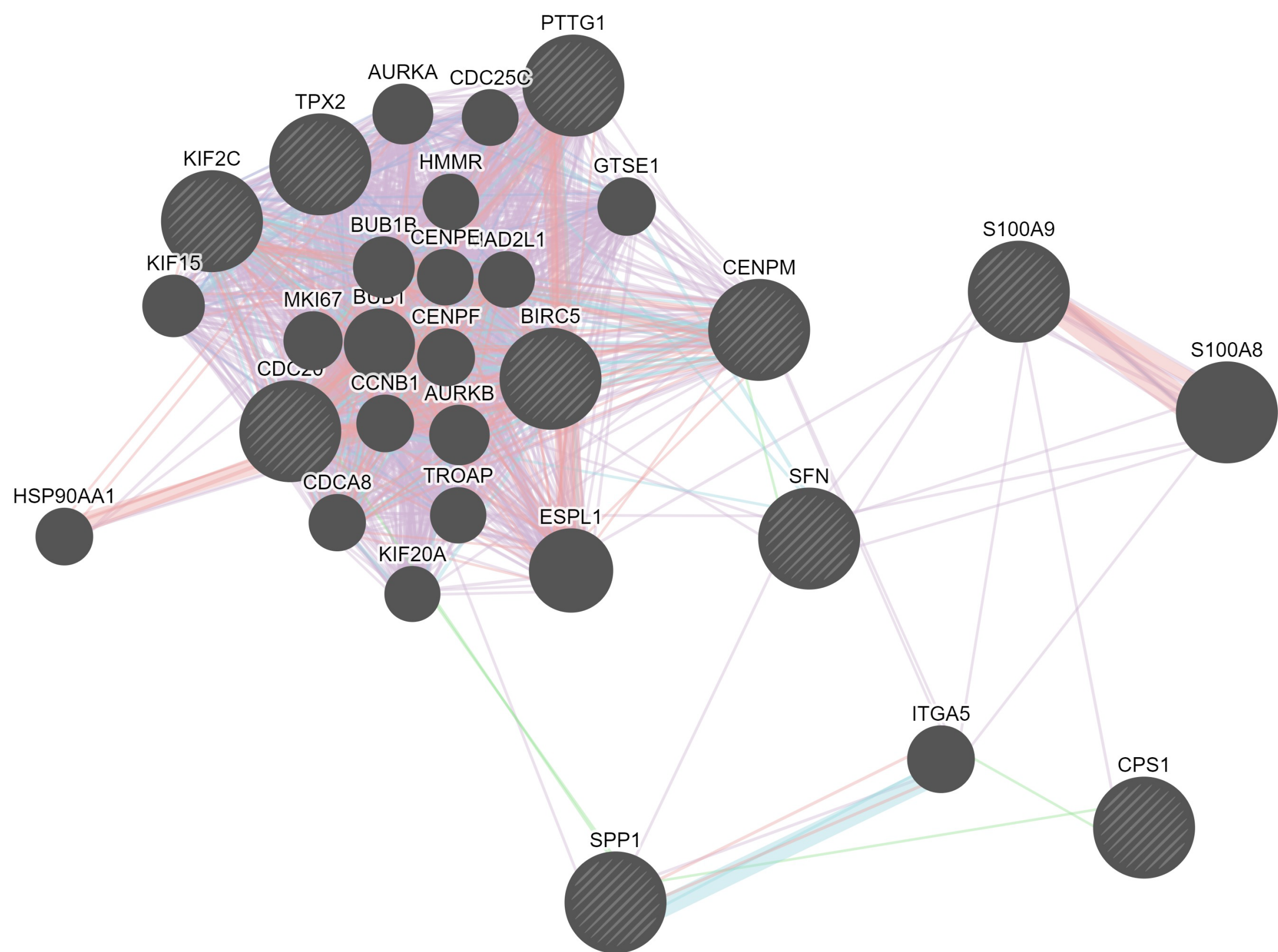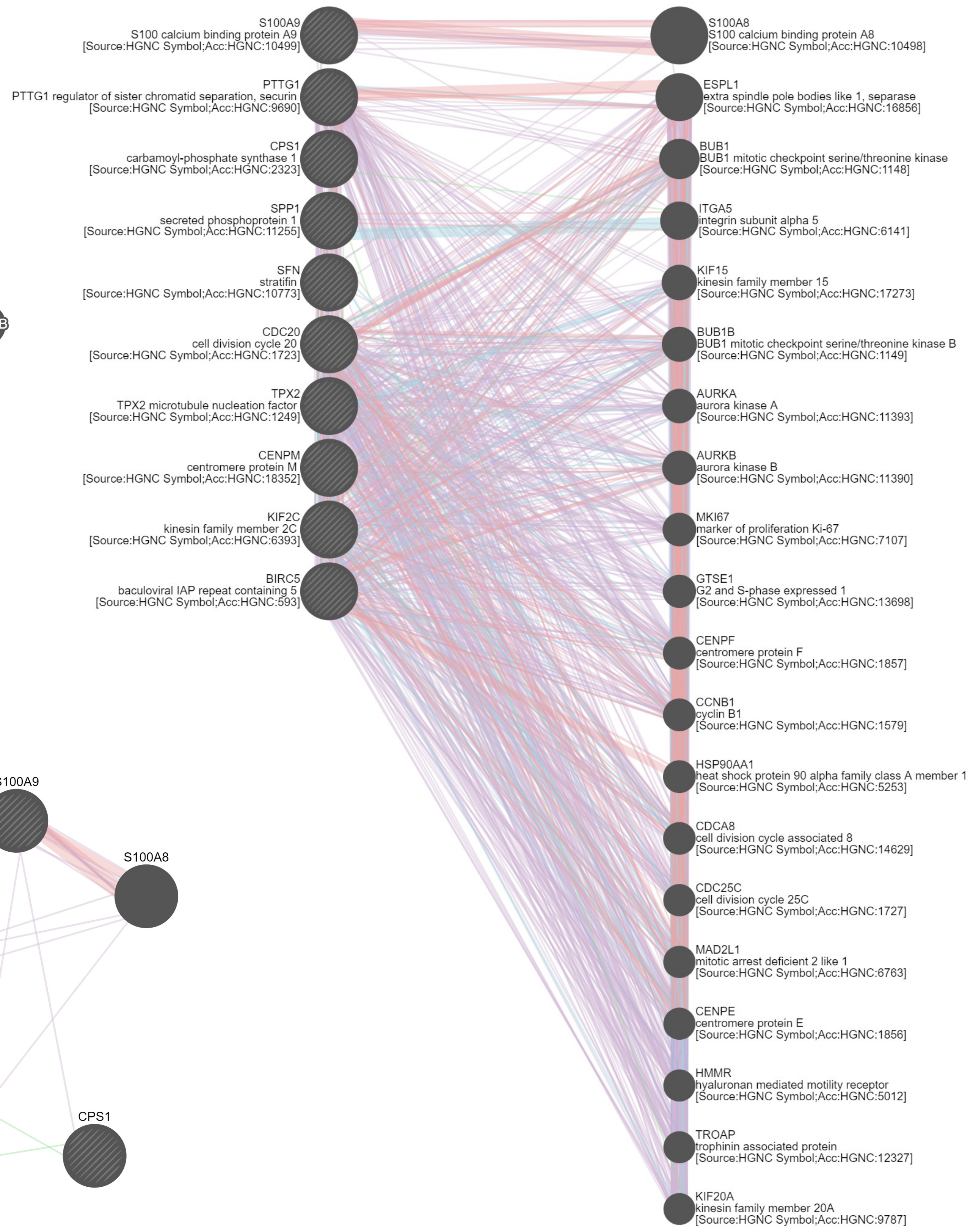

Supplement: Supporting Information — Additional supporting information can be found online in the Supporting Information section. Figure S1 Multiomics integrative consensus analysis based on the TCGA-LIHC cohort. (A) Evaluation of sample homogeneity through silhouette scores derived from consensus ensemble results. (B, C) PCA results before and after batch correction. (D) Results of DO terms enriched by 200 MS-related marker genes. Figure S2. Genomic landscapes between two HCC MSs. (A, B) GSVA scores for the hallmark gene sets and metabolism-related KEGG pathways. (C) Regulon activity profiles for 23 TFs and potential chromatin remodeling-associated regulators. (D) Immune checkpoint gene expression levels and ssGSEA scores of immune-related pathways. (E) Abundance of different immune cell types estimated by six independent algorithms. Figure S3. Development of MSRRS and its correlation with clinical characteristics. (A) PCA of training and validation cohorts before batch correction. (B) Detailed hazard ratios for 93 prognostic genes. (C) Results of bootstrap resampling of 93 prognostic genes. (D) Feature gene selection based on the Boruta algorithm. Green indicates genes considered important by the Boruta algorithm. (E) Correlation between MSRRS and clinical characteristics. (F, G) Univariate and multivariate Cox regression analysis of MSRRS and clinical characteristics. Figure S4. Molecular interaction networks associated with 10 MSRRS genes obtained from the GeneMANIA database. Figure S5. Correlation analysis of protein expression levels and CERES scores of potential therapeutic targets with MSRRS. (A) Protein expression. (B) CERES scores. Figure S6. Correlation between MSRRS and TME. (A) Differences in expression of various immunomodulators between high- and low-risk groups. (B) Correlation of MSRRS with predicted Treg cell abundance. (C, D) Differences in the activity of immune exclusion signatures and immunotherapy biomarkers between high- and low-risk groups. (E) Correlation of MSRRS with predict [file 9967779.f1.zip › Supply/Fig S4.pdf]

**A**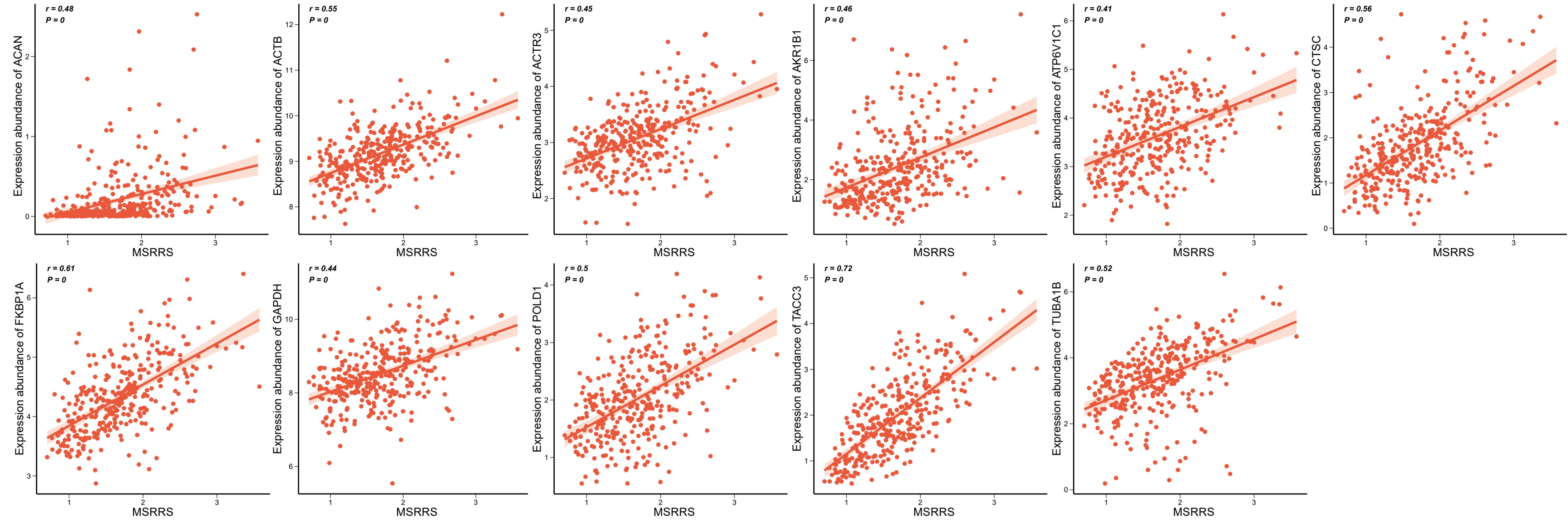**B**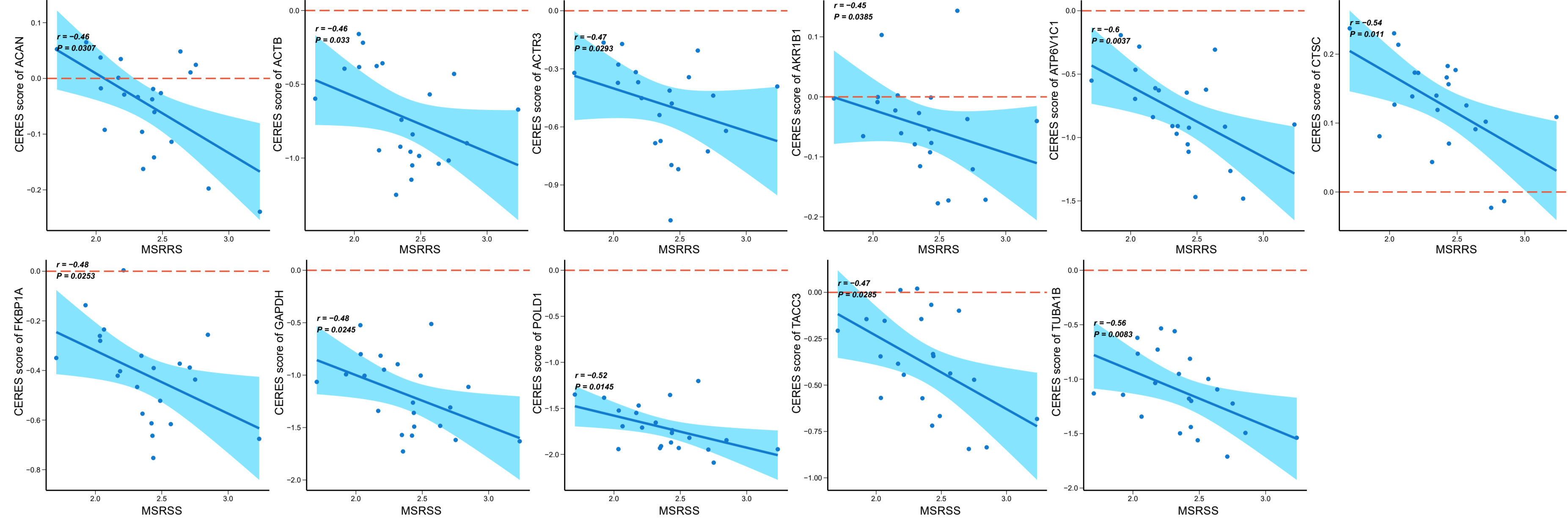

Supplement: Supporting Information — Additional supporting information can be found online in the Supporting Information section. Figure S1 Multiomics integrative consensus analysis based on the TCGA-LIHC cohort. (A) Evaluation of sample homogeneity through silhouette scores derived from consensus ensemble results. (B, C) PCA results before and after batch correction. (D) Results of DO terms enriched by 200 MS-related marker genes. Figure S2. Genomic landscapes between two HCC MSs. (A, B) GSVA scores for the hallmark gene sets and metabolism-related KEGG pathways. (C) Regulon activity profiles for 23 TFs and potential chromatin remodeling-associated regulators. (D) Immune checkpoint gene expression levels and ssGSEA scores of immune-related pathways. (E) Abundance of different immune cell types estimated by six independent algorithms. Figure S3. Development of MSRRS and its correlation with clinical characteristics. (A) PCA of training and validation cohorts before batch correction. (B) Detailed hazard ratios for 93 prognostic genes. (C) Results of bootstrap resampling of 93 prognostic genes. (D) Feature gene selection based on the Boruta algorithm. Green indicates genes considered important by the Boruta algorithm. (E) Correlation between MSRRS and clinical characteristics. (F, G) Univariate and multivariate Cox regression analysis of MSRRS and clinical characteristics. Figure S4. Molecular interaction networks associated with 10 MSRRS genes obtained from the GeneMANIA database. Figure S5. Correlation analysis of protein expression levels and CERES scores of potential therapeutic targets with MSRRS. (A) Protein expression. (B) CERES scores. Figure S6. Correlation between MSRRS and TME. (A) Differences in expression of various immunomodulators between high- and low-risk groups. (B) Correlation of MSRRS with predicted Treg cell abundance. (C, D) Differences in the activity of immune exclusion signatures and immunotherapy biomarkers between high- and low-risk groups. (E) Correlation of MSRRS with predict [file 9967779.f1.zip › Supply/Fig S5.pdf]

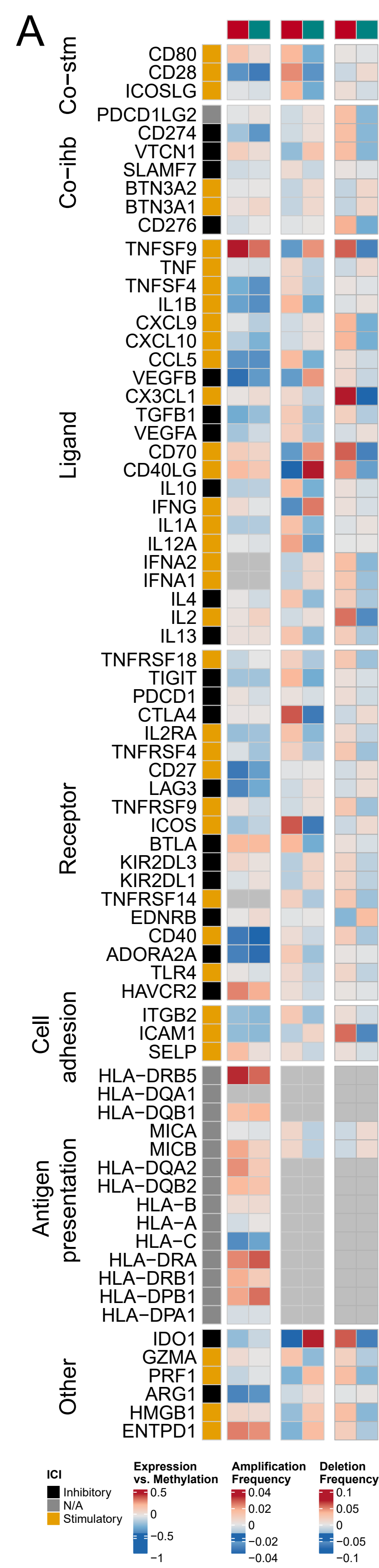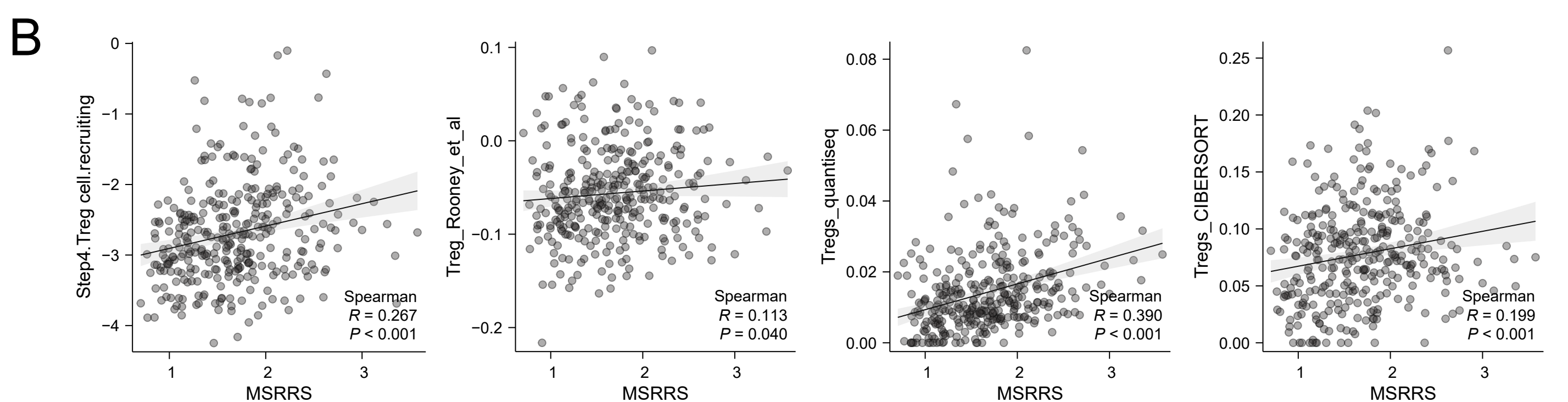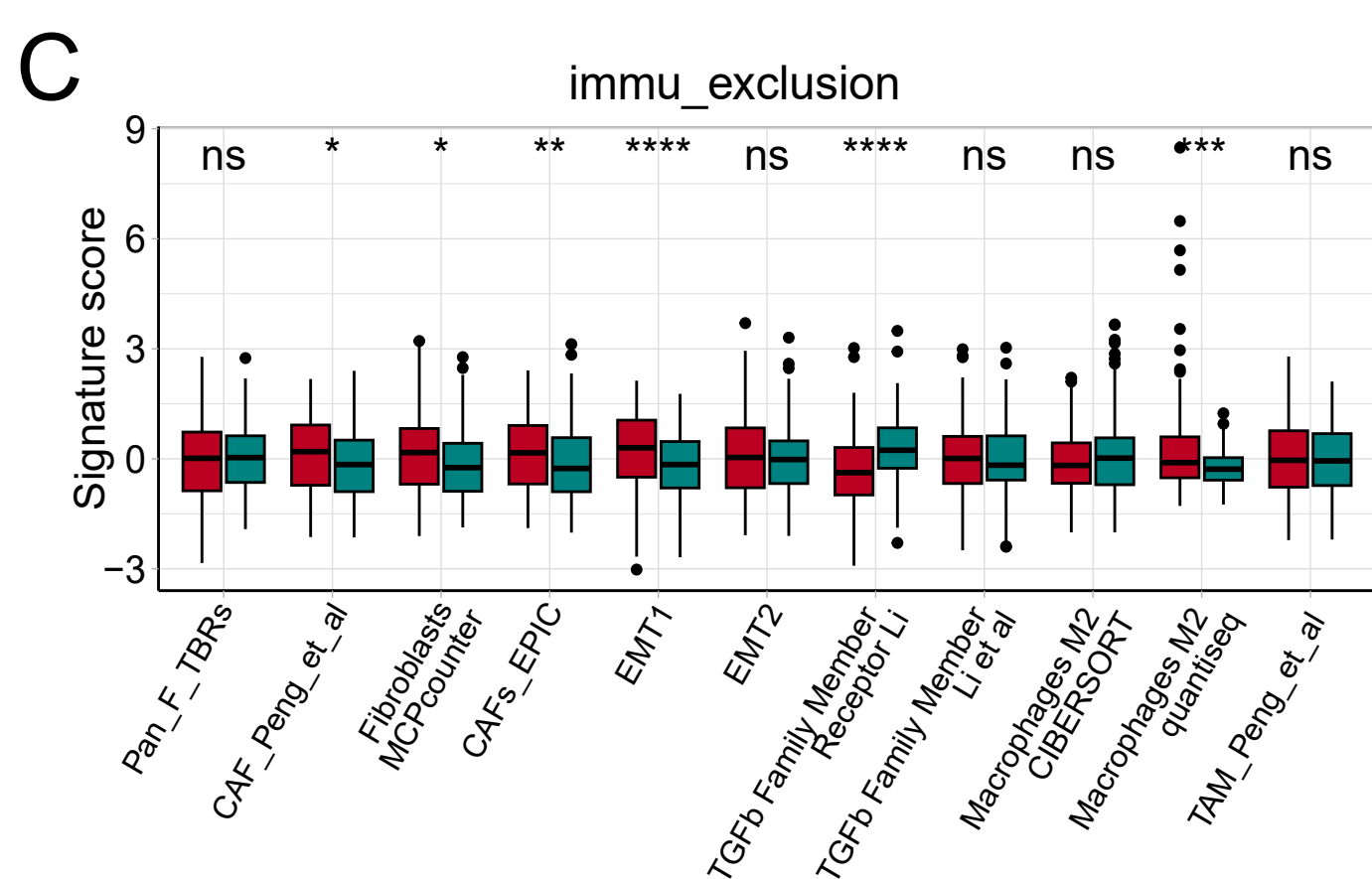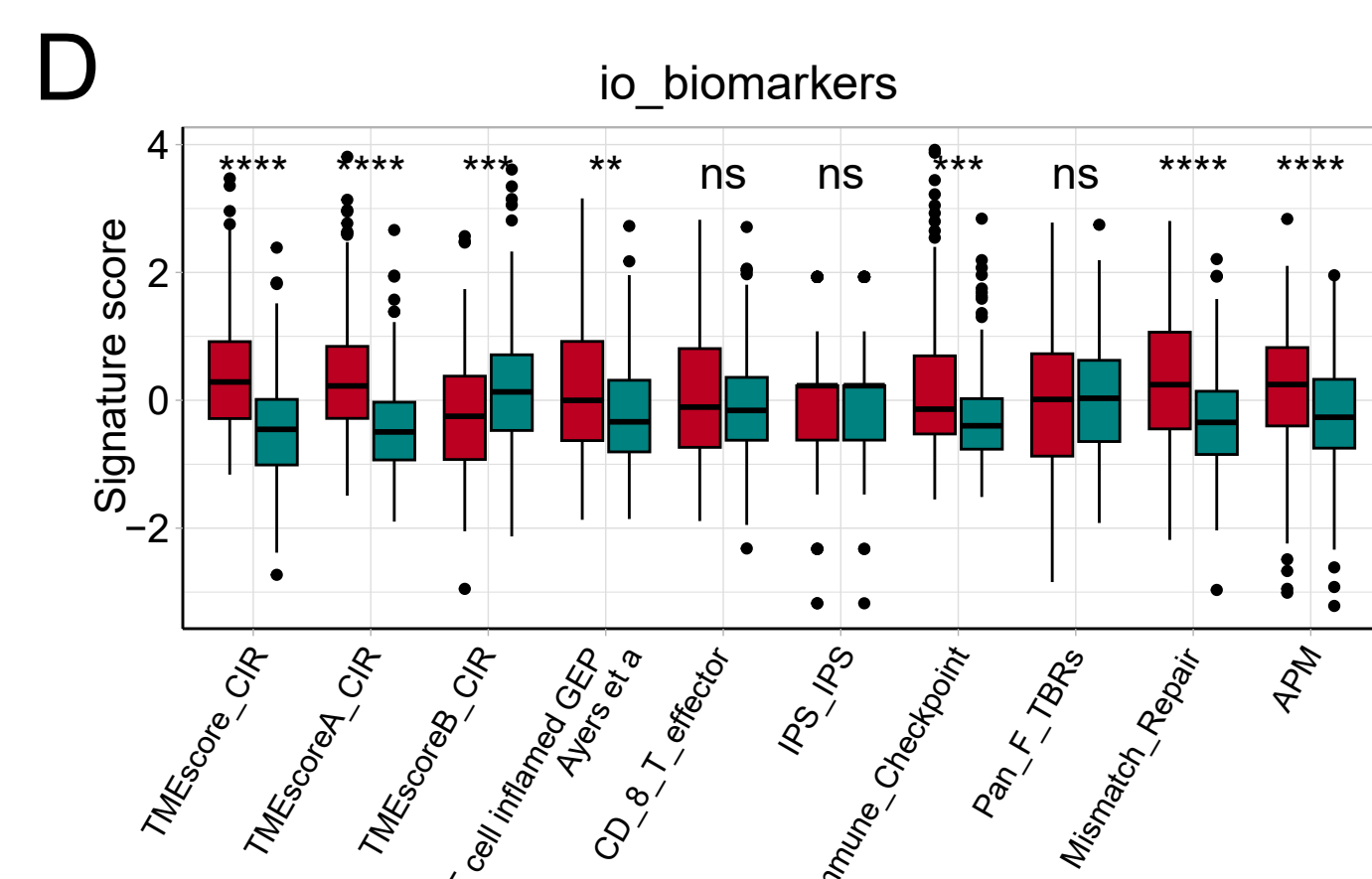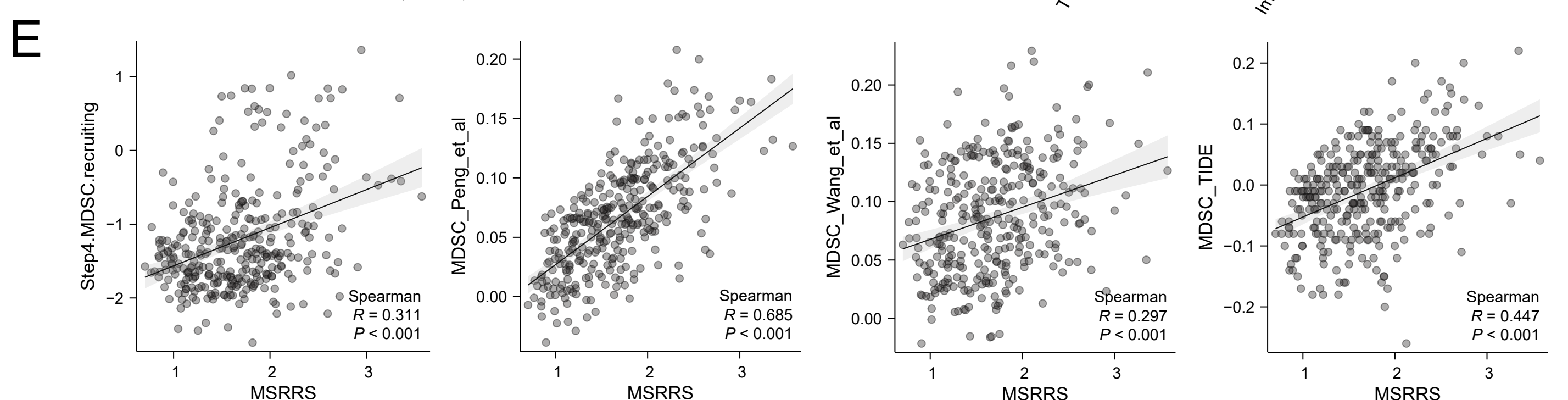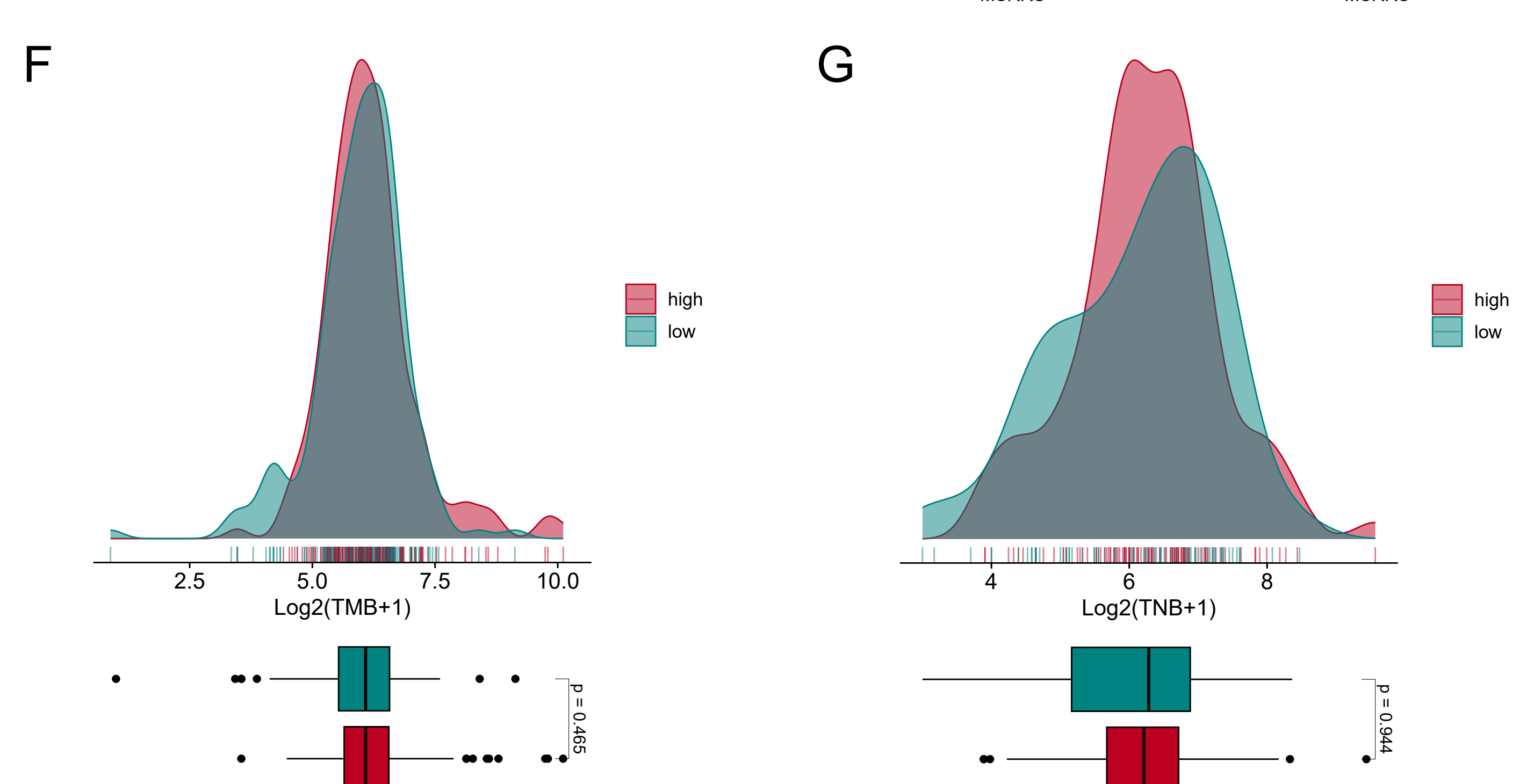

Supplement: Supporting Information — Additional supporting information can be found online in the Supporting Information section. Figure S1 Multiomics integrative consensus analysis based on the TCGA-LIHC cohort. (A) Evaluation of sample homogeneity through silhouette scores derived from consensus ensemble results. (B, C) PCA results before and after batch correction. (D) Results of DO terms enriched by 200 MS-related marker genes. Figure S2. Genomic landscapes between two HCC MSs. (A, B) GSVA scores for the hallmark gene sets and metabolism-related KEGG pathways. (C) Regulon activity profiles for 23 TFs and potential chromatin remodeling-associated regulators. (D) Immune checkpoint gene expression levels and ssGSEA scores of immune-related pathways. (E) Abundance of different immune cell types estimated by six independent algorithms. Figure S3. Development of MSRRS and its correlation with clinical characteristics. (A) PCA of training and validation cohorts before batch correction. (B) Detailed hazard ratios for 93 prognostic genes. (C) Results of bootstrap resampling of 93 prognostic genes. (D) Feature gene selection based on the Boruta algorithm. Green indicates genes considered important by the Boruta algorithm. (E) Correlation between MSRRS and clinical characteristics. (F, G) Univariate and multivariate Cox regression analysis of MSRRS and clinical characteristics. Figure S4. Molecular interaction networks associated with 10 MSRRS genes obtained from the GeneMANIA database. Figure S5. Correlation analysis of protein expression levels and CERES scores of potential therapeutic targets with MSRRS. (A) Protein expression. (B) CERES scores. Figure S6. Correlation between MSRRS and TME. (A) Differences in expression of various immunomodulators between high- and low-risk groups. (B) Correlation of MSRRS with predicted Treg cell abundance. (C, D) Differences in the activity of immune exclusion signatures and immunotherapy biomarkers between high- and low-risk groups. (E) Correlation of MSRRS with predict [file 9967779.f1.zip › Supply/Fig S6.pdf]

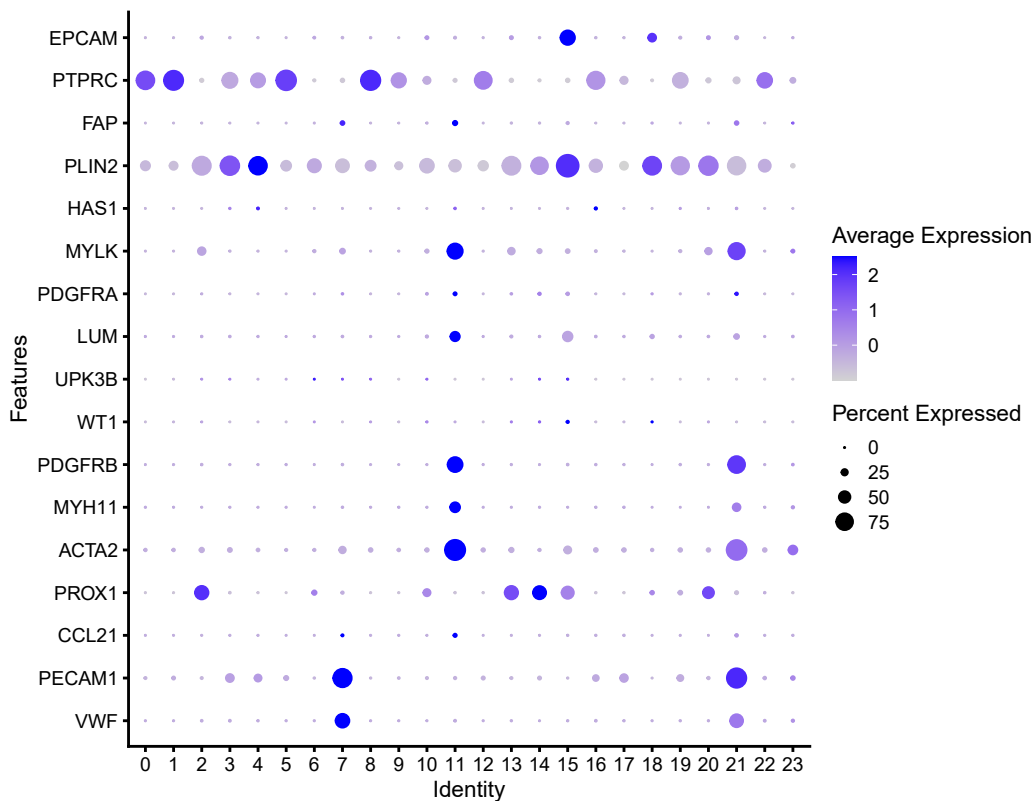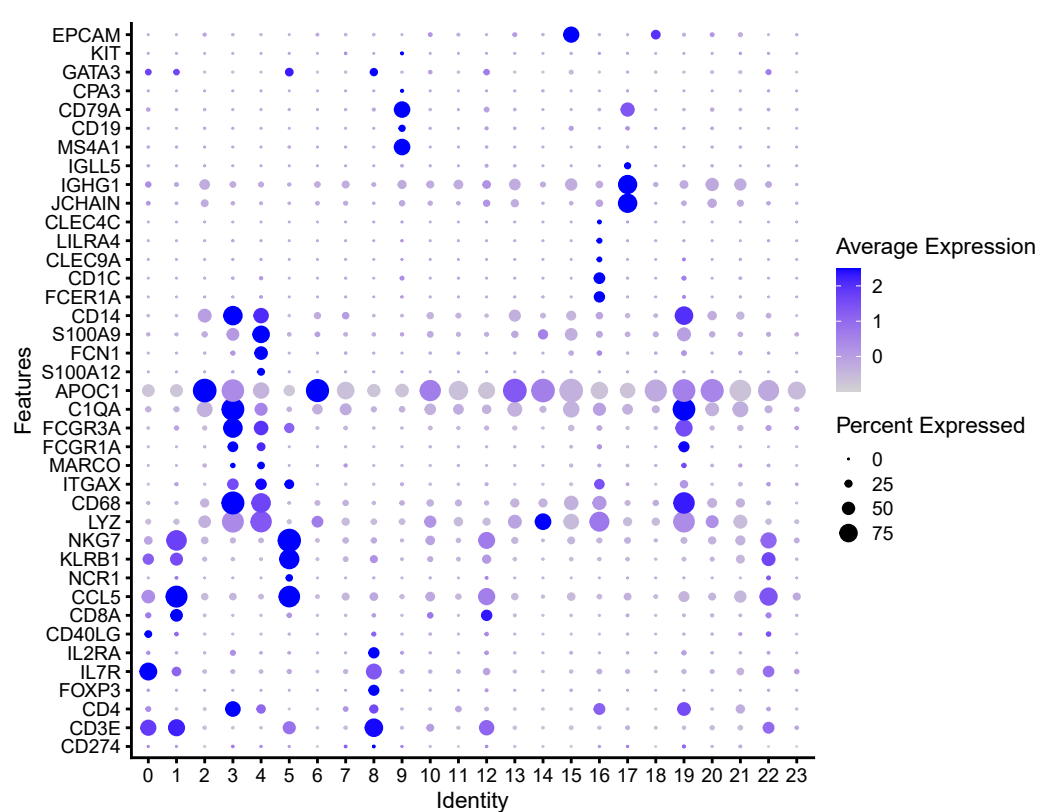

Supplement: Supporting Information — Additional supporting information can be found online in the Supporting Information section. Figure S1 Multiomics integrative consensus analysis based on the TCGA-LIHC cohort. (A) Evaluation of sample homogeneity through silhouette scores derived from consensus ensemble results. (B, C) PCA results before and after batch correction. (D) Results of DO terms enriched by 200 MS-related marker genes. Figure S2. Genomic landscapes between two HCC MSs. (A, B) GSVA scores for the hallmark gene sets and metabolism-related KEGG pathways. (C) Regulon activity profiles for 23 TFs and potential chromatin remodeling-associated regulators. (D) Immune checkpoint gene expression levels and ssGSEA scores of immune-related pathways. (E) Abundance of different immune cell types estimated by six independent algorithms. Figure S3. Development of MSRRS and its correlation with clinical characteristics. (A) PCA of training and validation cohorts before batch correction. (B) Detailed hazard ratios for 93 prognostic genes. (C) Results of bootstrap resampling of 93 prognostic genes. (D) Feature gene selection based on the Boruta algorithm. Green indicates genes considered important by the Boruta algorithm. (E) Correlation between MSRRS and clinical characteristics. (F, G) Univariate and multivariate Cox regression analysis of MSRRS and clinical characteristics. Figure S4. Molecular interaction networks associated with 10 MSRRS genes obtained from the GeneMANIA database. Figure S5. Correlation analysis of protein expression levels and CERES scores of potential therapeutic targets with MSRRS. (A) Protein expression. (B) CERES scores. Figure S6. Correlation between MSRRS and TME. (A) Differences in expression of various immunomodulators between high- and low-risk groups. (B) Correlation of MSRRS with predicted Treg cell abundance. (C, D) Differences in the activity of immune exclusion signatures and immunotherapy biomarkers between high- and low-risk groups. (E) Correlation of MSRRS with predict [file 9967779.f1.zip › Supply/Fig S7.pdf]

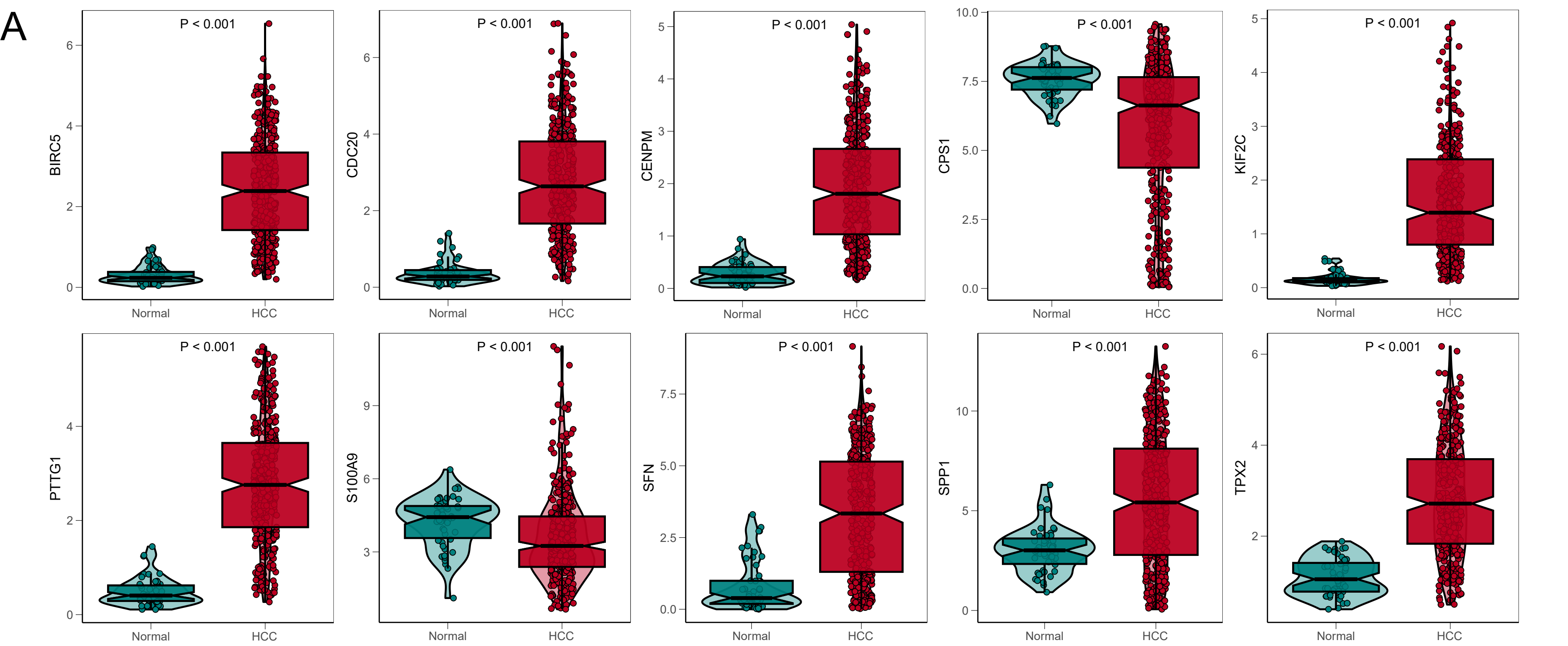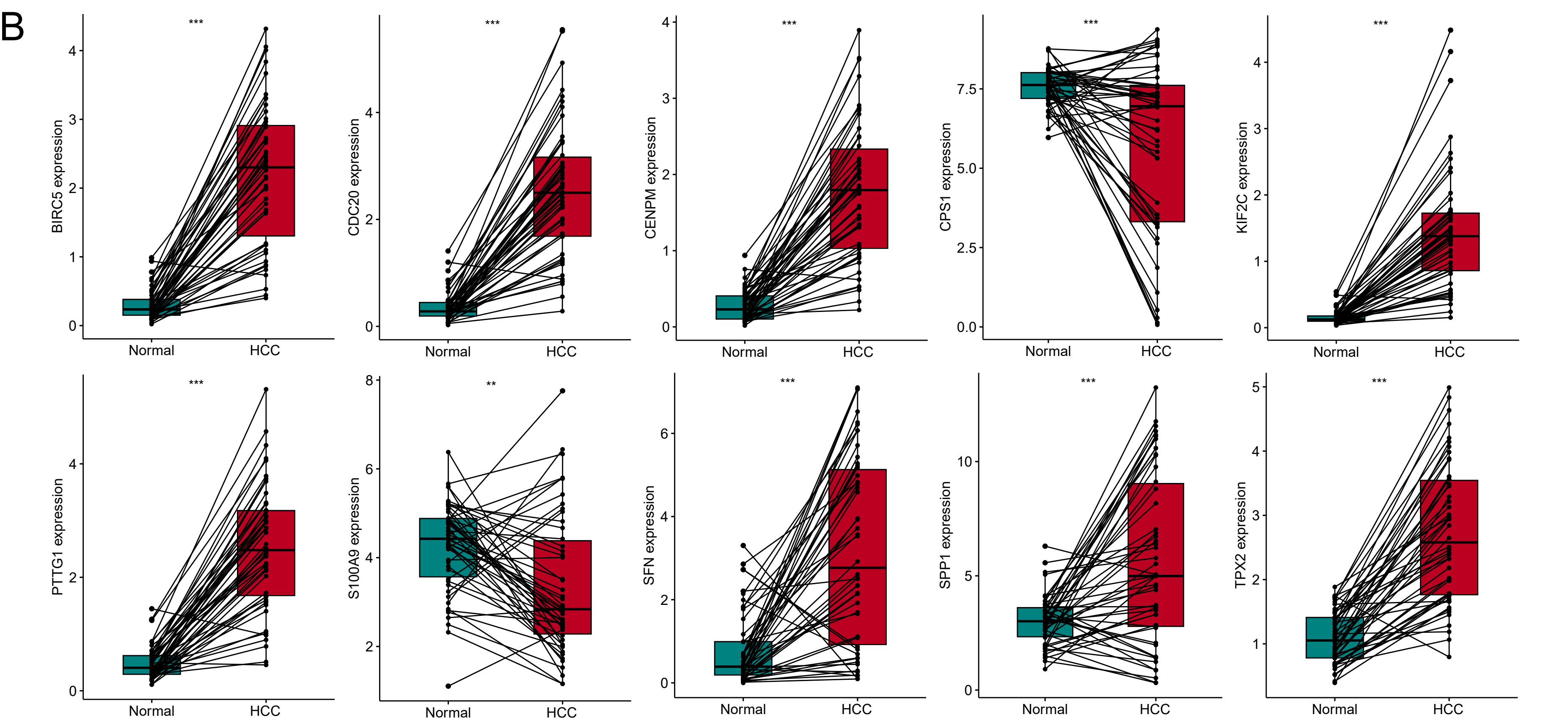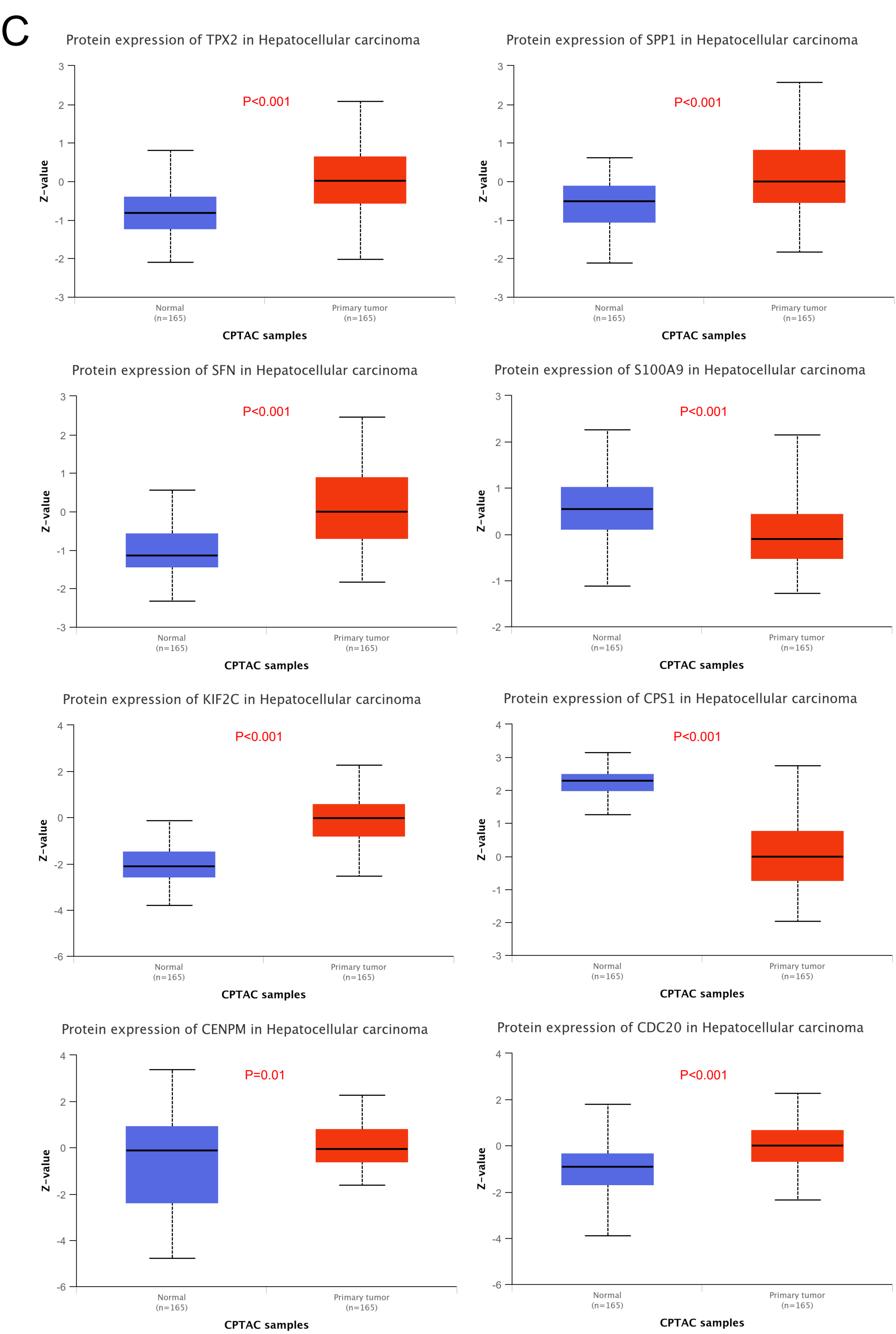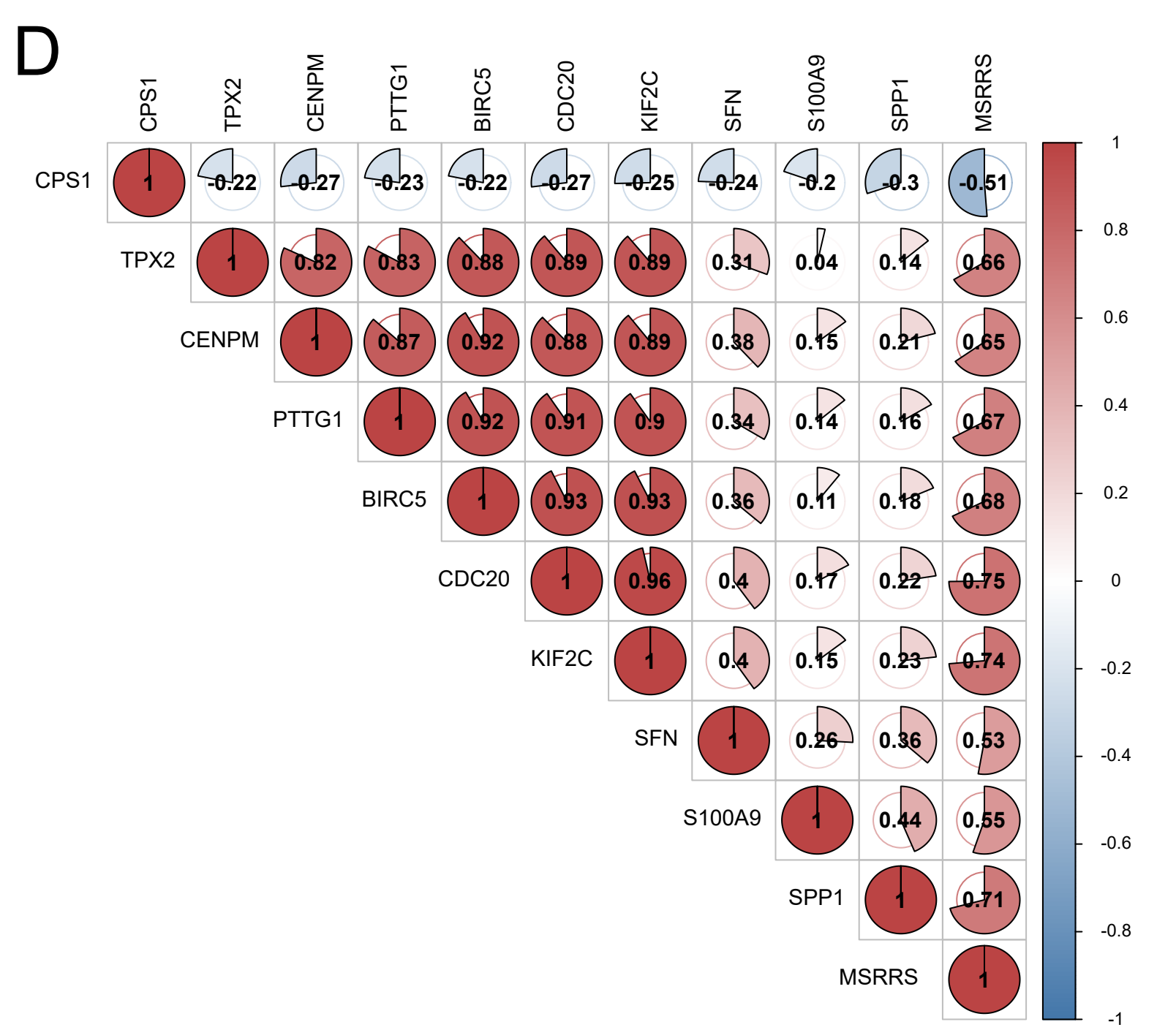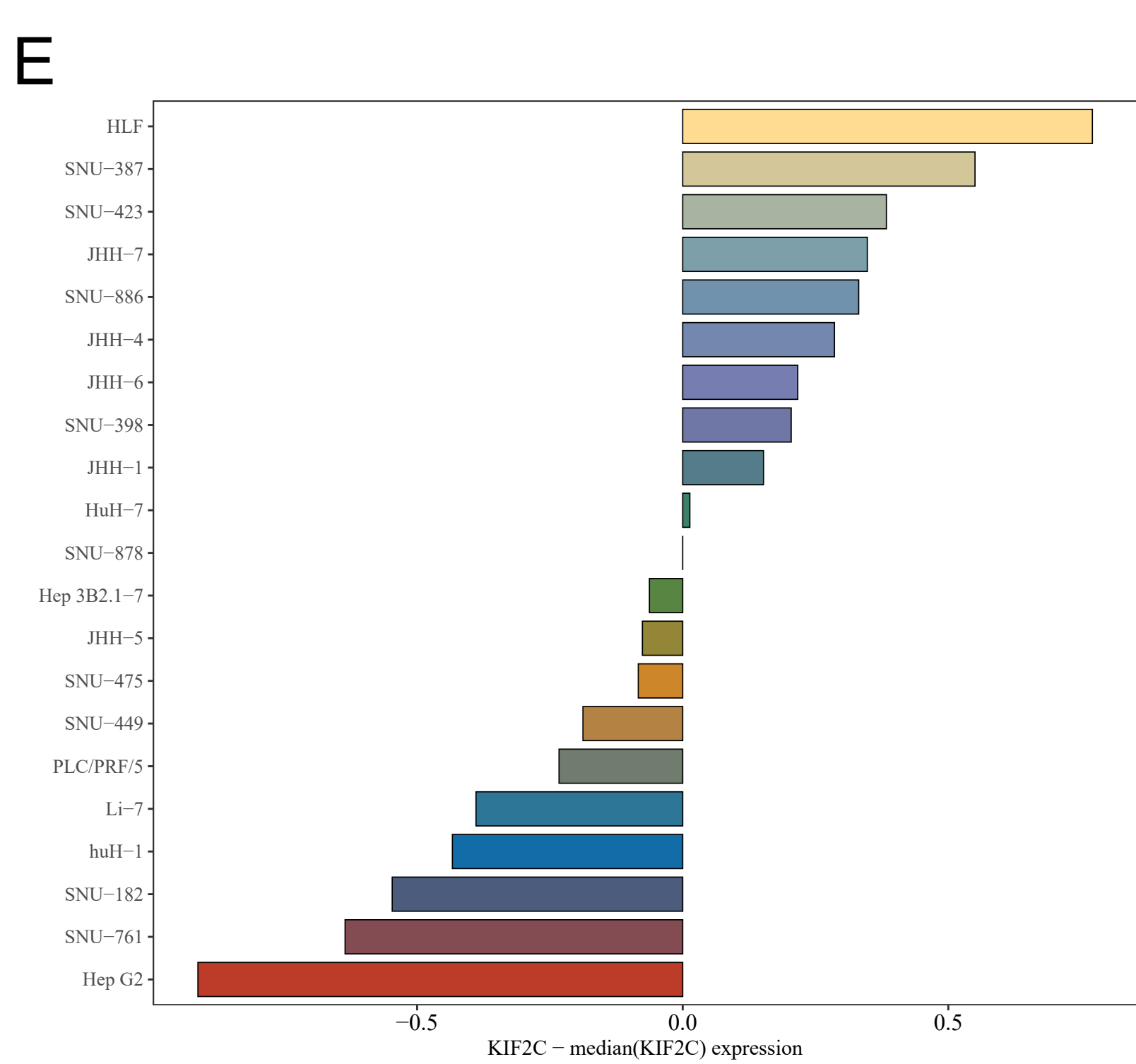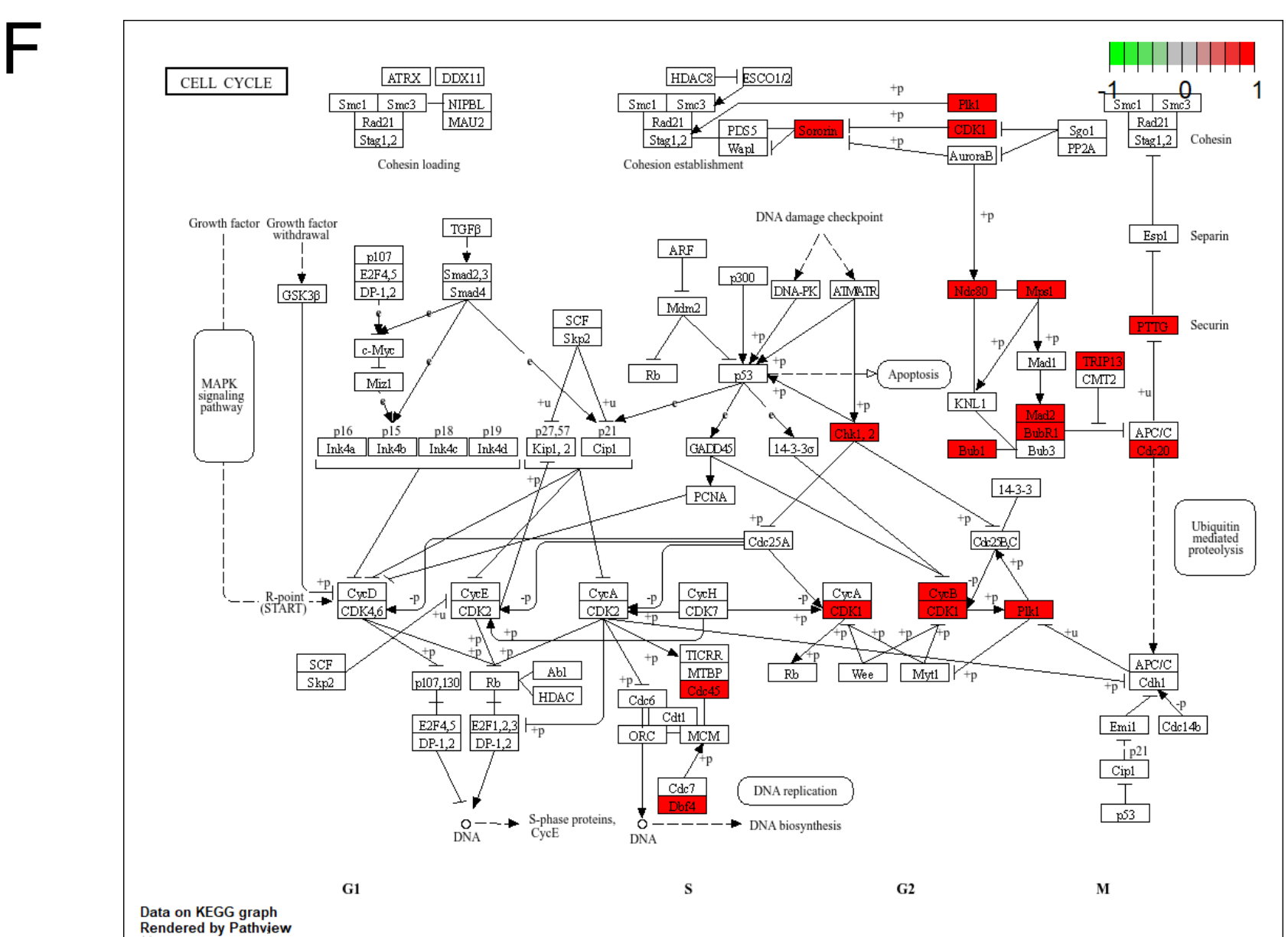

Supplement: Supporting Information — Additional supporting information can be found online in the Supporting Information section. Figure S1 Multiomics integrative consensus analysis based on the TCGA-LIHC cohort. (A) Evaluation of sample homogeneity through silhouette scores derived from consensus ensemble results. (B, C) PCA results before and after batch correction. (D) Results of DO terms enriched by 200 MS-related marker genes. Figure S2. Genomic landscapes between two HCC MSs. (A, B) GSVA scores for the hallmark gene sets and metabolism-related KEGG pathways. (C) Regulon activity profiles for 23 TFs and potential chromatin remodeling-associated regulators. (D) Immune checkpoint gene expression levels and ssGSEA scores of immune-related pathways. (E) Abundance of different immune cell types estimated by six independent algorithms. Figure S3. Development of MSRRS and its correlation with clinical characteristics. (A) PCA of training and validation cohorts before batch correction. (B) Detailed hazard ratios for 93 prognostic genes. (C) Results of bootstrap resampling of 93 prognostic genes. (D) Feature gene selection based on the Boruta algorithm. Green indicates genes considered important by the Boruta algorithm. (E) Correlation between MSRRS and clinical characteristics. (F, G) Univariate and multivariate Cox regression analysis of MSRRS and clinical characteristics. Figure S4. Molecular interaction networks associated with 10 MSRRS genes obtained from the GeneMANIA database. Figure S5. Correlation analysis of protein expression levels and CERES scores of potential therapeutic targets with MSRRS. (A) Protein expression. (B) CERES scores. Figure S6. Correlation between MSRRS and TME. (A) Differences in expression of various immunomodulators between high- and low-risk groups. (B) Correlation of MSRRS with predicted Treg cell abundance. (C, D) Differences in the activity of immune exclusion signatures and immunotherapy biomarkers between high- and low-risk groups. (E) Correlation of MSRRS with predict [file 9967779.f1.zip › Supply/Fig S8.pdf]

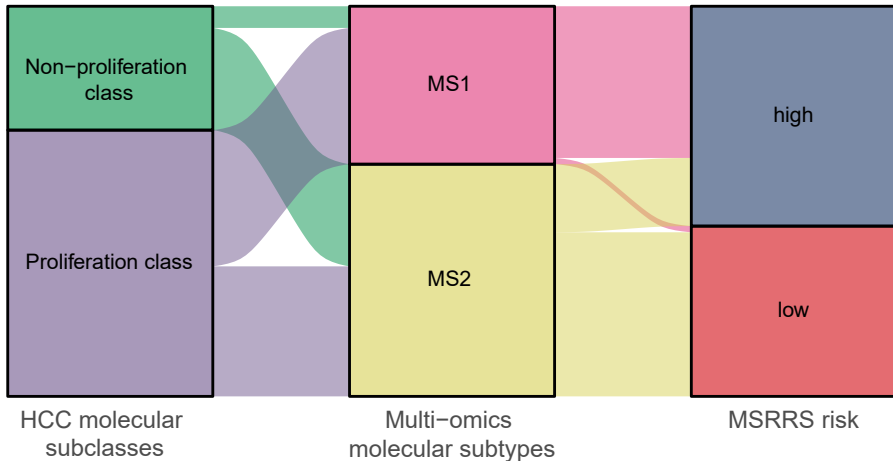

Supplement: Supporting Information — Additional supporting information can be found online in the Supporting Information section. Figure S1 Multiomics integrative consensus analysis based on the TCGA-LIHC cohort. (A) Evaluation of sample homogeneity through silhouette scores derived from consensus ensemble results. (B, C) PCA results before and after batch correction. (D) Results of DO terms enriched by 200 MS-related marker genes. Figure S2. Genomic landscapes between two HCC MSs. (A, B) GSVA scores for the hallmark gene sets and metabolism-related KEGG pathways. (C) Regulon activity profiles for 23 TFs and potential chromatin remodeling-associated regulators. (D) Immune checkpoint gene expression levels and ssGSEA scores of immune-related pathways. (E) Abundance of different immune cell types estimated by six independent algorithms. Figure S3. Development of MSRRS and its correlation with clinical characteristics. (A) PCA of training and validation cohorts before batch correction. (B) Detailed hazard ratios for 93 prognostic genes. (C) Results of bootstrap resampling of 93 prognostic genes. (D) Feature gene selection based on the Boruta algorithm. Green indicates genes considered important by the Boruta algorithm. (E) Correlation between MSRRS and clinical characteristics. (F, G) Univariate and multivariate Cox regression analysis of MSRRS and clinical characteristics. Figure S4. Molecular interaction networks associated with 10 MSRRS genes obtained from the GeneMANIA database. Figure S5. Correlation analysis of protein expression levels and CERES scores of potential therapeutic targets with MSRRS. (A) Protein expression. (B) CERES scores. Figure S6. Correlation between MSRRS and TME. (A) Differences in expression of various immunomodulators between high- and low-risk groups. (B) Correlation of MSRRS with predicted Treg cell abundance. (C, D) Differences in the activity of immune exclusion signatures and immunotherapy biomarkers between high- and low-risk groups. (E) Correlation of MSRRS with predict [file 9967779.f1.zip › Supply/Fig S9.pdf]
